# Supplementary material for: UPDATE trial: investigating the effects of ultra-processed versus minimally processed diets following UK dietary guidance on health outcomes: a protocol for an 8-week community-based cross-over randomised controlled trial in people with overweight or obesity, followed by a 6-month behavioural intervention
Source: BMJ Open. 2024 Mar 11;14(3):e079027. doi: 10.1136/bmjopen-2023-079027 (PMC10936475; doi:10.1136/bmjopen-2023-079027)
Supplement: Supplementary data [file bmjopen-2023-079027supp001.pdf]

Supplemental material BMJ Publishing Group Limited (BMJ) disclaims all liability and responsibility arising from any reliance placed on this supplemental material which has been supplied by the author(s) BMJ Open

PARTICIPANT INFORMATION SHEET

Sponsor Protocol Number: 151582
IRAS ID number: 311525

Study Title: UPDATE Trial: A study comparing the health effects of two diets following UK dietary guidance in people living with overweight or obesity

Name of Chief Investigator: Professor Rachel L. Batterham

Study team:
Professor Claudia Wheeler-Kingshott, Dr Adrian Brown, Dr Abigail Fisher, Dr Chris van Tulleken, Dr Janine Makaronidis, Dr Jed Wingrove, Tapiwa Ruwona, Samuel Dicken

Thank you for taking the time to read this participant information sheet (PIS). We would like to invite you to take part in our study investigating the benefit of eating a healthy, balanced diet as recommended by UK governments. The results from this study will be used to help inform UK dietary guidance and food policy. Before you decide if you would like to take part, it is important that you understand why this research is being done and what it will involve. Please take the time to decide whether or not you wish to take part. A member of the study team will call you in the next week to go through this information sheet with you and answer any questions you may have. You may also wish to discuss this information with your GP, your family or friends before deciding if you would like to take part.

Ask us if there is anything that is not clear. Take time to decide whether or not you wish to take part. We appreciate that this information sheet may not answer all of your questions, so please do not hesitate to contact a member of the study team on the telephone numbers given at the end of this information sheet if you would like to discuss any aspect of the study further.

Thank you for taking the time to read this information.

Dicken S, *et al. BMJ Open* 2024; 14:e079027. doi: 10.1136/bmjopen-2023-079027

Study summary:

- **UK governments recommend a healthy, balanced diet, which involves eating a variety of foods in the right proportions, such as five daily portions of fruit and vegetables, and limiting foods high in saturated fat, added sugar and salt.**
- **We want to see if the benefit of a healthy, balanced diet depends on the types of food processing in the diet.**
- **Staff at University College London Hospitals (UCLH) living with overweight or obesity will be invited to take part.**
- **There are two parts to this study, the first part is a diet intervention, the second part is behavioural support intervention to eat a healthier diet and be more active.**
- **In the first part, you will be given two diets in a random order, for 8 weeks each.**
- **Both diets are healthy and balanced, but differ in the types of food processing.**
- **After the first 8-week diet, you will return to your normal diet for 4 weeks, before we give you the second 8-week diet.**
- **We will not tell you which diet you will be given first.**
- **You will be given all your meals, snacks and drinks for both 8-week diets for free.**
- **After the second 8-week diet, we will then support you to eat a healthier diet and be more physically active for 6 months.**
- **We will collect data from you before you start both diets, at 4 weeks and 8 weeks into each diet, and then at the end of the 6-month support program.**
- **We will collect data about your health, including blood pressure, body composition, blood samples, physical activity and fitness, sleep and quality of life.**
- **The risk associated with taking part in the study is low, and no adverse events are expected from taking part.**
- **You can leave the study at any time, without giving a reason.**
- **You will be involved in the study for 49 weeks.**

Dicken S, *et al. BMJ Open* 2024; 14:e079027. doi: 10.1136/bmjopen-2023-079027

1. What is the purpose of the study?

Eating a healthy, balanced diet is important for our health. A healthy, balanced diet involves eating a variety of foods in the right proportions. In the UK, the Eatwell Guide gives us recommendations on what we should eat more of, and what we should eat less of. Unhealthy diets contain lots of foods high in saturated fat, added sugar and salt, which increases our risk of developing obesity, cardiovascular disease and type 2 diabetes. Advice for a healthy, balanced diet includes consuming five portions of fruit and vegetables per day and eating more high fibre foods. Following the healthy, balanced diet advice given in the Eatwell Guide can reduce our risk of poor health.

However, there are other parts of our diet that are not covered in the Eatwell Guide that may also be important for health. For example, research suggests that some types of food processing might influence our health, but these types of food processing are not included within the recommendations.

We do not know if the benefit of following the healthy diet advice in the Eatwell Guide depends on the types of food processing in our diets. It is important that we find out if these types of food processing matter, in order to give people the best advice possible. It also means that the government and other health organisations may need to change the regulations around the food we eat.

To answer this, we will compare the effects of two healthy, balanced diets following the advice in the UK Eatwell Guide, but each based on a different type of food processing. Participants will receive the first diet for 8 weeks, return to their normal diet for 4 weeks, and then receive the second diet for a further 8 weeks. They will have 6 months of support to help improve their diet and be more physically active. The results from this study will be used to help inform UK dietary guidance and food policy.

The study will also contribute towards the award of a PhD at University College London (UCL).

There are two parts to this study:

1. We will study if the benefit from eating a healthy, balanced diet depends on the types of food processing in the diet. We will do this by providing people with two diets that follow the Eatwell Guide, but containing foods with different types of processing, for 8 weeks each.
2. We will then study whether people are able to switch from their usual unhealthy diet to a healthy, balanced diet, and the benefits of doing so. We will do this by providing people with 6 months of personal support. We will also look at what helps people to maintain a healthy diet, and what makes it difficult. We will also support people to be more physically active.

2. Why have I been invited?

You have been invited to take part because you are a member of staff at UCL Hospitals (UCLH) living with overweight or obesity, and you have provided interest in participating in the study by contacting our research team.

However, you will be unable to take part if you have dietary restrictions that limit you from consuming the provided diets, such as having food allergies, or being vegan or vegetarian. You also cannot take part if you are pregnant and/or breastfeeding, or living with diabetes.

3. Do I have to take part?

No, the decision to take part is completely up to you. No one should force you into taking part. A member of the study team will contact you by telephone or email within a week of you receiving this invitation letter to see if you are still interested in taking part. We will describe the study and go through this information sheet. You can discuss any further questions you may have about the study with them. If you choose not to take part, that is completely fine. This will not affect any care you receive from the NHS, nor affect your role at UCLH. If you agree to take part, we will then ask you to sign a consent form. Even after signing the consent form, you are still able to withdraw from the study at any time without giving a reason. This would not affect the standard of any care you receive.

4. What will happen to me if I decide to take part?

If you decide to take part, you will be asked to sign two copies of the trial consent form. You will be given a copy to take home and keep. An appointment at a time and day convenient for you will be arranged for you to attend a screening visit. Signing the consent form however will not automatically enrol you onto the trial. Your eligibility to participate will depend on the data obtained during the screening visit. The research team will notify you on the outcome of this.

If you decide to take part, you will first be given one of the two diets for 8 weeks. You will then return to your normal diet for 4 weeks, before being given the second diet for another 8 weeks. After the second diet, you will be given 6 months of support to help you switch to and maintain a healthy, balanced diet and be more physically active.

For part 1 of this study, we will see if the benefit of consuming a healthy diet depends on the types of food processing in the diet. You will be given two diets in a random order, determined by a computer:

A. A diet following UK dietary guidance for 8 weeks containing foods based on one type of food processing
B. A diet following UK dietary guidance for 8 weeks containing foods based on another type of food processing

You will be given the diets in a random order, meaning that you could have diet A first then diet B, or diet B first then diet A. We will not tell you which diet you are on. Before the first diet, we will give you healthy eating advice from the Eatwell Guide, which the diets are based on. We will also explain to you about how to report how much of the food provided you have eaten. After the first 8-week diet, we will ask you to return to your normal diet you were eating before for 4 weeks. We will not provide your food or drink during this period. You will then receive the second diet for 8 weeks. After the second 8-week diet, we will stop providing you with food and drink, and you will then need to obtain your own food and drink. We will collect data on blood pressure, body composition, physical activity and fitness, questions regarding quality of life, mental health and

wellbeing, and blood samples at the start of each diet and at 4 and 8 weeks into each diet. We will work with you to find a suitable period of time to conduct part 1 of the study around any holidays or commitments you have.

The aim of part 1 is to collect important scientific data to see how food processing impacts on our health, rather than to assess specific foods. Therefore, we will provide you with all meals, snacks and drinks for the two 8-week diets, which have been designed with limited flexibility. You will not need to purchase any food or drink during this time. These will be set menus, featuring typical foods recommended in the Eatwell Guide that you might buy from a shop or make yourself at home. The meals and snacks on each menu will be varied across the week and include fruit, vegetables, beef, chicken, pork, fish, bread, potatoes, pasta, rice, noodles, oats, yoghurts, flapjacks, nuts, tea and coffee. We will not provide any alcohol. The diets will be delivered to your home at a time convenient for you or delivered to a safe space every few days. The meals will require minimal preparation, being ready to eat, or needing a few minutes in the microwave. Foods provided will need to be stored in either a fridge, freezer, or cupboard. We will provide a menu guide and instructions on how to prepare your food. Once prepared, you can then eat as little or as much of the food we give you as you like. We will give you kitchen scales and containers to prepare and store food for each day. You will have weekly telephone contact with the research team at a time that is convenient for you to help with any issues with the diets.

To provide you with your food for the diets, you will need to consent to and give your address and contact details to the food suppliers. This is explained in more detail in section 21.

Part 2 of the study will begin after you finish the second 8-week diet. A behavioural scientist will chat with you to create a personal plan to help you to eat a healthier diet and be more physically active. This support will last for 6 months, with ongoing monthly telephone/video calls with the research team to discuss your progress and support you. At the end of the 6 months, we will collect data on blood pressure, body composition, physical activity and fitness, questions regarding quality of life, mental health and wellbeing, and blood samples. We will also invite you to a one-to-one interview to chat about what aspects helped you to eat healthily and be more active, and what aspects made it difficult.

5. How many sessions do I need to attend?

You will be required to attend nine sessions:

- Screening visit

Part 1 of the study:

- Before the start of the first diet
- 4 weeks into the first diet
- 8 weeks into the first diet
- Before the start of the second diet
- 4 weeks into the second diet
- 8 weeks into the second diet

Part 2 of the study:

- At the start of the behavioural support program (this is a 60-90 minute remote telephone/video call)
- At the end (6 months) of the behavioural support program

Each visit will last 60-90 minutes except for screening, which will last around 30-40 minutes. These sessions will take place at UCLH or at the nearby Centre for Obesity Research (COR). We will conduct the visits at a time that suits you, that fits in with your work schedule and other commitments. If you are selected for MRI scans, this will be an extra 60 minutes at visits 2, 4, 5 and 7.

After all data is collected from visit 9 (the 6-month follow-up for the behavioural support), nothing more will be expected or asked of you, and you will have completed the study.

6. What data will be collected about me?

Screening

At the screening assessment, we will collect information to check that you are eligible and that it is safe for you to take part in the study. This will include:

- Checking that you do not have a medical diagnosis of an eating disorder.
- Checking your medical history to make sure that you do not have any medical conditions which might put you at risk during the study (such as Coeliac disease).
- Checking your current diet to see the amounts of different types of food processing in your diet.
- Checking that you do not have any food allergies or dietary restrictions that make it unsafe or not possible for you to eat the diets that we will provide you in part 1 of the study.
- If you are female, checking that you are not pregnant. We will ask you to take a urine pregnancy test. Urine taken for the pregnancy test will be disposed of following testing and not kept for any reason.

If you are not eligible, your information will be destroyed after 25 years.

During the study

The tests and data we will need to collect will include:

- Blood pressure and heart rate. We will measure blood pressure using a cuff that is placed on your upper arm. Heart rate will be measured with a small clip placed on your finger, called an oximeter.
- Weight and body composition, such as muscle mass and fat mass. You will stand on a scanner that uses an electric current to assess the amount of fat and muscle you have, for 30 seconds. Scales will be used to measure your weight, a stadiometer to measure your height, and a tape measure to measure your waist circumference.
- Blood samples before and after a meal. We will give you a standard meal to eat at our research centre. A 15ml tablespoon sized blood sample will be collected before and 15 minutes and 30 minutes after the meal, which will be performed by a trained healthcare professional. We will measure changes in the hormones that regulate your appetite, as well as markers of metabolism. Blood samples will be taken at five visits: before the start of the

Dicken S, *et al. BMJ Open* 2024; 14:e079027. doi: 10.1136/bmjopen-2023-079027

first and second diet, 8 weeks into the first and second diet, and at the end (6 months) of the behavioural support program. You will need to fast overnight for 12 hours before these five visits.

- How active you are during the day, and how much sleep you are getting. We will ask you to wear a monitor for 7 days to track your movement and sleep. The monitor is worn on your dominant hip or wrist for one week, from waking in the morning until going to bed at night. The monitor can be removed when taking a bath. We need a minimum of four days with at least 10 hours of daily wear time to collect useful data. The activity tracker records only basic movements, and does not record any identifiable information.
- How fit you are. We will ask you to complete three fitness tests. This will include squeezing a handgrip as hard as you can (handgrip strength test), measuring how far you can walk in 6 minutes (six-minute walk test), and testing your ability to stand from a seated position (sit-to-stand test).
- Questionnaires. We will ask you to fill in questionnaires about your physical activity, sleep, mental health, quality of life and eating behaviour, and what made it easier or harder for you to stick with your diet. The questionnaires will be provided to you in an electronic format, and can be completed at the study visit or remotely.
- Dietary information. To keep track of what you are eating, we will ask you to recall what you have eaten in a 24-hour period for two days, and ask you to fill in a questionnaire that asks how frequently you have eaten certain foods. We will explain how to complete these. We will ask you to also take some photos of your prepared meals for a couple of days during the 4- and 8-week follow-up visits in part 1 of the study. If it helps, we can also provide you with a diet diary to keep track of what you eat during each diet.
- A 60-90 minute interview at the end of the study to discuss the 6-month support program. A trained behavioural scientist will chat with you about the aspects of your personal, social and work life that helped you to stick with a healthy diet, and what aspects of your life made it difficult. This interview will be audio recorded and then converted into text. Some of the things you say may be quoted verbatim, but this will be anonymised before being published, so it will not be possible to identify you. Interviews will take place via telephone or video call at a time convenient for the participant during the scheduled visit time. Transcriptions will be anonymised following transcription, and audio recordings deleted as soon as transcriptions of the recordings have been obtained.

For some participants, we will also collect:

- Brain imaging scans. Not all participants will undergo a brain imaging scan. You may be selected to receive a brain scan if you consent, but you can opt out of this on the consent form. You will have a magnetic resonance imaging (MRI) scan on your brain. MRI does not expose you to ionising radiation. Brain imaging will be conducted before you start each diet, and at the 8-week visits for each diet. The scans will last around 60 minutes for each visit, in addition to the 60-90 minute visits. Scans will be conducted at the Institute of Neurology next to Russell Square. We can book these scans around your schedule at a separate visit if necessary. If you do not wish to undergo brain imaging if chosen, you can opt out on the consent form. We will provide you with more information about the brain scans at the screening visit. The brain is important for determining what we eat. Different areas of the brain can impact on the types of foods we eat, and evidence suggests that connections

between these different areas of the brain may change whilst eating certain diets. We want to learn more about how connections between different brain areas change when eating two different diets.

7. How long will I be involved in the study for?

After signing the written informed consent form, it is expected that your involvement in the study will last for around 49 weeks. Part 1 of the study will last around 24 weeks. Part 2 of the study will last around 25 weeks.

8. Study Timeline

The study timeline is outlined below.

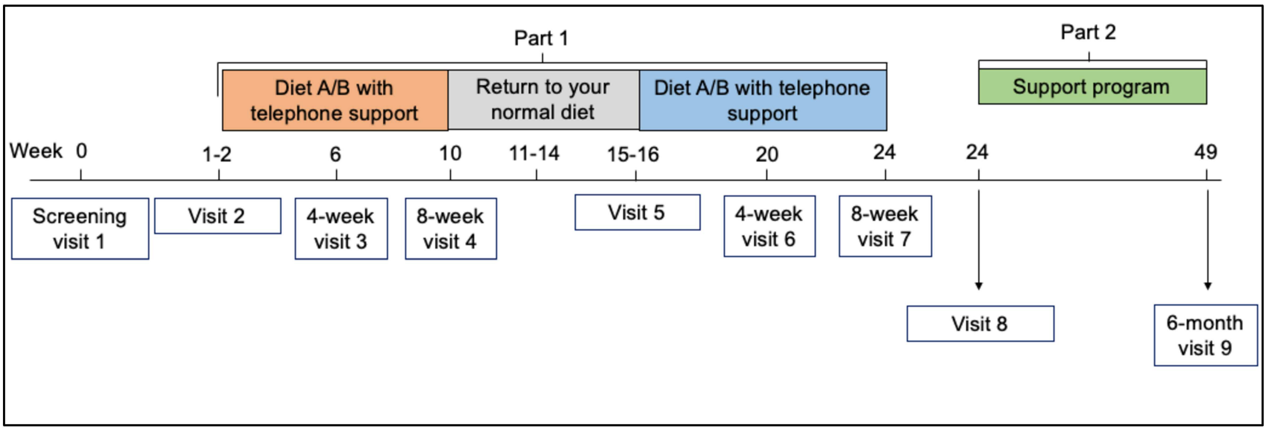

Assessment visit schedule and data collection

The table below outlines the measures collected at each visit and what will happen during the study.

		Part 1						Part 2	
		First diet			Second diet				
	Screening	Before start of diet	At 4 weeks	At 8 weeks	Before start of diet	At 4 weeks	At 8 weeks	At the start of support program	At the end of support program
**Visit Number:**	1	2	3	4	5	6	7	8	9
**Week:**	0	1-2	6	10	15-16	20	24	24	49
Informed Consent for Screening	Y								
Medical history	Y	Y	Y	Y	Y	Y	Y		Y

Dicken S, *et al. BMJ Open* 2024; 14:e079027. doi: 10.1136/bmjopen-2023-079027

Heart rate and blood pressure	Y	Y	Y	Y	Y	Y	Y		Y
Urine Pregnancy Test (females only)	Y								
Body composition		Y	Y	Y	Y	Y	Y		Y
Blood samples before and after a meal: you will need to fast for 12 hours before this visit		Y		Y	Y		Y		Y
Physical activity and sleep monitor		Y		Y	Y		Y		Y
Physical Fitness Tests		Y	Y	Y	Y	Y	Y		Y
Questionnaires		Y	Y	Y	Y	Y	Y		Y
Dietary information	Y	Y	Y	Y	Y	Y	Y		Y
Brain scans (if chosen)		Y		Y	Y		Y		
Behavioural support call								Y	
One to one telephone/video call interview									Y

9. Where will the study take place?

The study will take place at the Centre for Obesity Research (COR) at UCL and at UCLH. If selected for brain scans, these will be at the nearby UCL Institute of Neurology. You will not need to travel to any other location at any other time.

10. What will I have to do?

If enrolled in the study, we will expect you to attend all scheduled ither in-person or remote study visits, and perform the tests and assessments as listed above, accordingly to the study protocol. Importantly, you will be asked to prepare and eat the diets provided to you in part 1, and not to consume other foods or drinks during each 8-week period. The research team will be available if you need to contact them, for example, if there are issues with adherence due to social events,

issues with the provided diets (e.g. a missing delivery or missing meals) or problems with dietary reporting.

11. What are the possible disadvantages and risks of taking part?

Possible disadvantages and risks are related to the study assessments, procedures and questionnaires you will have to undergo when taking part in the trial. However, these would be considered minor risks/disadvantages, as explained below. The study is generally low risk. We do not expect there to be any major risks or adverse events from taking part in the study. There are no expected risks from the screening visit.

The two 8-week diets that we will provide you in part 1:

- As part of the screening process, we will make sure that you do not have any allergens or dietary restrictions to the foods we will provide you in part 1. We will check that there are no potential concerns regarding your eating behaviour.
- We will provide you with simple instructions on how to safely prepare and store your meals, and you will not be expected to consume foods beyond their best by date or use by date.

Blood samples before and after a meal:

- Taking blood samples may occasionally cause a bruise or pain. All samples will be obtained by a trained healthcare professional.

Physical fitness tests:

- The physical fitness measures are not expected to result in a risk of injury. Trained researchers will provide full instructions and supervision for the tests.

Questionnaires:

- You might find some of the questions about your mental health and quality of life upsetting. However, you can stop the questionnaires at any time, or decline to answer specific questions, without having to give a reason or without affecting your rights. If you do feel upset and you feel you would benefit from some emotional support, you can contact the Samaritans charity (https://www.samaritans.org) on 116 123, their helpline is open 24 hours per day. You can also contact MIND (https://www.mind.org.uk), or the CALM (Campaign against living miserably) charity, who have a helpline open from 5pm to midnight every day on 0800 58 58 58 or a web chat (https://www.thecalmzone.net).

Support program:

- In order to support you with eating a healthy diet and being more physically active, the behavioural scientist may discuss aspects of your personal, social and work life that may be upsetting. The aim of the discussion is to help you, such that there is a positive impact on your health. As with the questionnaires, you can stop the discussions at any time, or decline to answer specific questions, without having to give a reason or without affecting your rights.

Brain scans:

- If you consent and are selected for brain imaging assessment (unless you choose to opt out on the consent form), you will undergo an MRI scan. MRI does not expose you to ionising radiation. But, the noise from the scanner and small space can cause stress and discomfort.
- Our researchers will check that you are suitable to undergo MRI brain scanning during screening. We will also check for any reasons why you cannot undergo an MRI scan, which will be confirmed prior to every scan.

Unexpected findings

In the unlikely event that we notice an abnormal finding in some of the measurements we take (including blood tests, heart rate, blood pressure and brain scans), we will inform you and your GP, who will discuss the findings and any further action needed with you.

Will I experience any side-effects?

We do not expect there to be any side effects from the diet interventions, from the support program, or from the tests involved.

The diets provided contain typical foods in UK diets and are obtained from supermarkets or made in a professional catering facility.

It is important that if you do feel any unusual symptoms, regardless of whether you think they are linked to the study, that you tell a member of the study team.

12. What are the possible benefits of taking part?

It is likely that you will experience a benefit from the 6-month support program as you will be supported to improve your diet and be more physically active. It is not possible to guarantee any specific benefit from part 1 of the study, where we provide you with two diets.

The knowledge gained from this study will provide further information into whether a healthy diet depends on how processed it is. Results from this study can be used to inform changes to the current recommendations for dietary guidelines. A greater understanding of the long-term risks of diets can better inform healthcare professionals on helping people living with obesity.

13. How many participants will be in the study?

We will recruit 55 members of staff at UCLH living with overweight or obesity. The study will run for approximately 18 months in total.

14. What happens when the trial stops?

We will only provide your food and drink for the two 8-week diets in part 1 of the study. We will not be able to provide behavioural support after the end of the study.

At the end of the trial, we will analyse the data and publish the results in medical journals, and present results at scientific conferences. If you wish, a copy of the reports on the study findings can be requested from the Chief Investigator, Professor Batterham, at the address given in section 19 of this information sheet. Should the study be stopped prematurely for any reason, we will tell you why.

15. Will my taking part in the study be kept confidential?

Yes. We will follow ethical and legal practices and all information about you will be handled in confidence. The details are outlined in the following section 19.

16. Will I be paid to take part in the study?

You will receive the two 8-week diets for free in part 1 of the study. This means you will not need to purchase any food or drink for 16 weeks. We will not directly pay you for any aspects of the study.

We do not foresee any travel costs, as all study measurements will be at UCLH or the adjacent COR, so you will not have to make any extra journeys or travel anywhere other than to your usual appointment location. If you do need to travel specifically for the study visits, any reasonable travel costs incurred to attend the study visit will be reimbursed on production of valid receipts. All the food we provide will be delivered to your home every few days, so you do not need to travel to collect your food. We will work with you to ensure food deliveries, meetings or assessments align with your work and other commitments.

None of the study team are being paid beyond their normal salary to conduct the research, nor are any clinicians being paid for recruiting individuals into the study.

17. What if relevant new information becomes available?

Sometimes during a clinical trial, new information about the intervention becomes available. If this happens, we will tell you about it and discuss with you whether you want to or should continue your participation in this study. If you decide not to carry on, we will make arrangements for your care to continue. If you decide to continue in the study, you might be asked to sign an updated consent form. Also, on receiving new information, we might consider it to be in your best interest to withdraw from the study. If so, we will explain the reasons and arrange for your care to continue.

18. What will happen if I don’t want to carry on with the study?

Your participation in the trial is entirely voluntary and you will be free to withdraw from the study at any time and without giving a reason. A decision to withdraw will not affect the current or future care you receive. If you withdraw from the study, we will destroy all your identifiable information, but we will need to use and analyse the pseudonymised data collected up to the point of your withdrawal. You will be provided with a withdrawal form where you will be able to express your preference. In any case, pseudonymised (coded) information collected up to the point of your

Dicken S, et al. BMJ Open 2024; 14:e079027. doi: 10.1136/bmjopen-2023-079027

withdrawal will still be used in the analysis. Any stored blood or tissue samples that can still be identified as yours will be destroyed if you wish.

19. What if there is a problem?

If you have any problem during the study, or would like to discuss any aspect of the study, you can contact any of the research investigators. You can find their contact details on the last page of this information sheet. Every care will be taken in the course of this clinical trial.

However, in the unlikely event that you are injured by taking part, compensation may be available through the University College London (UCL) insurance scheme. If you suspect that the injury is the result of the Sponsor's (University College London) or the hospital's negligence then you may be able to claim compensation. After discussing with your research doctor, please make the claim in writing to the Chief Investigator for this trial:

Professor Rachel Batterham,
Centre for Obesity Research,
Rayne Building,
5 University Street,
London, WC1E 6JF.

The Chief Investigator will then pass the claim to the Sponsor's Insurers, via the Sponsor's office.

However, if you remain unhappy or have a complaint about any aspect of this study and wish to speak to someone independent of the research team/hospital, please contact the Head of Research Governance and Compliance, UCL/UCLH Joint Research Office, University College London, Gower Street, London WC1E 6BT email: research-incidents@ucl.ac.uk.

Regardless of this, if you wish to complain, or have any concerns about any aspect of the way you have been approached or treated by members of staff or about any side effects (adverse events) you may have experienced due to your participation in the clinical trial, the normal National Health Service complaints mechanisms are available to you. You could also write or get in touch with the Complaints Manager, UCL hospitals. Please quote the study IRAS number at the top of this information sheet. Please ask your study doctor if you would like more information on this. Details can also be obtained from the NHS website and the Department of Health website: http://www.dh.gov.uk. You can also contact the UCLH Patient Advise Liaison Service (PALS) via phone: 020 3447 3042 or email: uclh.pals@nhs.net. In case you feel you would benefit from some emotional support, you can contact the Samaritans charity on 116 123, their helpline is open 24 hours per day. The CALM (Campaign against living miserably) charity also have a helpline open from 5pm to midnight every day on 0800 58 58 58 or a web chat (https://www.thecalmzone.net).

20. How will we use information about you?

Data Protection Information

We will need to use information from you and your medical records for this research project. This information will include your contact details [name, initials, telephone number(s), email address, address, NHS/hospital number], demographics [age, gender, marital status, ethnicity, education,

Dicken S, et al. BMJ Open 2024; 14:e079027. doi: 10.1136/bmjopen-2023-079027

occupation]), which will be held by UCL. People will use this information to do the research or to check your records to make sure that the research is being done properly. People who do not need to know who you are will not be able to see your name or contact details. Your data will have a code number instead. We will keep all information about you safe and secure. Once we have finished the study, we will keep some of the data so we can check the results. We will write our reports in a way that no-one can work out that you took part in the study.

UCL is the sponsor for this study based in the United Kingdom. The sponsor is the organisation responsible for ensuring that the study is carried out correctly. UCL will act as the data controller for this study. This means that they are responsible for looking after your information and using it properly. UCL will keep this information about you for 25 years after the study has finished. The UCL Data Protection Office provides oversight of UCL activities involving the processing of personal data and can be contacted at data-protection@ucl.ac.uk. Further information on how UCL uses your information can be found on our general research privacy notice here https://www.ucl.ac.uk/legal-services/privacy.

In order to conduct the study, staff outside your normal care will need access to your medical records. You will need to give permission to the study team to access your medical records, and for long-term storage in an anonymised form. All of your personal information will be kept strictly confidential. The study will legally comply with the Data Protection Act, 2018. Your patient records and study information will be stored behind a card-secure door, with access only by members of the research team. Any data that leaves the study sites (UCLH and UCL) will be anonymised. Your name and other identifiable information will not be recorded on records or samples. Your initials and trial identification number will be used on records or samples.

When you agree to take part in a research study, the information about your health and care may be provided to researchers running other research studies in this organisation and in other organisations. These organisations may be universities, NHS organisations or companies involved in health and care research in this country or abroad. Your information will only be used by organisations and researchers to conduct research in accordance with the UK Policy Framework for Health and Social Care Research.

We collect personal data directly from you, or your GP and/or hospital team if needed, for the purposes of carrying out this research study. We use your name, NHS number and contact details to contact you about the research study and make sure that relevant information about the study is recorded for your care, and to oversee the quality of the study. We only share your personal data in limited circumstances, with your GP, as set out in this information sheet, and with individuals from regulatory organisations where they need to look at your medical and research records to check the accuracy of the research study. The only people in UCL who will have access to information that identifies you will be people who need to contact you as part of the study or audit the data collection process. The people who analyse the study data will not be able to identify you and will not be able to find out your name, NHS number or contact details. We will keep all information about you safe and secure. Once we have finished the study, we will keep some of the data so we can check the results. The final study reports will contain anonymised data only and will not contain any identifiable data or personal information.

Dicken S, *et al. BMJ Open* 2024; 14:e079027. doi: 10.1136/bmjopen-2023-079027

Your personal data will be processed for the purposes outlined in this information sheet. The legal basis to process your personal data will be:

- to undertake the research study, on the basis of performance of a task carried out in the public interest and processing your special categories of personal data (e.g., health information) for research purposes.
- where required to make disclosures to your GP further to the results of any study test/questionnaire, on the basis of performance of a task carried out in the public interest, compliance with a legal obligation and processing your special categories of personal data (e.g., health information) for public health purposes.
- to contact you about participating in further research studies, only where you have provided consent on the consent form. You have the right to withdraw your consent to be contacted about future studies at any time, using the details set out at the end of this section.

To deliver the 8-week diets to you, the food suppliers will need your name, contact details and address. We will not share your personal details with the food suppliers. For one of the diets, we will control a secure, password-protected online food delivery account that only the research team can log into. We will ask you to add your name, phone number and address to the account delivery details for the purposes of delivering the diet to you. The supermarket will handle your data according to their privacy policy. We will ask you to add your details to an account for a second supermarket, to be used only if there are issues with delivery from the first supermarket. It is not possible to determine that you are involved in the study from this. When your time on this 8-week diet ends, we will delete your details from the account. For the other diet, you will need to give your name, address and contact details directly to the food supplier. We will give you the details needed to contact them, if you are eligible and decide to participate. You will need to agree to this on the consent form.

Your personal data will be processed only for so long as it is required for the research project. We will pseudonymise the personal data as soon as registered on the study database and endeavour to minimise the processing of personal data, wherever possible, to safeguard your privacy. In the event of any future loss of capacity to make decisions relating to your ongoing participation in this study, you will automatically be withdrawn from the study. The information already collected up to that point, may continue to be used confidentially in connection with this study and future research, if applicable.

In line with the regulations, at the end of the study your data will be securely archived for a minimum of 25 years.

Under data protection law, you have individual rights in relation to the personal data we hold about you. For the purposes of research where such individual rights would seriously impair research outcomes, such rights may be limited. In fact, as we need to manage your records in specific ways for the research to be reliable, we will not be able to let you see or change the data we hold about you. If you would like to exercise a right under data protection law or find out more about how we use your information, please contact data-protection@ucl.ac.uk.

Personal contact details will be removed at the end of the study if you decide to opt out of receiving a results summary.

Dicken S, *et al. BMJ Open* 2024; 14:e079027. doi: 10.1136/bmjopen-2023-079027

We may need to request information about you from your medical records via your GP if we are unable to obtain this from yourself. This information may include age, gender, ethnicity, job occupation, work pattern, educational level, marital status, medication intake, alcohol consumption, smoking habits, family history of obesity and possible related diseases. Any data sent from your GP to the researchers will be transferred using encrypted NHS email systems that are secure for sending confidential information.

21. What are your choices about how your information is used?

- You can stop taking part in the study at any time, without giving a reason, but we will keep information about you that we already have.
- We need to manage your records in specific ways for the research to be reliable. This means that we won't be able to let you see or change the data we hold about you.
- If you agree to take part in this study, you will have the option to take part in future research using your data saved from this study. This is explained in section 24.

22. Where can you find out more about how your information used?

You can find out more about how we use your information:

- at www.hra.nhs.uk/information-about-patients/
- our leaflet available from www.hra.nhs.uk/patientdataandresearch
- by asking one of the research team
- by sending an email to data-protection@ucl.ac.uk, or
- by ringing us on 0203 108 8764

If you remain unsatisfied, you may wish to contact the Information Commissioner's Office (ICO) (https://ico.org.uk/concerns/handling/).

23. Will my GP be informed of my involvement?

With your permission, your GP will be notified that you are taking part in this study. If the study investigators became concerned about your well-being or about the implications of what you tell us for someone else's well-being we would need to inform your GP or other professionals. We would, of course, discuss this with you first. In addition, should your blood test results show any abnormality, we will let your GP know, who will be able to make necessary arrangements for your best care. You will be asked to agree to this when signing the consent form.

24. What will happen to my blood samples?

As part of this trial, we will collect new blood samples from you during the face-to-face visits, as detailed above. These blood samples will be stored and analysed at UCLH and the Centre for Obesity Research, University College London (UCL). Samples will be anonymised, which means they will not be identifiable. The samples will be identified only by your study number and will not be directly marked with your name. The samples will only be used for the study described above, however, with your consent, any remaining blood samples you provided for the study may be

Dicken S, *et al. BMJ Open* 2024; 14:e079027. doi: 10.1136/bmjopen-2023-079027

retained after the end of the study for future research. Only researchers involved in this study will have access to the blood samples. You can choose how you would like your samples to be used after the study ends in the consent form. We can transfer your samples to the Obesity Research Biobank Syndicate (ORBiS) run by COR, where they will be stored in a secure database and used for further research, or they can be safely disposed of. Your samples will be disposed of in accordance with the Human Tissue Act 2004. If you choose to withdraw from the study, any stored blood or tissue samples that can still be identified as yours will be destroyed if you wish.

25. What will happen to the results of the trial?

The results of the study will be available after it finishes and will usually be published in a medical journal or presented at a scientific conference. The data will be anonymised and none of the participants involved in this study will be identified in any report or publication. We will be happy to make the results of the study available to you. You can request a lay summary of the study results by ticking the corresponding box in the consent form.

26. Who is conducting the trial?

The study is being run by the UCL Centre for Obesity Research who care for patients living with obesity.

27. Who is organising and funding the trial?

This research study is being organised by the Centre for Obesity Research, University College London (UCL). The Chief Investigator is Professor Rachel L. Batterham (Consultant Obesity Physician, Diabetologist, and Endocrinologist) who has vast experience in clinical research studies. This study is being sponsored by University College London (UCL) and being funded by the National Institute for Health Research (NIHR) and Rosetrees trust.

28. How have patients and the public been involved in this trial?

In designing and planning this study, we have taken into account the opinions of UCLH staff following a focus group discussion, who have reviewed and given feedback on the study design and this Information Sheet.

29. Who has reviewed the trial?

It is important that when we carry out research, we make sure the study design is as good as possible to reduce the risk of bias and poor-quality research. All research in the NHS is looked at by independent group of people, called a Research Ethics Committee, to protect your interests. This study has been reviewed and given favourable opinion by the Yorkshire & The Humber - Sheffield Research Ethics Committee. Academics from UCL, National Institutes of Health in the US and academic peer-reviewers have also evaluated and reviewed the study design.

30. Further information and contact details

Dicken S, *et al. BMJ Open* 2024; 14:e079027. doi: 10.1136/bmjopen-2023-079027

If you require any further information or have any concerns while taking part in the study, please contact one of the following people:

Samuel Dicken Phone: 07415690476 Email: samuel.dicken.20@ucl.ac.uk

Fred Jassil Email: friedrich.jassil.13@ucl.ac.uk

If you decide you would like to take part, then please read and sign the consent form. You will be given a copy of this information sheet and the consent form to keep. A copy of the consent form will be filed in your patient notes, one will be filed with the study records and one may be sent to the Research Sponsor.

You can have more time to think this over if you are at all unsure.

Thank you for taking the time to read this information sheet and for considering this study.

Dicken S, *et al. BMJ Open* 2024; 14:e079027. doi: 10.1136/bmjopen-2023-079027
