# Supplementary material for: UPDATE trial: investigating the effects of ultra-processed versus minimally processed diets following UK dietary guidance on health outcomes: a protocol for an 8-week community-based cross-over randomised controlled trial in people with overweight or obesity, followed by a 6-month behavioural intervention
Source: BMJ Open. 2024 Mar 11;14(3):e079027. doi: 10.1136/bmjopen-2023-079027 (PMC10936475; doi:10.1136/bmjopen-2023-079027)
Supplement: Supplementary data [file bmjopen-2023-079027supp002.pdf]

CONSENT FORM

Centre for Obesity Research
Division of Medicine
University College London
Rayne Building
5 University Street
London WC1E 6JF
Telephone: 020 7679 0788

Email: samuel.dicken.20@ucl.ac.uk
r.batterham@ucl.ac.uk

UPDATE trial: A study comparing the health effects of two diets following UK dietary guidance in people living with overweight or obesity.

Name of Chief Investigator: Professor Rachel L Batterham **Participant screening number:** [ ]

Sponsor Protocol Number: 151582
IRAS ID number: 311525

Thank you for considering taking part in this project. If you have any questions arising from the information sheet or explanation already given to you, please ask the research team before you decide whether to join, contact details are on the top right-hand side of this page. This consent form is to confirm that you are happy to undergo the screening assessments to participate in the study, and if eligible, to participate in the study. For some elements, you must circle an option (e.g., consent/do not consent). You must circle one option and initial the box to confirm your selection. Leaving any boxes not initialled will result in the consent form being incomplete and not valid.

Please initial box

1	I confirm that I have read the participant information sheet (Version 1.1, dated 12.12.2022), which explains the benefits and risks of taking part in the study.	
2	I confirm that I have been provided with sufficient information and opportunity to ask any questions about the study and have had these answered satisfactorily.	
3	I confirm that I have been informed about any risk that may result from taking part in this study and that I have had sufficient time to consider whether or not I want to take part.	
4	I confirm that I understand the requirements for participating in the study, and that I am able to attend all relevant study sessions and procedures as outlined in the participant information sheet (Version 1.1, dated 12.12.2022).	
5	I understand that my participation in this study is voluntary. It is my right to withdraw from the study at any time, with no penalties. I do not need to give a reason for doing so. This will not affect my standard care or my legal rights.	
6	I understand that relevant sections of my medical notes and data collected during the study may be looked at by individuals from the sponsor of the trial (University College	

Dicken S, et al. BMJ Open 2024; 14:e079027. doi: 10.1136/bmjopen-2023-079027

	London) and responsible persons authorised by the sponsor, from regulatory authorities or from the NHS Trust, where it is relevant to my taking part in this research. I give permission for these individuals to have access to my records. I understand that such information will be handled appropriately and confidentially.	
7	I understand that the UCL research team will access parts of my medical record and my data collected in the study. I give permission for this access.	
8	I understand that my name, contact details and address need to be used for the purposes of delivering the diets to my home at a time that is convenient for me, as outlined in the participant information sheet (Version 1.1, dated 12.12.2022). The UCL research team will not share my details with the food suppliers. I agree that I will provide my contact details and address to the food suppliers to deliver the diets.	
9	I give permission for a researcher to contact me for the purposes of offering support during the interventions, ensuring the diet deliveries are received, in collecting study samples and to remind me to complete the study measurements.	
10	I give permission for my anonymised data to be used in future publications related to this study and to be stored securely for up to 25 years after the end of the study.	
11	I acknowledge that UCL will handle my personal data for the purposes of this research study and I have been provided with information about how UCL handles personal data, as described in the participant information sheet (Version 1.1, dated 12.12.2022).	
12	I understand that my GP will be informed that I am participating in this study.	
13	I consent that in the event that I lose the capacity to make decisions relating to my ongoing participation in this study, I will automatically be withdrawn from the study. The information already collected up to that point may continue to be used confidentially in connection with this study and future research, if applicable, as described in the participant information sheet (Version 1.1, dated 12.12.2022).	
14	I agree to the one-to-one interview at the 6-month follow-up to be recorded for data analysis purposes, and I understand that some of the things I say may be quoted verbatim in publications without me being directly identified.	
15	I consent / do not consent (please circle) to being potentially chosen to have the MRI brain scans as described in the participant information sheet (Version 1.1, dated 12.12.2022).	
16	I give permission for my blood samples to be stored and analysed at the University sites.	
17	At the end of the study, I consent / do not consent (please circle) to having my blood samples stored in ORBiS for future research as explained in the participant information sheet (Version 1.1, dated 12.12.2022). If I do not agree, I understand that my samples will be disposed of according to Human Tissue Act 2004.	
18	I consent / do not consent (please circle) for my personal information to be retained and used to contact me for the purpose of participating in future research.	

19	I would / would not (please circle) like to receive a summary of the results.	
20	I voluntarily consent and agree to undergoing a screening assessment to determine my eligibility for the study, and if eligible, participating in the above study.	

---------------------------------- ----------------------------- ----------------------
Name of participant Signature Date

I confirm that I have explained the study to the above participant and have answered questions honestly and fully.

---------------------------------- ---------------------------- ----------------------
Name of investigator taking consent Signature Date

Comments or concerns during the study
If you have any comments or concerns, you may discuss these with the investigators. If you wish to go further and complain about any aspect of the way you have been approached or treated during the course of the study, you should write or get in touch with the Complaints Manager, UCL Hospitals. Please quote the study number on the front page of this consent form.

When completed: 1 (original) for participant; 1 (original) for researcher site file; 1 to be kept in medical notes.

Dicken S, *et al. BMJ Open* 2024; 14:e079027. doi: 10.1136/bmjopen-2023-079027
