# Supplementary material for: UPDATE trial: investigating the effects of ultra-processed versus minimally processed diets following UK dietary guidance on health outcomes: a protocol for an 8-week community-based cross-over randomised controlled trial in people with overweight or obesity, followed by a 6-month behavioural intervention
Source: BMJ Open. 2024 Mar 11;14(3):e079027. doi: 10.1136/bmjopen-2023-079027 (PMC10936475; doi:10.1136/bmjopen-2023-079027)
Supplement: Supplementary data [file bmjopen-2023-079027supp003.pdf]

Supplementary Materials: Contraception list

Female participants

Females of childbearing potential are eligible to participate if they agree to use a highly effective contraception method for the duration of the trial and until 6 weeks after treatment discontinuation. Women are considered of childbearing potential following menarche and until becoming post-menopausal unless permanently sterile. Women are considered permanently sterile if they have had documented hysterectomy, bilateral salpingectomy or bilateral oophorectomy. Postmenopausal state is defined as no menses for 12 months without any medical cause. A high Follicle Stimulating Hormone (FSH) level in the postmenopausal range may be used to confirm a postmenopausal state in women not using hormonal contraception or Hormonal Replacement Therapy (HRT). However in the absence of 12 months of amenorrhoea, a single FSH measurement is insufficient.

Highly effective contraceptive methods include:

- Combine (oestrogen and progesterone containing) hormonal contraception associated with inhibition of ovulation:
  - Oral
  - Intravaginal
  - Transdermal
- Progesterone-only hormonal contraception associated with inhibition of ovulation:
  - Oral
  - Injectable
  - Implantable
- Intrauterine device.
- Intrauterine hormone-releasing system.
- Bilateral tubal occlusion.
- Vasectomised partner.
- True sexual abstinence (refraining from sexual intercourse – only acceptable when this is in line with the preferred and usual lifestyle of the subject).

Male participants

Male participants with partners of childbearing potential should use barrier methods of contraception for the duration of the trial until 6 weeks after treatment discontinuation.
