# Supplementary material for: UPDATE trial: investigating the effects of ultra-processed versus minimally processed diets following UK dietary guidance on health outcomes: a protocol for an 8-week community-based cross-over randomised controlled trial in people with overweight or obesity, followed by a 6-month behavioural intervention
Source: BMJ Open. 2024 Mar 11;14(3):e079027. doi: 10.1136/bmjopen-2023-079027 (PMC10936475; doi:10.1136/bmjopen-2023-079027)
Supplement: Supplementary data [file bmjopen-2023-079027supp004.pdf]

UPDATE Exit Interview Guide

Full details of the intervention development and content will be provided in a future publication focussed on the behavioural support intervention. This document outlines the support programme exit interview. This guide is to ensure that key aspects of participants' opinions of participating in the trial are covered during the interview. This is a semi-structured interview guide, and as such, a respondent-sensitive approach should be taken, allowing deviation from question order and raising additional issues if appropriate, depending on the conversation with each individual.

Introduction:

Introduce yourself and explain that the purpose of the call is to find out more about how they have found participating in the behavioural support programme. Ask participants to be as open and honest as possible in their answers and reassure that there are no right or wrong answers – we're just interested in their opinions. Ask permission to record the phone call so that we can come back to their answers.

When you first saw the advert for the UPDATE trial, what made you want to take part?
Was the offer of behavioural support appealing to you? *[Prompt on reasons/what they hoped to get out of the behavioural support]*

I will ask about individual parts of the programme in more detail later, but overall, did you feel you got any benefit from the behavioural support? [*Prompt on what this was/reasons why/why not]*

When you first entered the trial, you weren't told it was about ultra-processed food (UPF) / minimally processed food (MPF). How did you feel when you found this out? [*Prompt on reasons/whether this something they felt positively or negatively about]*

Were you familiar with the ideas of UPF or MPF before you started this programme? Do you feel you understand these more as a result of taking part?

[If they were aware of UPF/MPF before the trial] How worthwhile did you think it was to reduce UPF/increase MPF before you started this programme? *[Probe on reasons for it being worthwhile versus not]*

How worthwhile to do you feel it is to reduce UPF/increase MPF now? *[Probe on reasons]*

I am now going to ask about a different parts of the behavioural support programme. I want to know what worked for you, but also really want to know what didn't work. If there was anything you didn't use it's also really helpful for us to know.

[Note to researcher – these sections do not have to be in order. If it feels more natural to start talking about another part of the programme, i.e. because a participant raises it, then be led by them]

Let's talk about:

Dicken S, *et al. BMJ Open* 2024; 14:e079027. doi: 10.1136/bmjopen-2023-079027

The **behavioural support calls/sessions** [*Prompt on how many they received and if they did not receive all, ask why they missed some. Researcher should be aware in advance of which calls/sessions they received and can use this to help remind them if needed].*

- Ask if they did it online/in person and reasons for preference. [Use 'sessions' rather than calls if they were in person].
- What were your feelings about the calls/sessions overall? Were they helpful?
- Let's break it down and think about the first one. That was the **introductory call/session** where you were asked about your experience of diet A/B, what influenced how you ate before joining the trial, personal barriers, and were asked to fill in a food/mood diary. Did you find this call useful? *[Probe on what they liked/didn't like, the length/the areas covered/the diary/what could have been done differently]*
- Let's think about the call you had a week later (the **first behavioural support call/session**). Did you find this call useful? *[Probe on what they liked/didn't like, the length/the areas covered/the diary/what could have been done differently]*
- In the month three call physical activity was introduced. How did you feel about this? *[Probe on what they liked/didn't like, the length/the areas covered/the diary/what could have been done differently]*
- What about the other (check-in) calls in-between these? Were they useful? [*Prompt on reasons]*
- Overall, how did you feel about the frequency of calls/sessions – was it too little/too much/about right for you? *[Prompt on reasons and what they might have preferred where relevant]*
- Still thinking about the calls/sessions - do you have any suggestions for what we could do differently in future?

In between the calls you got **weekly emails**. How did you feel about these? Did you read them? Were they helpful? Was weekly OK for you? What about the content of the emails? Was this a good way to communicate with you between calls/sessions? *[Probe on other preferences if not].*

Did you use the **UPDATE behavioural support** (green) booklet alongside your behavioural support calls?

- If no, explore why not.
- If yes:
  - What were your feelings about it overall? Was it helpful to use during the call?
  - How did you feel about the design/content?
  - Did you refer to it in between the calls? *(Probe on why/why not)*
  - Do you still refer to it now?
  - Did your interest in or use of the booklet change over the course of the programme? *[Probe on how/why]*
  - Do you think the booklet influenced your UPF/MPF intake? *(Probe on why/why not)*
  - Do you think it influenced your physical activity? *(Probe on why/why not)*
  - Is there anything you would add or change in the green booklet?

Did you use the **UPDATE tracker** (blue) booklet?

- If no, explore why not.
- If yes:
  - What were your feelings about it overall?
  - How did you feel about the design/content?
  - Did you use it more than once?
  - Did your interest in or use of it change over the course of the programme? *[Probe on how/why]*
  - Do you think it influenced your UPF/MPF intake? *(Probe on why/why not)*
  - Do you think it influenced your physical activity? *(Probe on why/why not)*
  - How did you feel about the idea of tracking and setting goals?
  - As we talked about during the programme tracking and setting goals is important. Do you think this was the best way to do it? Do you have ideas of different ways that might work in this type of programme?

Did you use the **UPDATE website**?

- If no, explore why not.
- If yes:
  - What were your feelings about it overall? Was it helpful?
  - How did you feel about the design/content?
  - Did you use it more than once? *[Prompt on how often if so]*
  - What prompted you to use it?
  - Did you use the food mapper/recipes/other resources [*Probe on what they liked and didn't like/what could be improved on each]*
  - Did your interest in or use of the website change over the course of the programme? *[Probe on how/why]*
  - Do you think anything on the website influenced your UPF/MPF intake? (Probe on why/why not)
  - Do you think anything on the website influenced your physical activity?
  - Is there anything you would add or change?

Did you attend any **Group Sessions**?

- If no, explore why not.
- If yes:
  - How many did you attend?
  - What were your feelings about them overall? Were they helpful? Any sessions that stood out as especially helpful/relevant to you? *[Probe as needed]*
  - How did you feel about the format/length?
  - Is there anything you would add or change?

Summary

- Overall, would you recommend the behavioural support programme to other people? *[Probe on reasons why/why not]*

- You were offered the behavioural support programme as part of the larger study. Would you have taken part if only the behavioural support programme had been offered?
- Do you think having done the first part of the trial had any impact on how you felt about the behavioural support programme? *(Probe on what/why)*
- Have you felt anything has changed for you as a result of taking part, positively or negatively? *(Prompt: noticed any physical/psychological changes, changes in thoughts/feelings, wellbeing?)*
- Did your feelings about the behavioural support programme change from when you first started it to now? *(Probe on how/why)*
- Is there anything you learned during the programme that you are still doing now?
- Is there anything that was missing for you that you haven't told us about?
- Thinking about the study as a whole (both parts) – how did you feel about the assessments *(probe on number, type)* – is there anything else you'd like to say about the way the study was designed or set up?
- Is there anything else you would like to say, that we've not already spoken about?

Thank participant for their time and participation in the interview, and the study as a whole.

Dicken S, *et al. BMJ Open* 2024; 14:e079027. doi: 10.1136/bmjopen-2023-079027
