# Supplementary material for: UPDATE trial: investigating the effects of ultra-processed versus minimally processed diets following UK dietary guidance on health outcomes: a protocol for an 8-week community-based cross-over randomised controlled trial in people with overweight or obesity, followed by a 6-month behavioural intervention
Source: BMJ Open. 2024 Mar 11;14(3):e079027. doi: 10.1136/bmjopen-2023-079027 (PMC10936475; doi:10.1136/bmjopen-2023-079027)
Supplement: Supplementary data [file bmjopen-2023-079027supp005.pdf]

Supplementary Materials: Schedule of Assessments

			First diet intervention				Second diet intervention		Behavioural support program	
	**Screening**	**Baseline Assessment first diet**	**4-week follow-up first diet**	**8-week follow-up first diet**	**4-week washout period**	**Baseline Assessment second diet**	**4 -week follow-up second diet**	**8-week follow-up second diet**	**Behavioural support counselling**	**6-month follow-up**
**Visit No:**	**1**	**2**	**3**	**4**		**5**	**6**	**7**	**8**	**9**
**Week**	**0**	**1-2**	**6**	**10**	**11-14**	**15-16**	**20**	**24**	**24**	**49**
Informed Consent	✓[a]									
Concomitant Medication review	✓	✓	✓	✓		✓	✓	✓		✓
Adverse Events review		✓	✓	✓		✓	✓	✓		✓
Urine Pregnancy Test	✓									
Socio-demographics	✓									
Medical History and Co-morbidities	✓	✓	✓	✓		✓	✓	✓		✓
Vital Signs	✓	✓	✓	✓		✓	✓	✓		✓
Randomisation[b] for diet order		✓								
Height	✓									
Weight	✓	✓	✓	✓		✓	✓	✓		✓
Waist circumference		✓	✓	✓		✓	✓	✓		✓
BIA		✓	✓	✓		✓	✓	✓		✓
Blood sample collection		✓		✓		✓		✓		✓
Meal test		✓		✓		✓		✓		✓
Accelerometry (ActiGraph)		✓		✓		✓		✓		✓
Physical activity and sleep questionnaires		✓	✓	✓		✓	✓	✓		✓
Physical Function Assessment (6MWT, STS, HGST)		✓	✓	✓		✓	✓	✓		✓
Mental health and wellbeing Questionnaires		✓	✓	✓		✓	✓	✓		✓
Eating behaviour Questionnaires		✓	✓	✓		✓	✓	✓		✓
COM-B questionnaire		✓								✓
Behavioural support program									✓	
One to one semi-structured phone/video call interview										✓

Supplementary Materials: Schedule of Assessments

Diet assessment: 24-hr recall (Intake24)	✓	✓	✓	✓		✓	✓	✓		✓
Diet assessment: FFQ (EPIC-Norfolk FFQ)		✓	✓	✓		✓	✓	✓		✓
Diet assessment: IBDA			✓	✓			✓	✓		
MRI brain scans in a subset		✓		✓		✓		✓		

[a] Prior to Screening.

[b] Randomisation will be undertaken after the participant has completed the baseline diet assessment.

Abbreviations: BIA: Bioelectrical Impedance Analysis; COM-B: capability, opportunity, motivation – behaviour; FFQ: food frequency questionnaire; HGST: handgrip strength test; IBDA: image-based dietary assessment; MRI: magnetic resonance imaging; STS-test: sit-to-stand test; 6MWT: 6-minute walk test.
