# Supplementary material for: UPDATE trial: investigating the effects of ultra-processed versus minimally processed diets following UK dietary guidance on health outcomes: a protocol for an 8-week community-based cross-over randomised controlled trial in people with overweight or obesity, followed by a 6-month behavioural intervention
Source: BMJ Open. 2024 Mar 11;14(3):e079027. doi: 10.1136/bmjopen-2023-079027 (PMC10936475; doi:10.1136/bmjopen-2023-079027)
Supplement: Author's manuscript [file bmjopen-2023-079027.draft_revisions.pdf]

BMJ Open is committed to open peer review. As part of this commitment we make the peer review history of every article we publish publicly available.

When an article is published we post the peer reviewers' comments and the authors' responses online. We also post the versions of the paper that were used during peer review. These are the versions that the peer review comments apply to.

The versions of the paper that follow are the versions that were submitted during the peer review process. They are not the versions of record or the final published versions. They should not be cited or distributed as the published version of this manuscript.

BMJ Open is an open access journal and the full, final, typeset and author-corrected version of record of the manuscript is available on our site with no access controls, subscription charges or pay-per-view fees (http://bmjopen.bmj.com).

If you have any questions on BMJ Open's open peer review process please email info.bmjopen@bmj.com

BMJ Open

UPDATE trial: Investigating the effects of Ultra-Processed versus minimally processed Diets following UK dietAry guidance on healTh outcomEs: a protocol for an 8-week community-based crossover randomised controlled trial in people with overweight or obesity, followed by a 6-month behavioural intervention.

Journal:	*BMJ Open*
Manuscript ID	bmjopen-2023-079027
Article Type:	Protocol
Date Submitted by the Author:	18-Aug-2023

Complete List of Authors:	Dicken, Samuel; UCL, Centre for Obesity Research<br>Makaronidis, Janine; University College London, Centre for Obesity Research, Department of Medicine; University College London Hospital (UCLH), Bariatric Centre for Weight Management and Metabolic Surgery<br>van Tulleken, Chris; University College London, Division of Infection<br>Jassil, Friedrich; University College London, Division of Medicine; University College London Hospital (UCLH), Bariatric Centre for Weight Management and Metabolic Surgery<br>Hall, Kevin; National Institute of Diabetes and Digestive and Kidney Diseases, Bethesda, MD, National Institutes of Health<br>Brown, Adrian; UCL, Centre for Obesity Research, Department of Medicine; University College London Hospital (UCLH), Bariatric Centre for Weight Management and Metabolic Surgery<br>Gandini Wheeler-kingshott, Claudia; Queen Square Multiple Sclerosis Centre, UCL Institute of Neurology, Neuroinflammation; Brain MRI 3T Research Center, C. Mondino National Neurological Institute<br>Fisher, Abigail; University College London, Department of Behavioural Science and Health<br>Batterham, Rachel; UCL, Centre for Obesity Research, Department of Medicine; University College London Hospital (UCLH), Bariatric Centre for Weight Management and Metabolic Surgery

Keywords:	NUTRITION & DIETETICS, Obesity, PUBLIC HEALTH, Clinical Trial

SCHOLARONE™ Manuscripts

UPDATE trial: Investigating the effects of **U**ltra-**P**rocessed versus minimally processed **D**iets following UK diet**A**ry guidance on heal**T**h outcom**E**s: a protocol for an 8-week community-based crossover randomised controlled trial in people with overweight or obesity, followed by a 6-month behavioural intervention.

Samuel J. Dicken[1], Janine Makaronidis[1,2,3], Chris Van Tulleken[4], Friedrich C Jassil[1,2,3], Kevin Hall[5], Adrian Brown[1,2,3], Claudia A. M. Gandini Wheeler-Kingshott[6,7,8], Abi Fisher[9], Rachel L. Batterham[1,2,3]

1. Centre for Obesity Research, Department of Medicine, University College London (UCL), London WC1E 6JF, UK.
2. Bariatric Centre for Weight Management and Metabolic Surgery, University College London Hospital (UCLH), London NW1 2BU, UK.
3. National Institute for Health Research, Biomedical Research Centre, University College London Hospital (UCLH), London W1T 7DN, UK.
4. Division of Infection, University College London, London WC1E 6BT, UK.
5. National Institute of Diabetes and Digestive and Kidney Diseases, National Institutes of Health, US.
6. NMR Research Unit, Queen Square MS Centre, Department of Neuroinflammation, UCL Institute of Neurology, Faculty of Brain Sciences, University College London, London, United Kingdom.
7. Department of Brain and Behavioural Sciences, University of Pavia, Pavia, Italy.
8. Digital Neuroscience Center, IRCCS Mondino Foundation, Pavia, Italy.
9. Behavioural Science and Health, University College London, London, UK.

For peer review only

Abstract

Introduction Obesity increases the risk of morbidity and mortality. A major driver has been the increased availability of ultra-processed food (UPF), now the main energy source in UK diets. The UK Eatwell Guide (EWG) provides public guidance for a healthy balanced diet, but offers no UPF guidance. Whether a healthy balanced diet can largely consist of UPFs is unclear. No studies have assessed whether the health impact of dietary guidelines depends on the extent and purpose of processing, nor provided behavioural support to UK adults with overweight/obesity to reduce their UPF intake.

Methods and analysis UPDATE is a 2x2 crossover randomised controlled trial followed by a 6-month behavioural intervention. Fifty-five adults aged ≥18, living with overweight/obesity (≥25 to <40 kg/m^2), with ≥50% of habitual energy intake from UPFs will receive an 8-week UPF diet and an 8-week minimally processed food (MPF) diet, both following EWG recommendations, in a random order, with a 4-week washout period. All food and drink will be provided. Participants will then receive 6-months of behavioural support to reduce UPF intake. The primary outcome is the difference in weight change between UPF and MPF diets. Secondary outcomes include changes in diet, waist circumference, body composition, heart rate, blood pressure, cardiometabolic risk factors, appetite regulation, sleep quality, physical activity levels, physical function and strength, wellbeing and aspects of behaviour change and eating behaviour at 8 weeks between UPF and MPF diets, and at 6-months follow-up. Quantitative assessment of changes in brain magnetic resonance imaging functional resting-state connectivity between MPF and UPF diets, and qualitative analysis of the behavioural support intervention for feasibility and acceptability will also be undertaken.

Ethics and dissemination The Sheffield Research Ethics Committee approved the trial (22/YH/0281). Results will be reported in peer-reviewed journals, conferences, PhD thesis and lay media.

Trial registration number NCT05627570.

Strengths and limitations of this study

- UPDATE will be the longest and first free-living diet trial to date to investigate the impact of UPF on weight and other health parameters, and the first trial to provide behavioural support to UK adults living with overweight/obesity to reduce their UPF intake.
- Our findings will expand the knowledge regarding the health consequences of UPFs, to inform policy and the development of UPF regulations.
- UPDATE is directly relevant for UK public health food policy as the interventions are based on the UK Eatwell Guide, as well as worldwide, given the similarities between UK and other national dietary guidelines.
- Limitations include the exclusion of individuals with type 2 diabetes or with dietary restrictions (e.g., vegan, vegetarian, Halal, Kosher).

For peer review only

Introduction

Obesity is a global healthcare challenge. Nearly two thirds of UK adults now live with overweight or obesity[1], increasing the risk of life-limiting disease and premature mortality[2]. The overall annual cost of obesity in the UK is estimated at £58 billion[3]. Of which, the National Health Service (NHS) spends over £6 billion/year on obesity-related health issues, which is expected to rise to nearly £10 billion/year by 2050[4].

A major driver of the obesity epidemic has been the shift in the food environment with increased availability of ultra-processed food (UPF)[5–7]. Defined by NOVA, UPFs are industrial formulations with five or more ingredients, using extracts of original foods with preservatives, flavourings and colours[8]. UPFs include breakfast cereals, sweets, packaged breads and ready meals. Over 50% of UK energy intake now comes from UPFs, displacing more traditional minimally processed food (MPF)[9]. In prospective cohort studies, higher UPF intakes are associated with increased risks of weight gain, overweight/obesity[10,11], cardiometabolic disease[12], gastrointestinal disorders, cancer[13], mental health problems[14], lower physical strength[15] and all-cause mortality[16].

The Eatwell Guide (EWG) provides the UK public with guidance for a healthy, balanced diet[4,17,18]**.** The EWG focusses on macronutrients and food groups, such as choosing foods lower in saturated fat, sugar and salt, and eating five daily portions of fruit and vegetables[18]. The EWG recommends 'reduced fat' or 'lower sugar' reformulated foods, based on European Food Safety Authority nutrition claim regulations. However, this promotes the intake of UPFs[19]. Given their improved nutritional quality from reduced saturated fat, added sugar and/or salt, reformulated UPFs are often marketed as "healthy"[20]. However, the associations between high UPF intakes and adverse health outcomes appear to independent of diet quality or diet pattern[16].

In the only randomised trial to date examining the effect of energy-matched UPF versus MPF diets, participants gained nearly 1kg body weight on the 2-week UPF diet, but lost nearly 1kg on the 2-week MPF diet, with over 500kcal/day differences in energy intake[21]. The diets were also matched for presented energy density, carbohydrate, sugar, fat, sodium and fibre content. The MPF diet also led to favourable changes in appetite-regulating gut hormones compared with the UPF diet. This further suggests that the adverse effects of high UPF intakes are independent of individual nutrients such as fat, salt and sugar. In epidemiological substitution modelling, replacing UPFs for MPFs leads to weight loss, whereas replacing MPFs for UPFs leads to weight gain, independent of diet pattern (by adjusting for adherence to a Mediterranean diet)[22]. However, to date, UK organisations such as the British Nutrition Foundation and the Scientific Advisory Committee on Nutrition (SACN) do not recommend the inclusion of UPF in dietary guidelines. This is due to the largely observational body of evidence regarding the negative impact of ultra-processing on health, and lack of high-quality interventional evidence[23,24].

Adopting a healthy diet still presents a significant behaviour change and lifestyle modification which can be challenging for many individuals. The prevalence of obesity in the UK are also present amongst UK healthcare professionals (HCPs)[1,25]. Many HCPs consume an unhealthy diet[26], attributed to the high availability and accessibility of ready-to-eat unhealthy food and

For peer review only

lack of healthier choices in the workplace especially during night-shifts, stress-driven eating, long working hours, and staff shortages limiting the ability to take breaks[27]. It is therefore important to support individuals to reduce their UPF intake, and in the process, identifying barriers and facilitators to develop effective strategies for long-term behaviour change.

Objectives and hypotheses

To date, no studies have assessed whether the health impact of following dietary guidelines is dependent on food processing, nor whether providing behavioural support to UK adults living with overweight/obesity can help to reduce their UPF intake.

Therefore, this study aims to compare the health effects between MPF and UPF diets that follow the EWG recommendations, and the feasibility and acceptability of a behavioural support programme to reduce UPF intake and be more physically active.

The primary objective is to compare weight change between MPF and UPF diets following EWG recommendations (Table 1). Secondary objectives are to compare changes in cardiometabolic, behavioural, mental and hormonal outcomes between MPF and UPF diets following EWG recommendations and explore the feasibility and acceptability of the behavioural support intervention.

We hypothesise that there will be a difference in the change in weight and other health measures between the two diets; whereby consuming an *ad libitum* MPF diet complying with the EWG will result in weight loss and favourable changes in cardiometabolic, behavioural, mental and hormonal outcomes, whereas consuming an *ad libitum* UPF diet complying with the EWG will result in no change in weight or cardiometabolic, behavioural, mental and hormonal outcomes.

Table 1. UPDATE trial design based on PICOT

Population	Adults (UCLH staff) living with overweight or obesity, and with ≥50% of habitual energy intake from UPFs.
Intervention	A minimally processed diet complying with UK EWG guidance.
Comparator	An ultra-processed diet complying with UK EWG guidance.
Outcome (primary)	Mean difference in percent weight change between intervention and comparator at 8 weeks.
Timepoints	Primary outcome assessed from baseline to 8 weeks follow-up on each diet.

EWG, Eatwell Guide; UCLH, University College London Hospitals; UK, United Kingdom; UPF, ultra-processed food.

Methods and analysis

Trial design and study setting

UPDATE is a single-site, community-based, 2x2 crossover randomised controlled trial (RCT) followed by a six-month behavioural intervention, conducted in the UK by the Centre for Obesity Research, Division of Medicine, University College London (UCL) and University College London Hospitals (UCLH). The protocol was designed according to Standard Protocol

For peer review only

Items: Recommendations for Interventional Trials (SPIRIT) guidelines (https://www.spirit-statement.org)[28].

In summary, 55 adults aged 18 or older, living with overweight/obesity (between 25 kg/m^2 and 40 kg/m^2), with 50% or more of habitual energy intake from UPFs will be recruited. Participants will receive an 8-week, minimum 80% UPF diet and an 8-week, minimum 80% MPF diet, both following UK EWG macronutrient recommendations, in a random order, with a 4-week washout period. All food and drink will be provided. All participants will then receive a 6-month behavioural support programme to reduce UPF (Figure 1).

The trial is funded by the National Institute for Health and Care Research UCLH Biomedical Research Council and Rosetrees Trust, and UCL/UCLH are the Sponsors. The Sponsor and funders were not involved in the design and conduct of the trial.

For peer review only

Figure 1. Flow chart of UPDATE trial.

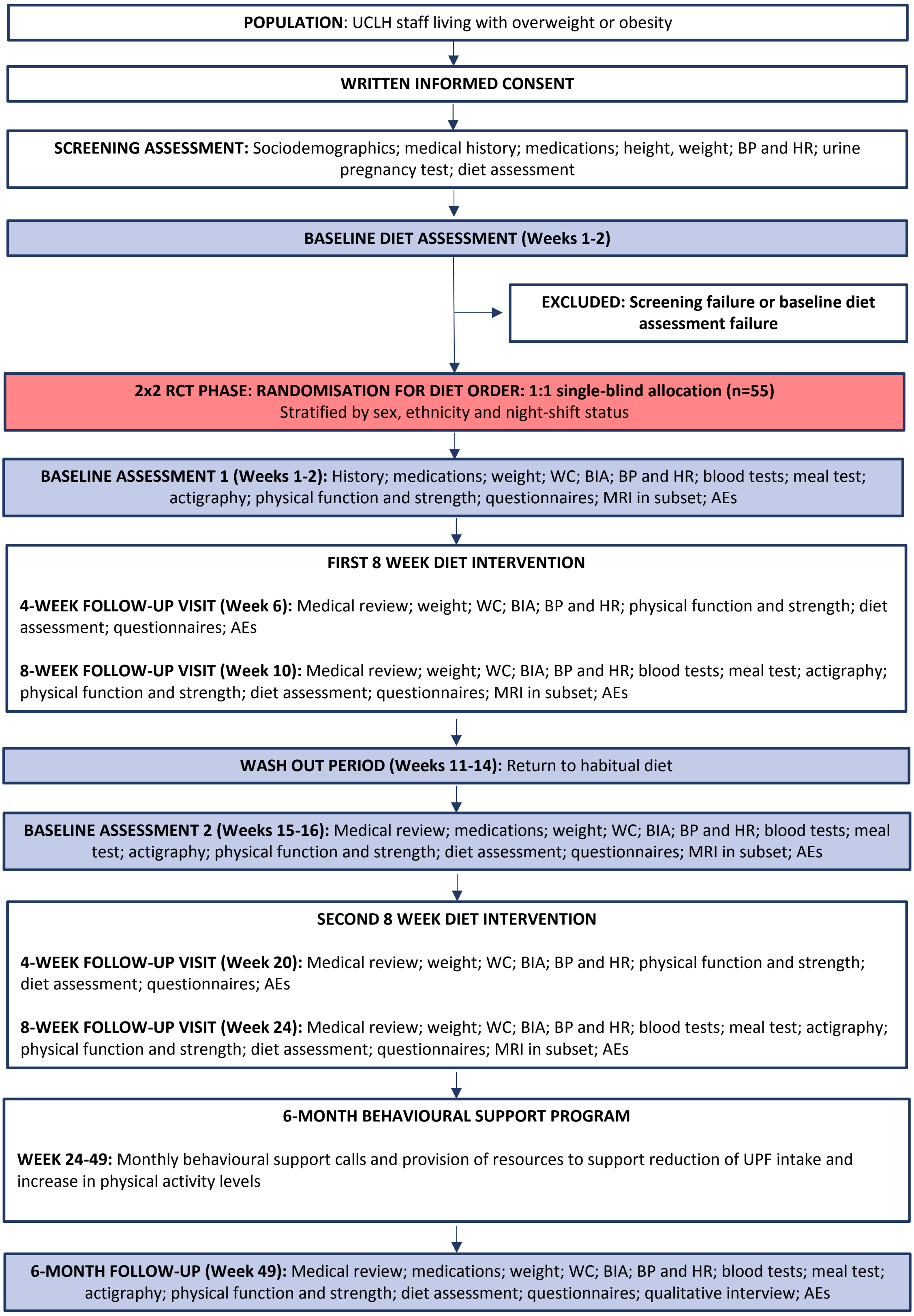

AE, adverse events; BIA, bio-impedance analysis; BP, blood pressure; HR, heart rate; MRI, magnetic resonance imaging; RCT, randomised controlled trial; UCLH, University College London Hospitals; WC, waist circumference.

Methods: Participants, interventions, and outcomes

Recruitment

Potential participants will be identified through advertising at UCLH/UCL (e.g. websites, Trust email, posters, internal communications) and on social media (Twitter).

Interested individuals will receive a Participant information sheet (PIS) (Supplementary Material 1), and be offered a phone call with the research team. Researchers will then explain the screening procedure and the aims, methods, anticipated benefits and potential hazards of the trial, and invite individuals to attend an in-person visit to undergo screening. Written informed consent will be sought a minimum of 24 hours after receiving the PIS, at the in-person visit (see Supplementary Material 2 for the approved consent form). Participants can withdraw at any time, without giving a reason.

Eligibility criteria

Written informed consent will be obtained prior to commencing screening based on the inclusion and exclusion criteria, and before any research-associated measurement is taken (Table 2).

Table 2. Eligibility criteria

**Inclusion criteria**	**Exclusion criteria**
Staff at UCLH	Contraindication for dietary intervention
Adults aged ≥18 years	Participation in another clinical intervention trial
BMI ≥ 25 kg/m^2 (living with overweight or obesity)	Concomitant recent usage of medications that cause weight gain or weight loss
Weight stable (≤5 % variation in body weight over preceding 3 months)	Cardiometabolic comorbidities (e.g. diabetes, on insulin)
Have a habitual dietary intake consisting of ≥50% UPF intake as % total daily energy intake	Coeliac disease
Able to read and write in English	Inflammatory bowel disease
Medically safe to participate in a dietary intervention programme.	A diagnosed eating disorder
Willing and able to give written informed consent.	Planning a weight management programme in the next 3 months
Able to attend the relevant in person and remote sessions	Any diagnosed food allergy, or other allergies which limit the ability to adhere to the intervention diet
Able to comply with the study protocol (including dietary recommendations for each intervention and reporting adherence)	Dietary restrictions (e.g., vegan, vegetarian, Halal or kosher) which limit the ability to adhere to the interventions
Females of childbearing potential and males agree to use an effective method of contraception from the time consent is signed until the end of the intervention period and final follow-up assessment. Effective methods of contraception acceptable for UPDATE are outlined in the Supplementary Material 3	BMI >40 kg/m^2 or basal metabolic rate ≥2300/kcal/day (to ensure intervention diets are at least 300kcal/day greater than maintenance energy needs, based on excess energy intakes reported in Hall et al.[21])

For peer review only - http://bmjopen.bmj.com/site/about/guidelines.xhtml

Females of childbearing potential must be on highly effective contraception and have a negative pregnancy test	Females who are pregnant, breast-feeding, or intends to become pregnant
	A history of drug or alcohol abuse
	Any other factor making the participant unsuitable in the view of investigator

Participants will complete two non-consecutive 24-hour recalls at screening. If they meet all inclusion criteria and do not meet any exclusion criteria, they will complete a further two non-consecutive 24-hour recalls at baseline to confirm a habitual dietary intake of ≥50% UPF. After which, they will be considered eligible.

Interventions

Eligible participants will receive in a random order (1): an 8-week MPF diet (at least 80% MPF (NOVA group 1), <20% UPF), and (2): an 8-week UPF diet (at least 80% UPF, <20% MPF), both following EWG recommendations. During the 4-week washout between diets, participants will return to their habitual diet to minimise carryover effects.

Participants will be given all meals, snacks and drinks for each 8-week intervention to maximise adherence, ensure internal validity and minimise dropout[29,30]. Catering companies and supermarkets will deliver diets to participants' homes, two times per week. Deliveries will be scheduled according to participants' convenience.

Both diets will follow EWG recommendations, choosing foods with green or amber front of package (FoP) traffic lights for total fat, saturated fat, sugar and salt over foods with red traffic lights, consuming 5 portions per day of fruit and vegetables, and eating a variety of foods in the right proportions[18].

Diets will be matched for and aim to follow government recommended intakes for a 2000kcal/day diet[4,18] (Table 2). Provided diets will be scaled up to 4000kcal/day, to ensure participants are not energy restricted and energy intake is *ad libitum.*

Table 2. Eatwell Guide dietary recommendations[4,18]

**Component**	**Guidance**
Total fat	35% of provided energy intake or below, 78g or less/2000kcal
Saturated fat	10% of provided energy intake or below, 24g or less/2000kcal
Salt	Less than 6g per day. Intake in line with current UK average intakes[31], aiming for below 6g per day
Carbohydrate	Around 50% of provided energy intake, 267g/2000kcal
Total sugars	Less than 90g/2000kcal, less than 18% of total energy
Protein	Around 15% of provided energy intake, 45g/2000kcal
Fibre	At least 18g/2000kcal
Fruit and vegetables	Five portions per day

UK, United Kingdom

For peer review only

The menus will be designed to be representative of UK diets, by identifying the most commonly consumed foods in the UK National Diet and Nutrition Survey[32]. Practical and logistical aspects including price, best-before dates, storage requirements, and accessibility will be factored into the design. Diets will be matched where possible, with MPF/UPF versions of the same meals (e.g., Caesar salad, salmon and potatoes, porridge/breakfast cereal). UPF items are typically foods obtained from supermarkets, whereas MPFs are freshly made culinary preparations. The menu will vary across the week to prevent participant boredom and sensory-specific satiety[33], but repeating each week to allow consistency and reduce burden. Alcohol will not be provided. Participants will be asked to keep alcohol consumption within government guidelines (≤14 units per week)[34]. Participants will be educated on the EWG, but further lifestyle guidance will not be given. Menu guides will be provided with instructions and pictures to prepare each meal. As in previous *ad libitum* feeding trials investigating weight change[35], participants will be asked to consume as much or as little of the provided diets as they desire, but they will not be asked to actively reduce their energy intake.

Participants will receive weekly calls the research team, including dietitians, to discuss any issues with the diets and to promote adherence. Participants will have a food diary to track adherence and to record any food consumed off diet. The 4- and 8-week diet assessments will also be used for monitoring adherence. Participants will be encouraged to report any deviations from the provided diets during weekly calls and in the food diary, and to be as honest as possible, with no repercussions. Previous studies indicate high adherence when all food is provided and delivered to participant homes, where eating foods provided is more of a challenge than eating foods off the diet[29]. Minor modifications to the intervention that do not alter the overall design will be acceptable for enabling adherence. For example, adding herbs or spices (without sugar/salt) to taste, or providing alternative items to facilitate food preparation if participants do not have access to a microwave (e.g. swapping a microwaveable meal for a ready-to-eat meal).

Behavioural intervention

The study is powered for the crossover RCT, rather than for the behavioural support intervention. Therefore, in line with the Medical Research Council (MRC) guidance for developing complex interventions[36], the aim of this secondary aspect of the study is to explore the feasibility and acceptability of the behavioural support intervention to reduce UPF (primary target behaviour) and increase physical activity (secondary target). Full details of the intervention development and content will be provided in a future publication focussed on the behavioural support intervention. However, in brief, the intervention was developed following the steps outlined in the Behaviour Change Wheel framework for intervention development[37], which incorporates the "Capability, Opportunity, Motivation" model for understanding behaviour (COM-B), augmented with Theoretical Domains Framework (TDF) for utilising behaviour change theory[36,38]. Qualitative studies of barriers and facilitators for dietary change in healthcare workers were extensively reviewed, with findings then mapped onto the TDF, and behaviour change techniques consistently associated with successful diet and physical activity change drawn from meta-analyses of RCTs (e.g.[39–41]). The intervention content combines monthly video/telephone behavioural support calls with print/online resources, based on TDF domains. Some examples of TDF domains (linked to the intervention components) are Knowledge (information on what UPFs are and how to recognise them),

For peer review only

Environment (mapping of food outlets local to participant's workplace to for quickly accessible MPF options), Beliefs about Consequences (information about links between UPF and health), Behavioural Regulation (support with goal-setting, action-planning, self-monitoring, habit) and Social Influence (moderated online peer support sessions). The calls will be individually tailored based to participant demographics/work patterns, responses to their baseline COM-B questionnaire, baseline levels of UPF intake and reported physical activity (measures of these are all described in the outcomes section) and personal barriers and motivations to change.

Outcomes

Outcome measures will be collected at seven in-person visits. See Figures 1 and 3, and Supplementary Material 4 for the Schedule of Assessments.

The primary outcome is the mean difference in percent weight change (%WC) at 8 weeks from baseline between MPF and UPF diets. This outcome is currently being used clinically in weight management clinics, used for all NHS weight management programmes and directly relates to improvements in cardiometabolic risk factors (e.g., blood pressure, blood glucose, glycated haemoglobin (HbA1c), lipids)[42], physical function and quality of life[43]. Weight is an efficient and more accurate measurement to collect than other energy balance measures, such as energy intake. The hypothesised %WC in the face of the current growing obesity pandemic would have clinically relevant impacts on halting the rate of progression of obesity prevalence and mitigating the adverse impacts of adiposity-related disease, simply through shifts in the types of processed in foods being consumed.

Secondary outcomes include changes in dietary intake, waist circumference, body composition (fat mass, fat-free mass), heart rate, blood pressure, cardiometabolic risk factors (blood biomarkers such as HbA1c and c-reactive protein), fasted and fed metabolomics, appetite measures (fasted and fed appetite scores, circulating gut hormones and adipocytokines), sleep quality, physical activity levels, physical function and strength, mental health and wellbeing and aspects of eating behaviour between MPF and UPF diets, and after the 6-month behavioural intervention compared with the first baseline assessment. In a subset of participants, changes in brain functional resting state connectivity between MPF and UPF diets will be assessed. Further behavioural intervention secondary outcomes include understanding the experiences of UPF versus MPF diets, and barriers and facilitators to reducing UPF intake.

Meal tests with blood samples for biomarkers, metabolomics, gut hormones and adipocytokines will be collected at five visits: baseline visits and 8-weeks follow-ups for the RCT and at 6-months follow-up for the behavioural intervention. A random subset of participants will undergo MRI scans at four visits: baseline visits and 8-weeks follow-ups for the RCT. The COM-B behaviour change questionnaire will be assessed at two visits: the first baseline visits and at 6-months follow-up.

Participant timeline

For peer review only

The trial duration per participant is 49 weeks (see Figure 2). The overall trial is expected to last 24 months. Recruitment commenced in March 2023, and is expected to continue until March 2024 or when 55 participants have been recruited.

Figure 2. Participant timeline

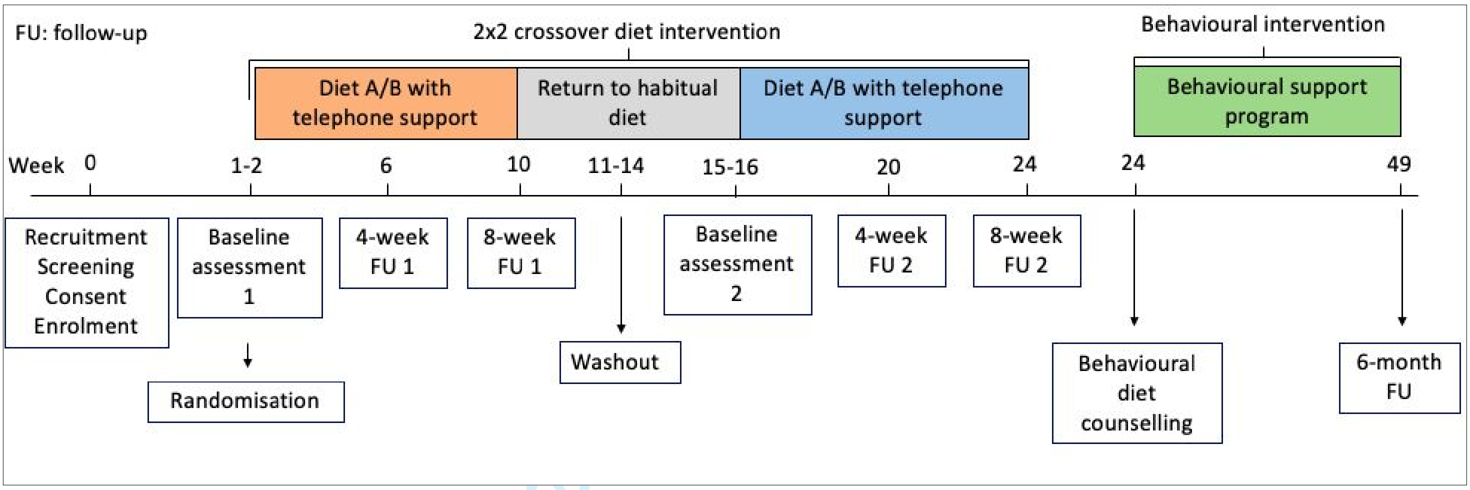

FU, follow-up.

Methods: Assignment of interventions

Randomisation

Enrolled participants will be block randomised (stratified by night-shift status, sex and ethnicity as potential treatment modifiers) to receive: (1) the MPF diet then UPF diet, or (2) UPF diet then MPF diet. Randomisation will be performed using Sealed Envelope by the research team (https://www.sealedenvelope.com).

The trial statistician, but not researchers, will be blinded to the diet assignments. To prevent bias, participants are informed that the provided interventions are healthy balanced diets made with different types of food processing. All participant communications omit the terms MPF/UPF, with diets instead being referred to generally as Diet 1/A or Diet 2/B. Unblinding procedures are not required.

Methods: Data collection, management, and analysis

Data collection methods

Sociodemographics

Age, gender, ethnicity, occupation, work pattern, educational level, marital status, medication intake, alcohol consumption, smoking habits, family history of obesity, cardiovascular disease and diabetes will be collected at screening.

Anthropometrics and body composition

Weight will be measured using an electronic scale to the nearest 0.1 kg (Tanita DC-430MAS; Tanita, Tokyo, Japan). Body composition including fat mass, fat-free mass and bone mass will be assessed using bioelectrical impedance analysis (BIA) (Tanita) at each visit. Height will be determined using a stadiometer to the nearest 0.1 cm, and BMI derived from weight and

height (in kg/m^2). Waist circumference will be measured using a non-stretch tape measure at the iliac crest[44].

Vital signs

Blood pressure will be recorded in triplicate, seated, alongside heart rate with an automated sphygmomanometer and oximeter.

Mixed meal test and blood samples

Mixed meal tests will be used to assess circulating gut hormones, adipocytokines and metabolomics, given the significant differences in metabolites and appetite hormones between UPF and MPF diets in Hall et al., 2019[21]. Participants will consume 187.5ml Ensure Compact 2.4kcal (https://nutrition.abbott/uk/product/ensure-compact) after an overnight fast. Fasted blood samples taken before the test include venous glucose, HbA1c, liver function tests, lipid profile and C-reactive protein, relevant for cardiometabolic health. Blood samples and subjective appetite assessments using a visual analogue score will be collected immediately before, and 15 and 30 minutes after starting the liquid meal[45]. Fasted and post-prandial circulating appetite hormones (ghrelin, peptide YY and glucagon-like-peptide-1), adipocytokines as markers of adipose tissue inflammation (leptin, resistin, adiponectin, interleukin-6 and tumour necrosis factor alpha), and metabolomics analysis of 250 metabolites using the Nightingale platform (https://nightingalehealth.com) will be collected and stored at -80°C.

Physical activity levels and sleep quality

Physical activity (PA) levels will be measured objectively using accelerometry (ActiGraph wGT3X-BT). The device has been previously used in clinical research given its practicality, non-invasiveness, and reliability and accuracy in measuring PA in free-living adults[46]. Data recorded includes energy expenditure, metabolic equivalents and sleep activity. The short-form International Physical Activity Questionnaire (IPAQ-SF) will be used to subjectively assess light, moderate and vigorous PA, and time spent sitting[47–49]. The IPAQ-SF contains seven questions about PA in the past 7 days[50]. ActiGraph wGT3X-BT will also objectively measure sleep quality and quantity. The Pittsburgh Sleep Quality Index is a validated 19-item measure for determining sleep quality in the past month, to distinguish between good and poor sleepers[51].

Physical function and strength

Static muscle strength of the upper extremities will be assessed using a handgrip dynamometer (Jamar Hydraulic Hand Dynamometer, Patterson Medical). Three measures will be recorded (in kg) with each hand, alternately, whilst seated. Functional capacity will be assessed using the 6-minute walk test, a self-paced submaximal assessment, validated in people living with obesity[52]. The test will be performed according to the *"American Thoracic Society Statement: Guidelines for the Six-minute Walk Test"* protocol[53]. The pre- and post-test heart rate, total distance covered, perceived physical exertion and any physical problems will be recorded. Lower body functional capacity will be assessed using the sit-to-stand test[54]. Participants will be requested to perform five sit-to-stand repetitions as fast as possible with arms crossed over the chest. Number of repetitions and time to completion will be recorded.

Quality of life, mental health and wellbeing questionnaires

For peer review only

EuroQol five-dimensions three-levels (EQ-5D-3L) is a 6-item questionnaire containing a descriptive system assessing five domains: mobility, self-care, usual activities, pain/discomfort and anxiety/depression using three levels (e.g., mobility: I have no problems walking about, I have some problems walking about, or I am confined to bed) and a visual analogue scale reporting self-rated health from 0-100[55]. Impact of Weight on Quality of Life-Lite (IWQOL-Lite) is a 31-item, obesity/overweight-specific measure of health-related quality of life relating to five domains: physical function, self-esteem, sexual life, public distress, and work. Higher scores indicate a better quality of life[56]. The Warwick Edinburgh Mental Wellbeing Scale (WEMWBS) is a 14-item, positively worded, validated measure of mental wellbeing. Five responses are summed for a total score between 14-70, with higher scores indicating greater positive mental wellbeing[57]. The Patient Health Questionnaire (PHQ-9) is a 9-item validated measure to assess the severity of depression[58]. Each item is rated from 0-3, for a total score out of 27. A score of 1-4 indicates minimal depression, 5-9 indicates mild, 10-14 indicates moderate, 15-19 indicates moderately severe, with 20-27 indicating severe depression. The Generalised Anxiety Disorder assessment (GAD-7) is a 7-item anxiety scale to measure the severity of generalised anxiety disorder[59]. Each item is rated from 0-3, for a total score out of 21. A score of 11-15 indicates moderately severe anxiety, with 16-21 indicating severe anxiety.

Eating behaviour questionnaires

The Power of Food (PoF) scale is a 15-item validated measure to assess the psychological impact of living in food-abundant environments[60]. The Control of Eating Questionnaire (CoEQ) is a 21-item validated measure of the severity and type of food cravings[61].

Behaviour change questionnaire

Participants will complete a questionnaire investigating the barriers and facilitators to PA and healthy eating based on COM-B[62]. The PA component contains 137 items, and the eating component contains 120 items.

Magnetic resonance imaging (MRI)

Several aspects of UPFs may influence eating behaviour, including changes to the food composition, food matrix degradation and behavioural aspects, including large portion presentation, cost, availability, shelf life, heavy marketing and attractive packaging[16]. UPFs have been suggested to have addictive-like properties[63], evoking strong emotional reactivity from visual cues[64]. Functional brain magnetic resonance imaging (MRI) has provided valuable insights into appetite regulation[65], and how obesity impacts the neurobiology of weight regulation. However, the neurobiological impact of high-UPF diets is largely unknown. A subset of participants (*n*=24) will undergo an advanced MRI brain protocol at the baseline and 8-week visits of the RCT (four visits), to assess a number of changes in brain properties: i) functional resting-state connectivity between regions implicated in eating behaviour, metabolism and swelling linked to diffuse inflammation, and their reversibility[66]; ii) microstructure brain properties extracted from diffusion weighted imaging data[67]; iii) changes in brain dynamics, based on each subject's functional and structural connectivity[68]; iv) metabolite changes, such as glutamate, N-acetyl aspartate and Inositol, through brainstem magnetic resonance spectroscopy[69]; v) regional volume changes through high-resolution structural scans[66], and vi) relaxometry properties alterations affected by inflammation[70].

For peer review only

Assessment of dietary adherence and intake

In line with previous trials[71], multiple approaches will be used to assess dietary intake and adherence, including 24-hour recalls, food frequency questionnaires (FFQ) and image-based dietary assessment (IBDA).

Intake24[72] is a validated, online, self-reported 24-hour recall system, based on a multiple-pass recall suitable for the general population[73,74] (https://intake24.co.uk). The web-based recall method is convenient, efficient and ensures coding consistency. Participants enter all food and drink consumed in the previous 24 hours (from waking up to going to sleep) into Intake24 on two non-consecutive days per visit period. Each recall takes roughly 12 minutes to complete[75]. The first recall at screening will be conducted with the research team to ensure adequate training. Links will then be sent to participants to complete further recalls remotely. Intake24 is connected to the National Diet and Nutrition Survey Nutrient Databank to provide nutrient outputs, and has been coded into NOVA by the research team for calculating UPF/MPF intake.[76].

The European Prospective Investigation into Cancer and Nutrition (EPIC)-Norfolk FFQ is a validated, semi-quantitative measure of average dietary intake over the past year[77–79]. The FFQ contains a 130-item food list followed by detailed questions regarding the food list. For each food item, participants tick the most appropriate frequency of consumption of that item from nine options (from never or less than once per month, to 6+ per day).

Participants will complete an FFQ and two non-consecutive day 24-hour dietary recalls at baseline, and 4- and 8-weeks for both diets, and at the 6-month behavioural intervention follow-up (Figure 1)[80]. IBDA will also be completed on two non-consecutive days during weeks 4 and 8 of the RCT, whereby participants will take photos before and after their *ad libitum* meals/snacks.

Questionnaires summary

There are twelve questionnaires/surveys: PSQI, IPAQ-SF, EQ-5D-3L, IWQOL-Lite, WEMWBS, PHQ9, GAD7, PoF, CoEQ, COM-B healthy eating and PA questionnaire, Intake24 and EPIC-Norfolk FFQ. Time points for completion are detailed in Table 3 and in Supplementary Material 4.

Feasibility/acceptability and process evaluation of the behavioural intervention

Feasibility/acceptability and process evaluation will be described in full in the subsequent paper focussed on the behavioural intervention. However, the main feasibility/acceptability outcome will be percentage of intervention calls successfully delivered, as well as retention to the study. For quality control intervention calls will be recorded and coded against a checklist to ensure that target BCTs were being delivered as planned. Acceptability of the intervention content and process will be gathered in qualitative interviews. Changes in other outcomes pre and post the behavioural intervention will be explored as secondary.

Qualitative interviews

In the first behavioural intervention call, before being told that the intervention(s) focus on processing, participants will be asked to describe their experiences of each of the trial diets 1A/2B (positives and negatives, how they felt physically/mentally, barriers they faced to

For peer review only

adherence) then probed on which diet they thought was UPF vs MPF and why. In addition, at the end of the behavioural intervention participants will be invited to take part in a one-to-one semi-structured telephone interview about their experiences of the trial overall, motivations for participation, barriers to reducing UPF and increasing MPF, and experience of the behavioural intervention specifically (whether they found materials and process acceptable/useful).

Table 3: Time points of questionnaires in the UPDATE trial.

Visit	Number of questionnaires	Time to complete
Screening	Intake24 only	12 minutes
RCT:<br>• First baseline visit<br><br>Behavioural intervention:<br>• 6-month follow-up<br><br>(Visits 2 and 9)	All 12 questionnaires	90 minutes
RCT:<br>• Week 4<br>• Week 8<br>• Second baseline visit<br><br>(Visits 3, 4, 5, 6 and 7)	11 questionnaires (all except the COM-B healthy eating and PA questionnaire)	60 minutes

COM-B, Capability, Opportunity, Motivation - Behaviour; PA, physical activity; RCT, randomised controlled trial.

Data methods: monitoring

Data monitoring

Data generated from this trial will be handled (including collection, storage, processing and disclosure) in accordance with all applicable legal and regulatory requirements, including the UK Data Protection Act (DPA) 2018 and European Union General Data Protection Regulation (EU GDPR 2016/679). All members of the research team are trained in information governance and research integrity.

All data storage mediums will comply with the NHS Information Governance Toolkit. All physical data will be stored in a secure room, with limited access only to members of the research team. All computers storing electronic data will be encrypted and password protected. Data will be first stored on paper case report forms (CRF), securely kept in a locked cabinet in a locked office, and then recorded electronically on a purpose-built trial database (eCRF) using the REDCap system (https://www.project-redcap.org). REDCap is hosted within the UCL Data Safe Haven, a ISO27001 certified secure environment protected by dedicated firewalls, accessed by role-based user accounts with multi-factor authentication. Each participant has their own eCRF, listed under their participant identification number (PIN).

For peer review only

Questionnaires will be provided electronically and stored directly in REDCap. The REDCap database contains range validity checks with warnings for erroneous or missing values, and initial data entry from the paper CRF will be verified by a second team member for quality assurance. The REDCap application also provides a comprehensive audit trail showing all changes to the data.

Data confidentiality is outlined in the PIS (Supplementary Material 1). Participant initials and their PIN will be used on records. The CRF will not bear the participant's name or other personal identifiable data. Identifiable information will be stored in a separate electronic database to the database containing trial data. Participants' personal identity and data collected in the study cannot be connected by anyone outside the study team. The data generated from this trial will not be transferred to any party not identified in the protocol and will not be processed and/or transferred other than in accordance with the participants' consent. Confidentiality will be maintained by user agreements prior to access, ensuring all researchers uphold the principles of Good Clinical Practice, EU GDPR 679/2016 and DPA 2018.

Retention

Loss to follow-up will be minimised through weekly telephone calls during the RCT and monthly calls during the 6-month behavioural intervention to discuss any issues and adherence, encouraging participants to discuss any difficulties with researchers. Other procedures will be adopted for maintaining participation in the study (e.g., reminders before scheduled trial visits, sending greetings messages, personalising letters, and keeping measures short in terms of completion time). No data will be collected after withdrawal.

Sample size

The sample size of 55 is based on Hall et al., 2019[21] showing 0.9kg weight loss following a 2-week MPF diet, with a standard deviation (SD) of the mean difference in weight change of 1.98kg between MPF and UPF diets (mean: 1.85kg), the predicted weight loss if the MPF diet were continued for 8 weeks [using the National Institutes of Health (NIH) bodyweight planner (https://www.niddk.nih.gov/bwp)].

No interventional studies to date have compared diets differing in the nature, extent and purpose of food processing over this duration. To best estimate the expected difference in %WC over this time-frame, the results from the only controlled feeding trial comparing UPF and MPF diets matched for diet quality on weight change were used (Hall et al., 2019[21]), which lasted for 2 weeks. Inclusion criteria for UPDATE requires participants to have a habitual diet high in UPF, thus the UPF diet is hypothesised at best to lead to no change in weight. Whereas, the MPF diet will result in weight loss, with a similar initial trajectory to that in Hall et al., 2019[21]. The NIH bodyweight planner takes into account that weight change is not linear based on the initial calorie deficit and tends to plateau and considers key factors that influence metabolic rate. The mean age, height, weight, and physical activity levels of the male and female participants from Hall et al., were entered into the bodyweight planner to produce a quantitative estimate of weight change on the MPF diet over 8 weeks, which was converted percentage weight change. Male participants in Hall et al., 2019[21] would achieve 2.8% (2.2kg) weight loss after 8 weeks on the MPF diet, and female participants would achieve 2.7% (2.1kg) weight loss. There is no clear value from the literature as to what the SD should be for the mean difference in weight change between 8-week MPF and UPF diets. The expected SD must

For peer review only

therefore be estimated. The 1.98kg value for the SD of the mean difference in weight change from Hall et al., was used as the starting point for the expected SD after 8 weeks. This was around 1.1x the mean difference in weight change between MPF and UPF diet interventions. By assuming that the SD would increase over 8 weeks compared with 2 weeks, the SD was assumed to increase to 2x the mean difference in weight change between MPF and UPF diets. Other trials longer in duration than Hall et al., 2019 were considered, but other trials have a number of fundamental differences to the planned trial, which limits the ability to extrapolate their findings [e.g. not necessarily *ad libitum* or crossover trials, testing diets that are unrelated to the concepts of MPF or UPF, or comparing an intervention to a control diet, (whereas UPDATE involves two interventions)]. These generally reported SDs for the mean weight change of each group, rather than of the mean difference between groups. For each group over several weeks (e.g., 12 weeks), the SDs tended to be around 0.1-0.9x the mean weight change. Therefore, the Hall et al., 2019 estimate was considered most applicable, relevant and conservative.

Assuming weight loss on the MPF diet and no weight change on the UPF diet, 44 participants are required to detect a mean difference of 2.7% weight change between diets (standard deviation of 5.4% (2x the mean difference), power=0.9, alpha=0.05, with a two-sided paired t-test, using SPSS version 27.0). With a 20% dropout rate based on previous controlled feeding trials[81,82], 55 participants will be recruited. This is comparable to attrition rates in previous multi-arm, community-based crossover trials lasting several months, where participants are provided with all meals[83–86].

The target sample size for the qualitative interviews is 20-30. Data saturation is controversial, but researchers will aim for meaningful saturation, whereby further interviews produce minimal, or no changes to the coding framework and allow complete understanding of thematic codes[87]. Where possible, participants will be selected to ensure representation from across ethnicities, night-shift patterns genders, treatment allocation arms. All participants will be offered the behavioural intervention calls, so will be asked about experiences of the provided trial diets.

Statistical methods

A CONSORT diagram and descriptive statistics will be used to outline the trial sample[88]. The primary outcome will be analysed using mixed-effects models, with a random effect for participants, adjusting for stratification variables and any baseline variables not balanced between arms. An intention-to-treat analysis will be conducted[89], with values presented by randomisation group and all available data analysed as randomised. Additional models will include a per-protocol analysis, and repeated-measures analysis additionally using data from 4-week follow-ups. Bias from missing data will be dealt with using multiple imputation[90]. The 4-week washout period between diets will minimise any carryover effects[88], with any residual effect assessed between arms. To account for non-adherence (consuming more than one meal per week off the intervention diet), inverse probability weighting will be used to reweight the remaining sample[91]. There is no planned interim analysis.

Secondary outcome variables for the RCT and behavioural intervention will be analysed using mixed-effects or linear, binary or ordinal regression models, where appropriate.

For peer review only

Qualitative interviews will be analysed using Framework Analysis, following the stages outlined by Gale and colleagues[92]. Transcripts will be deductively coded, broadly mapping barriers and facilitators to the COM-B model[36,37] and Theoretical Domains Framework[38] – an approach previously used to understand the experience of novel diets[93], as well as inductively coding aspects that may not naturally fit into these groupings. Recordings will be converted into text using transcription software. Two researchers will initially independently analyse three interviews, before meeting to discuss and develop an initial framework. Additional transcripts will then be coded based on this framework, with additional factors added as they arise. Two researchers will interpret data and write the report in accordance with the COREQ checklist (www.equator-network.org/reporting-guidelines/coreq).

Methods: Monitoring

A Data and Safety Monitoring Plan (DSMP) plan is in place for quality control to ensure adherence to the approved protocol (Version 1.3, 26/07/23). Charters outline the roles and responsibilities of the Trial Management Group (TMG) and Trial Steering Committee (TSC). The TMG includes the Chief Investigator (CI) (RLB), Trial Coordinator and Manager (SJD) and co-investigators/researchers (AB, CWK, AF, FCJ, JM). The TMG is responsible for maintaining and overseeing the trial, meeting before recruitment and every 3 months throughout the trial. The TMG approved the final trial protocol and eCRF, as well as any subsequent amendments. The TSC will provide supervision and act on behalf of the funders and Sponsor, recommending any appropriate amendment/actions as necessary. The TSC includes a lay member and will be chaired by an external academic member with experience leading clinical trials. The trial may be stopped before completion upon recommendation from the TSC, or Sponsor and CI.

A Data Monitoring Committee will not be set up as there are no intervention-related adverse events (AEs), serious adverse events (SAEs) or major risks associated with participation. Risks are considered minimal, and are outlined in the PIS and CF (Supplementary Materials 1 and 2). Recording/reporting AEs will commence from written informed consent until the 6-month follow-up (week 49). All SAEs will be reported to the Sponsor within 24 hours of becoming aware. Incidental findings will be reported to participants and their GP.

Auditing

Monitoring and auditing (e.g., adherence, deviations, withdrawals, AEs/SAEs) is outlined in the DSMP, and will be conducted by the CI, TMG and TSC in accordance with the UK Policy Framework for Health and Social Care Research, and in accordance with the Sponsor's monitoring and audit policies and procedures.

Ethics and dissemination

Research ethics approval

The Yorkshire & The Humber - Sheffield Research Ethics Committee approved the trial on 22nd December 2022 (22/YH/0281), and the study was prospectively registered on ClinicalTrials.gov (NCT05627570). The trial is currently recruiting and ongoing. The trial will be conducted in compliance with the principles of the Declaration of Helsinki 1996 and the principles of the International Conference on Harmonisation GCP. Any amendments will be

For peer review only

recorded in academic publications, and will be submitted for approval to the Sponsor and Research Ethics Committee prior to implementation. Protocol Version 1.3, 26/07/2023. Sponsor contact: (UCLH/UCL) Joint Research Office (uclh.randd@nhs.net). On 16/06/2023, the UPF intake inclusion criterion was amended from ≥60% to ≥50% to better reflect the average intake of participants in London, compared with intakes across the UK[9].

Ancillary and post-trial care

There will be no diet provisions or behavioural support given at the end of the trial. Consent will be sought for future research and the storage and future use of participant samples in the Obesity Research Biobank Syndicate (ORBiS).

Dissemination

Results will be disseminated through publications, conferences and social and lay media. Progress and analyses may be presented to the Sponsor and funding bodies. Authorship will follow the International Committee of Medical Journal Editors guidelines. Participant data will not be made publicly accessible.

Discussion

This protocol outlines the methodology for a free-living study comparing the health effects of MPF and UPF diets following EWG dietary recommendations, and the effectiveness of a 6-month behavioural support programme to reduce UPF. If the hypotheses are supported, the results have the potential to substantially impact on dietary guidelines and the dietary management of obesity worldwide, supporting the development of regulations around the marketing and labelling of UPFs, and increasing knowledge and public awareness of the health consequences of UPFs[94]. The results are directly relevant for UK public health food policy as the interventions are based on the UK EWG. Given the similarities between UK and other national dietary guidelines, there will be important implications for guidelines worldwide as well.

Importantly, the results would highlight important health implications of the foods that make up the majority of the energy intake of the UK adult population and the differential impact of UPF and MPF, regardless of following current NHS dietary guidance.

However, resource limitations mean that individuals with dietary restrictions (e.g., vegan, vegetarian, Halal, Kosher) are excluded, and the intervention diets are not adapted to individual energy needs, as it was not financially or logistically possible to draft a range of matched diets across both intervention arms.

Twitter

R.L.B: ProfBatterhamMD
S.J.D: SamuelDickenUK
A.B: BrownAdey
C.W.K: Claudia_WK; @GolgiBrain
K.H: KevinH_PhD
A.F: abifisher99

For peer review only

J.M: DrJanine_Mak
F.C.J: FriedrichJassil
C.v.T: DoctorChrisVT

Author contributions:

R.L.B conceptualised the trial; S.J.D wrote the initial manuscript and protocol draft; R.L.B, S.J.D, A.B, C.W.K, K.H, A.F, J.M, F.C.J and C.v.T contributed to the intervention design, methodology and assessments, R.L.B, S.J.D, A.B, A.F, J.M and F.C.J contributed to the study protocol.

Acknowledgements:

The authors would like to thank: UPDATE participants and collaborators, the Intake24 team (https://intake24.co.uk/), the patient and public involvement focus group, EPIC-Norfolk for use of their FFQ (https://www.epic-norfolk.org.uk/for-researchers/ffq/), REDCap (https://www.project-redcap.org/) and Out Of The Box Food. Acknowledgements also go to Caroline Buck, Gabriella Heuchan and Rana Conway, for their substantial input into the behavioural intervention development.

The statistical analysis plan has been reviewed by an independent associate professor in statistics, (with thanks to Michalis Katsoulis) and a statistician specialising in clinical trial design was consulted regarding the sample size calculation for the proposed statistical analyses (with thanks to Michael Grayling).

Funding:

This work was supported by National Institute for Health and Care Research Biomedical Research Centre (NIHR BRC) grant BRC530a and Rosetrees Trust grant PGL22/100041.

Patient and public involvement:

The trial, including the menu, assessments and participant burden have been reviewed and designed with input from NHS UCLH staff at following a focus group session before ethics submission. In addition, Obesity Empowerment Network UK members, with lived experience of obesity, have also contributed to the design.

Competing Interests:

S.J.D is funded by the Medical Research Council (MR/N013867/1) and receives royalties from Amazon for a self-published book that mentions ultra-processed food, and payments from Red Pen Reviews as a contributor. R.L.B reports honoraria from Novo Nordisk, Eli Lilly, Medscape, ViiV Healthcare Ltd and International Medical P and advisory board and consultancy work for Novo Nordisk, Eli Lilly, Pfizer, Gila Therapeutics Ltd, Epitomee Medical Ltd and ViiV Healthcare Ltd and from May 2023 is an employee and share holder of Eli Lilly and Company. A.B. reports honoraria from Novo Nordisk, Office of Health Improvement and Disparity, Johnson and Johnson and Obesity UK outside the submitted work and is on the

For peer review only

Medical Advisory Board and shareholder of Reset Health Clinics Ltd. CGWK receives funding from Horizon2020 (Research and Innovation Action Grants Human Brain Project 945539 (SGA3)), BRC (#BRC704/CAP/CGW), MRC (#MR/S026088/1), Ataxia UK, Rosetrees Trust (#PGL22/100041 and #PGL21/10079) and is a shareholder in Queen Square Analytics Ltd. JM is funded by the NIHR and reports funding from the NIHR BRC and the Society from Endocrinology. JM reports institutional funding from Novo Nordisk, Rhythm Pharmaceuticals and Innovate UK outside the submitted work.KH is supported by the Intramural Research Program of the National Institutes of Health, National Institute of Diabetes & Digestive & Kidney Diseases. C.v.T receives royalties for a book on ultra-processed food. A.F. and F.C.J report no conflicts of interest.

Peer review:

The study has been peer reviewed in accordance with the requirements outlined by UCL. The study was internally peer-reviewed by co-investigators of the research team, and externally peer-reviewed by two peer reviewers at UCL. The protocol has been updated with additional detail following the comments provided by the peer reviewers. Furthermore, the trial was peer-reviewed as part of the funding grant application by Rosetrees Trust by external peer reviewers.

References:

1 NHS Digital. Part 3: Adult overweight and obesity. 2020.https://digital.nhs.uk/data-and-information/publications/statistical/statistics-on-obesity-physical-activity-and-diet/england-2020/part-3-adult-obesity-copy (accessed 12 Jul2021).

2 The Global BMI Mortality Collaboration. Body-mass index and all-cause mortality: individual-participant-data meta-analysis of 239 prospective studies in four continents. *Lancet* 2016; **388**: 776–786.

3 Frontier Economics. The annual social cost of obesity in the UK. Frontier Economics. https://www.frontier-economics.com/uk/en/news-and-articles/articles/article-i9130-the-annual-social-cost-of-obesity-in-the-uk/ (accessed 26 Jul2023).

4 Public Health England. Government recommendations for energy and nutrients for males and females aged 1–18 years and 19+ years. 2016.https://assets.publishing.service.gov.uk/government/uploads/system/uploads/attachment_data/file/618167/government_dietary_recommendations.pdf (accessed 14 Oct2021).

5 Popkin BM, Adair LS, Ng SW. The Global Nutrition Transition: The Pandemic of Obesity in Developing Countries. *Nutr Rev* 2012; **70**: 3–21.

6 Popkin BM, Ng SW. The nutrition transition to a stage of high obesity and noncommunicable disease prevalence dominated by ultra-processed foods is not inevitable. *Obesity Reviews* 2022; **23**: e13366.

For peer review only

7 Hall KD. From dearth to excess: the rise of obesity in an ultra-processed food system. *Philosophical Transactions of the Royal Society B: Biological Sciences* 2023; **378**: 20220214.

8 Monteiro CA, Cannon G, Levy RB, Moubarac J-C, Louzada ML, Rauber F *et al.* Ultra-processed foods: what they are and how to identify them. *Public Health Nutr* 2019; **22**: 936–941.

9 Rauber F, Steele EM, Louzada ML da C, Millett C, Monteiro CA, Levy RB. Ultra-processed food consumption and indicators of obesity in the United Kingdom population (2008-2016). *PLoS One* 2020; **15**. doi:10.1371/journal.pone.0232676.

10 Moradi S, Entezari MH, Mohammadi H, Jayedi A, Lazaridi A-V, Kermani M ali H *et al.* Ultra-processed food consumption and adult obesity risk: a systematic review and dose-response meta-analysis. *Crit Rev Food Sci Nutr* 2021; : 1–12.

11 Askari M, Heshmati J, Shahinfar H, Tripathi N, Daneshzad E. Ultra-processed food and the risk of overweight and obesity: a systematic review and meta-analysis of observational studies. *Int J Obes (Lond)* 2020; **44**: 2080–2091.

12 Juul F, Deierlein AL, Vaidean G, Quatromoni PA, Parekh N. Ultra-processed Foods and Cardiometabolic Health Outcomes: from Evidence to Practice. *Curr Atheroscler Rep* 2022; **24**: 849–860.

13 Isaksen IM, Dankel SN. Ultra-processed food consumption and cancer risk: A systematic review and meta-analysis. *Clin Nutr* 2023; **42**: 919–928.

14 Lane MM, Gamage E, Travica N, Dissanayaka T, Ashtree DN, Gauci S *et al.* Ultra-Processed Food Consumption and Mental Health: A Systematic Review and Meta-Analysis of Observational Studies. *Nutrients* 2022; **14**: 2568.

15 Zhang S, Gu Y, Rayamajhi S, Thapa A, Meng G, Zhang Q *et al.* Ultra-processed food intake is associated with grip strength decline in middle-aged and older adults: a prospective analysis of the TCLSIH study. *Eur J Nutr* 2021. doi:10.1007/s00394-021-02737-3.

16 Dicken SJ, Batterham RL. The Role of Diet Quality in Mediating the Association between Ultra-Processed Food Intake, Obesity and Health-Related Outcomes: A Review of Prospective Cohort Studies. *Nutrients* 2022; **14**: 23.

17 Scientific Advisory Committee on Nutrition. Carbohydrates and Health Report. 2015.https://www.gov.uk/government/publications/sacn-carbohydrates-and-health-report (accessed 5 Feb2020).

18 NHS. The Eatwell Guide. https://www.nhs.uk/live-well/eat-well/the-eatwell-guide/ (accessed 11 Oct2021).

19 European Commission. Nutrition claims. 2012.https://ec.europa.eu/food/safety/labelling-and-nutrition/nutrition-and-health-claims/nutrition-claims_en (accessed 7 Nov2021).

For peer review only

20 Derbyshire E. Are all ‘ultra-processed’ foods nutritional demons? A commentary and nutritional profiling analysis. *Trends Food Sci Technol* 2019; **94**: 98–104.

21 Hall KD, Ayuketah A, Brychta R, Cai H, Cassimatis T, Chen KY *et al.* Ultra-Processed Diets Cause Excess Calorie Intake and Weight Gain: An Inpatient Randomized Controlled Trial of Ad Libitum Food Intake. *Cell Metab* 2019; **30**: 67-77.e3.

22 Cordova R, Kliemann N, Huybrechts I, Rauber F, Vamos EP, Levy RB *et al.* Consumption of ultra-processed foods associated with weight gain and obesity in adults: A multi-national cohort study. *Clin Nutr* 2021; **40**: 5079–5088.

23 British Nutrition Foundation. Position statement on the concept of ultra-processed foods (UPF) - British Nutrition Foundation. https://www.nutrition.org.uk/news/2023/position-statement-on-the-concept-of-ultra-processed-foods-upf/ (accessed 7 Jul2023).

24 GOV.UK. SACN statement on processed foods and health. https://www.gov.uk/government/publications/sacn-statement-on-processed-foods-and-health (accessed 11 Jul2023).

25 Kyle RG, Wills J, Mahoney C, Hoyle L, Kelly M, Atherton IM. Obesity prevalence among healthcare professionals in England: a cross-sectional study using the Health Survey for England. *BMJ Open* 2017; **7**: e018498.

26 Blake H, Mo PK, Lee S, Batt ME. Health in the NHS: lifestyle behaviours of hospital employees. *Perspect Public Health* 2012; **132**: 213–215.

27 Power BT, Kiezebrink K, Allan JL, Campbell MK. Understanding perceived determinants of nurses’ eating and physical activity behaviour: a theory-informed qualitative interview study. *BMC Obes* 2017; **4**: 18.

28 Chan A-W, Tetzlaff JM, Altman DG, Laupacis A, Gøtzsche PC, Krleža-Jerić K *et al.* SPIRIT 2013 Statement: Defining Standard Protocol Items for Clinical Trials. *Ann Intern Med* 2013; **158**: 200–207.

29 Hall DM, Most MM. Dietary Adherence in Well-Controlled Feeding Studies. *Journal of the American Dietetic Association* 2005; **105**: 1285–1288.

30 Moreira EAM, Most M, Howard J, Ravussin E. Dietary Adherence to Long-Term Controlled Feeding in a Calorie-Restriction Study in Overweight Men and Women. *Nutr Clin Pract* 2011; **26**: 309–315.

31 Public Health England. National Diet and Nutrition Survey: Assessment of salt intake from urinary sodium in adults. https://assets.publishing.service.gov.uk/government/uploads/system/uploads/attachment_data/file/876624/Report_England_Sodium_Survey_2018-to-2019.pdf.

For peer review only

32 NDNS: results from years 9 to 11 (2016 to 2017 and 2018 to 2019). GOV.UK. https://www.gov.uk/government/statistics/ndns-results-from-years-9-to-11-2016-to-2017-and-2018-to-2019 (accessed 30 Nov2021).

33 Rolls BJ, Rolls ET, Rowe EA, Sweeney K. Sensory specific satiety in man. *Physiol Behav* 1981; **27**: 137–142.

34 Department of Health and Social Care. Alcohol consumption: advice on low risk drinking. 2016.https://www.gov.uk/government/publications/alcohol-consumption-advice-on-low-risk-drinking (accessed 27 May2021).

35 Poppitt SD, Keogh GF, Prentice AM, Williams DEM, Sonnemans HMW, Valk EEJ *et al.* Long-term effects of ad libitum low-fat, high-carbohydrate diets on body weight and serum lipids in overweight subjects with metabolic syndrome. *Am J Clin Nutr* 2002; **75**: 11–20.

36 Skivington K, Matthews L, Simpson SA, Craig P, Baird J, Blazeby JM *et al.* A new framework for developing and evaluating complex interventions: update of Medical Research Council guidance. *BMJ* 2021; **374**: n2061.

37 Michie S, van Stralen MM, West R. The behaviour change wheel: A new method for characterising and designing behaviour change interventions. *Implement Sci* 2011; **6**: 42.

38 Cane J, O'Connor D, Michie S. Validation of the theoretical domains framework for use in behaviour change and implementation research. *Implement Sci* 2012; **7**: 37.

39 Morrow A, Walker K, Calder-MacPhee N, Ozakinci G. The active ingredients of physical activity and / or dietary workplace-based interventions to achieve weight loss in overweight and obese healthcare staff: a systematic review. *J Behav Med* 2022; **45**: 331–349.

40 Michie S, Abraham C, Whittington C, Mcateer J, Gupta S. Effective Techniques in Healthy Eating and Physical Activity Interventions: A Meta-Regression. *Health psychology : official journal of the Division of Health Psychology, American Psychological Association* 2009; **28**: 690–701.

41 Samdal GB, Eide GE, Barth T, Williams G, Meland E. Effective behaviour change techniques for physical activity and healthy eating in overweight and obese adults; systematic review and meta-regression analyses. *Int J Behav Nutr Phys Act* 2017; **14**. doi:10.1186/s12966-017-0494-y.

42 Jensen MD, Ryan DH, Apovian CM, Ard JD, Comuzzie AG, Donato KA *et al.* 2013 AHA/ACC/TOS Guideline for the Management of Overweight and Obesity in Adults. *Circulation* 2014; **129**: S102–S138.

43 Buckell J, Mei XW, Clarke P, Aveyard P, Jebb SA. Weight loss interventions on health-related quality of life in those with moderate to severe obesity: Findings from an individual patient data meta-analysis of randomized trials. *Obesity Reviews* 2021; **22**: e13317.

For peer review only

44 Ross R, Neeland IJ, Yamashita S, Shai I, Seidell J, Magni P *et al.* Waist circumference as a vital sign in clinical practice: a Consensus Statement from the IAS and ICCR Working Group on Visceral Obesity. *Nat Rev Endocrinol* 2020; **16**: 177–189.

45 Flint A, Raben A, Blundell JE, Astrup A. Reproducibility, power and validity of visual analogue scales in assessment of appetite sensations in single test meal studies. *Int J Obes Relat Metab Disord* 2000; **24**: 38–48.

46 Powell C, Carson BP, Dowd KP, Donnelly AE. Simultaneous validation of five activity monitors for use in adult populations. *Scandinavian Journal of Medicine & Science in Sports* 2017; **27**: 1881–1892.

47 Lee PH, Macfarlane DJ, Lam TH, Stewart SM. Validity of the International Physical Activity Questionnaire Short Form (IPAQ-SF): a systematic review. *Int J Behav Nutr Phys Act* 2011; **8**: 115.

48 Meh K, Jurak G, Sorić M, Rocha P, Sember V. Validity and Reliability of IPAQ-SF and GPAQ for Assessing Sedentary Behaviour in Adults in the European Union: A Systematic Review and Meta-Analysis. *Int J Environ Res Public Health* 2021; **18**: 4602.

49 Sember V, Meh K, Sorić M, Starc G, Rocha P, Jurak G. Validity and Reliability of International Physical Activity Questionnaires for Adults across EU Countries: Systematic Review and Meta Analysis. *Int J Environ Res Public Health* 2020; **17**: E7161.

50 Craig CL, Marshall AL, Sjöström M, Bauman AE, Booth ML, Ainsworth BE *et al.* International Physical Activity Questionnaire: 12-Country Reliability and Validity. *Medicine & Science in Sports & Exercise* 2003; **35**: 1381–1395.

51 Buysse DJ, Reynolds CF, Monk TH, Berman SR, Kupfer DJ. The Pittsburgh Sleep Quality Index: a new instrument for psychiatric practice and research. *Psychiatry Res* 1989; **28**: 193–213.

52 Beriault K, Carpentier AC, Gagnon C, Ménard J, Baillargeon J-P, Ardilouze J-L *et al.* Reproducibility of the 6-minute walk test in obese adults. *Int J Sports Med* 2009; **30**: 725–727.

53 ATS Committee on Proficiency Standards for Clinical Pulmonary Function Laboratories. ATS statement: guidelines for the six-minute walk test. *Am J Respir Crit Care Med* 2002; **166**: 111–117.

54 Klukowska AM, Staartjes VE, Vandertop WP, Schröder ML. Five-Repetition Sit-to-Stand Test Performance in Healthy Individuals: Reference Values and Predictors From 2 Prospective Cohorts. *Neurospine* 2021; **18**: 760–769.

55 EuroQol. EQ-5D-3L – EQ-5D. https://euroqol.org/eq-5d-instruments/eq-5d-3l-about/ (accessed 15 Jan2022).

For peer review only

56 Kolotkin RL, Crosby RD. Psychometric evaluation of the impact of weight on quality of life-lite questionnaire (IWQOL-lite) in a community sample. *Qual Life Res* 2002; **11**: 157–171.

57 Tennant R, Hiller L, Fishwick R, Platt S, Joseph S, Weich S *et al.* The Warwick-Edinburgh Mental Well-being Scale (WEMWBS): development and UK validation. *Health Qual Life Outcomes* 2007; **5**: 63.

58 Kroenke K, Spitzer RL, Williams JBW. The PHQ-9. *J Gen Intern Med* 2001; **16**: 606–613.

59 Spitzer RL, Kroenke K, Williams JBW, Löwe B. A brief measure for assessing generalized anxiety disorder: the GAD-7. *Arch Intern Med* 2006; **166**: 1092–1097.

60 Lowe MR, Butryn ML, Didie ER, Annunziato RA, Thomas JG, Crerand CE *et al.* The Power of Food Scale. A new measure of the psychological influence of the food environment. *Appetite* 2009; **53**: 114–118.

61 Dalton M, Finlayson G, Hill A, Blundell J. Preliminary validation and principal components analysis of the Control of Eating Questionnaire (CoEQ) for the experience of food craving. *Eur J Clin Nutr* 2015; **69**: 1313–1317.

62 Willmott TJ, Pang B, Rundle-Thiele S. Capability, opportunity, and motivation: an across contexts empirical examination of the COM-B model. *BMC Public Health* 2021; **21**: 1014.

63 Gearhardt AN, Schulte EM. Is Food Addictive? A Review of the Science. *Annu Rev Nutr* 2021; **41**: 387–410.

64 David IA, Krutman L, Fernández-Santaella MC, Andrade JR, Andrade EB, Oliveira L *et al.* Appetitive drives for ultra-processed food products and the ability of text warnings to counteract consumption predispositions. *Public Health Nutrition* 2018; **21**: 543–557.

65 Batterham RL, ffytche DH, Rosenthal JM, Zelaya FO, Barker GJ, Withers DJ *et al.* PYY modulation of cortical and hypothalamic brain areas predicts feeding behaviour in humans. *Nature* 2007; **450**: 106–109.

66 Wingrove J, Makaronidis J, Prados F, Kanber B, Yiannakas MC, Magee C *et al.* Aberrant olfactory network functional connectivity in people with olfactory dysfunction following COVID-19 infection: an exploratory, observational study. *EClinicalMedicine* 2023; **58**: 101883.

67 Zhang H, Schneider T, Wheeler-Kingshott CA, Alexander DC. NODDI: Practical in vivo neurite orientation dispersion and density imaging of the human brain. *NeuroImage* 2012; **61**: 1000–1016.

68 Monteverdi A, Palesi F, Costa A, Vitali P, Pichiecchio A, Cotta Ramusino M *et al.* Subject-specific features of excitation/inhibition profiles in neurodegenerative diseases. *Frontiers in Aging Neuroscience* 2022; **14**.https://www.frontiersin.org/articles/10.3389/fnagi.2022.868342 (accessed 15 Aug2023).

For peer review only

69 Solanky BS, John NA, DeAngelis F, Stutters J, Prados F, Schneider T *et al.* NAA is a Marker of Disability in Secondary-Progressive MS: A Proton MR Spectroscopic Imaging Study. *AJNR Am J Neuroradiol* 2020; **41**: 2209–2218.

70 Feldotto B, Eppler JM, Jimenez-Romero C, Bignamini C, Gutierrez CE, Albanese U *et al.* Deploying and Optimizing Embodied Simulations of Large-Scale Spiking Neural Networks on HPC Infrastructure. *Front Neuroinform* 2022; **16**: 884180.

71 Williamson DA, Anton SD, Han H, Champagne CM, Allen R, LeBlanc E *et al.* Adherence is a multi-dimensional construct in the POUNDS LOST trial. *J Behav Med* 2010; **33**: 35–46.

72 Simpson E, Bradley J, Poliakov I, Jackson D, Olivier P, Adamson AJ *et al.* Iterative Development of an Online Dietary Recall Tool: INTAKE24. *Nutrients* 2017; **9**: E118.

73 Bradley J, Simpson E, Poliakov I, Matthews JNS, Olivier P, Adamson AJ *et al.* Comparison of INTAKE24 (an Online 24-h Dietary Recall Tool) with Interviewer-Led 24-h Recall in 11-24 Year-Old. *Nutrients* 2016; **8**: E358.

74 Foster E, Lee C, Imamura F, Hollidge SE, Westgate KL, Venables MC *et al.* Validity and reliability of an online self-report 24-h dietary recall method (Intake24): a doubly labelled water study and repeated-measures analysis. *J Nutr Sci* 2019; **8**: e29.

75 Food Standards Agency. Comparison study: INTAKE24 vs Interviewer led recall Final report. 2014https://intake24.co.uk/assets/papers/Intake24-Comparison-report.pdf.

76 Dicken SJ, Batterham RL, Brown A. Nutrients or processing? An analysis of food and drink items from the UK National Diet and Nutrition Survey based on nutrient content, the NOVA classification, and front of package traffic light labelling. *medRxiv* 2023; : 2023.04.24.23289024.

77 Bingham SA, Welch AA, McTaggart A, Mulligan AA, Runswick SA, Luben R *et al.* Nutritional methods in the European Prospective Investigation of Cancer in Norfolk. *Public Health Nutr* 2001; **4**: 847–858.

78 McKeown NM, Day NE, Welch AA, Runswick SA, Luben RN, Mulligan AA *et al.* Use of biological markers to validate self-reported dietary intake in a random sample of the European Prospective Investigation into Cancer United Kingdom Norfolk cohort. *Am J Clin Nutr* 2001; **74**: 188–196.

79 Bingham SA, Gill C, Welch A, Cassidy A, Runswick SA, Oakes S *et al.* Validation of dietary assessment methods in the UK arm of EPIC using weighed records, and 24-hour urinary nitrogen and potassium and serum vitamin C and carotenoids as biomarkers. *Int J Epidemiol* 1997; **26 Suppl 1**: S137-151.

80 Freedman LS, Midthune D, Arab L, Prentice RL, Subar AF, Willett W *et al.* Combining a Food Frequency Questionnaire With 24-Hour Recalls to Increase the Precision of Estimation of Usual Dietary Intakes-Evidence From the Validation Studies Pooling Project. *Am J Epidemiol* 2018; **187**: 2227–2232.

For peer review only

81 Barnard ND, Alwarith J, Rembert E, Brandon L, Nguyen M, Goergen A *et al.* A Mediterranean Diet and Low-Fat Vegan Diet to Improve Body Weight and Cardiometabolic Risk Factors: A Randomized, Cross-over Trial. *Journal of the American Nutrition Association* 2022; **41**: 127–139.

82 Fuller NR, Lau NS, Denyer G, Simpson AE, Gerofi J, Wu M *et al.* A 12-week, randomised, controlled trial to examine the acceptability of the Korean diet and its effectiveness on weight and metabolic parameters in an Australian overweight and obese population. *Obesity Research & Clinical Practice* 2012; **6**: e71–e83.

83 Sacks FM, Carey VJ, Anderson CAM, Miller ER, Copeland T, Charleston J *et al.* Effects of High vs Low Glycemic Index of Dietary Carbohydrate on Cardiovascular Disease Risk Factors and Insulin Sensitivity. *JAMA* 2014; **312**: 2531–2541.

84 Appel LJ, Sacks FM, Carey VJ, Obarzanek E, Swain JF, Miller ER *et al.* Effects of Protein, Monounsaturated Fat, and Carbohydrate Intake on Blood Pressure and Serum LipidsResults of the OmniHeart Randomized Trial. *JAMA* 2005; **294**: 2455–2464.

85 Ginsberg HN, Kris-Etherton P, Dennis B, Elmer PJ, Ershow A, Lefevre M *et al.* Effects of Reducing Dietary Saturated Fatty Acids on Plasma Lipids and Lipoproteins in Healthy Subjects. *Arteriosclerosis, Thrombosis, and Vascular Biology* 1998; **18**: 441–449.

86 Berglund L, Lefevre M, Ginsberg HN, Kris-Etherton PM, Elmer PJ, Stewart PW *et al.* Comparison of monounsaturated fat with carbohydrates as a replacement for saturated fat in subjects with a high metabolic risk profile: studies in the fasting and postprandial states. *The American Journal of Clinical Nutrition* 2007; **86**: 1611–1620.

87 Palinkas LA, Horwitz SM, Green CA, Wisdom JP, Duan N, Hoagwood K. Purposeful sampling for qualitative data collection and analysis in mixed method implementation research. *Adm Policy Ment Health* 2015; **42**: 533–544.

88 Dwan K, Li T, Altman DG, Elbourne D. CONSORT 2010 statement: extension to randomised crossover trials. *BMJ* 2019; **366**: l4378.

89 White IR, Horton NJ, Carpenter J, Pocock SJ. Strategy for intention to treat analysis in randomised trials with missing outcome data. *BMJ* 2011; **342**: d40.

90 Jakobsen JC, Gluud C, Wetterslev J, Winkel P. When and how should multiple imputation be used for handling missing data in randomised clinical trials – a practical guide with flowcharts. *BMC Med Res Methodol* 2017; **17**: 162.

91 Hernán MA, Robins JM. Per-Protocol Analyses of Pragmatic Trials. *New England Journal of Medicine* 2017; **377**: 1391–1398.

92 Gale NK, Heath G, Cameron E, Rashid S, Redwood S. Using the framework method for the analysis of qualitative data in multi-disciplinary health research. *BMC Medical Research Methodology* 2013; **13**: 117.

For peer review only

93 Timlin D, McCormack JM, Simpson EE. Using the COM-B model to identify barriers and facilitators towards adoption of a diet associated with cognitive function (MIND diet). *Public Health Nutr*; **24**: 1657–1670.

94 Dicken SJ, Batterham RL. Ultra-processed food: a global problem requiring a global solution. *Lancet Diabetes Endocrinol* 2022; **10**: 691–694.

For peer review only

/ScholarOne/conversions/6443719738255878786/51349623_File000004_1259532859.docx

PARTICIPANT INFORMATION SHEET

Sponsor Protocol Number: 151582
IRAS ID number: 311525

Study Title: UPDATE Trial: A study comparing the health effects of two diets following UK dietary guidance in people living with overweight or obesity

Name of Chief Investigator: Professor Rachel L. Batterham

Study team:
Professor Claudia Wheeler-Kingshott, Dr Adrian Brown, Dr Abigail Fisher, Dr Chris van Tulleken, Dr Janine Makaronidis, Dr Jed Wingrove, Tapiwa Ruwona, Samuel Dicken

Thank you for taking the time to read this participant information sheet (PIS). We would like to invite you to take part in our study investigating the benefit of eating a healthy, balanced diet as recommended by UK governments. The results from this study will be used to help inform UK dietary guidance and food policy. Before you decide if you would like to take part, it is important that you understand why this research is being done and what it will involve. Please take the time to decide whether or not you wish to take part. A member of the study team will call you in the next week to go through this information sheet with you and answer any questions you may have. You may also wish to discuss this information with your GP, your family or friends before deciding if you would like to take part.

Ask us if there is anything that is not clear. Take time to decide whether or not you wish to take part. We appreciate that this information sheet may not answer all of your questions, so please do not hesitate to contact a member of the study team on the telephone numbers given at the end of this information sheet if you would like to discuss any aspect of the study further.

Thank you for taking the time to read this information.

For peer review only

For peer review only - http://bmjopen.bmj.com/site/about/guidelines.xhtml

Study summary:

- UK governments recommend a healthy, balanced diet, which involves eating a variety of foods in the right proportions, such as five daily portions of fruit and vegetables, and limiting foods high in saturated fat, added sugar and salt.
- We want to see if the benefit of a healthy, balanced diet depends on the types of food processing in the diet.
- Staff at University College London Hospitals (UCLH) living with overweight or obesity will be invited to take part.
- There are two parts to this study, the first part is a diet intervention, the second part is behavioural support intervention to eat a healthier diet and be more active.
- In the first part, you will be given two diets in a random order, for 8 weeks each.
- Both diets are healthy and balanced, but differ in the types of food processing.
- After the first 8-week diet, you will return to your normal diet for 4 weeks, before we give you the second 8-week diet.
- We will not tell you which diet you will be given first.
- You will be given all your meals, snacks and drinks for both 8-week diets for free.
- After the second 8-week diet, we will then support you to eat a healthier diet and be more physically active for 6 months.
- We will collect data from you before you start both diets, at 4 weeks and 8 weeks into each diet, and then at the end of the 6-month support program.
- We will collect data about your health, including blood pressure, body composition, blood samples, physical activity and fitness, sleep and quality of life.
- The risk associated with taking part in the study is low, and no adverse events are expected from taking part.
- You can leave the study at any time, without giving a reason.
- You will be involved in the study for 49 weeks.

view only

For peer review only - http://bmjopen.bmj.com/site/about/guidelines.xhtml

1. What is the purpose of the study?

Eating a healthy, balanced diet is important for our health. A healthy, balanced diet involves eating a variety of foods in the right proportions. In the UK, the Eatwell Guide gives us recommendations on what we should eat more of, and what we should eat less of. Unhealthy diets contain lots of foods high in saturated fat, added sugar and salt, which increases our risk of developing obesity, cardiovascular disease and type 2 diabetes. Advice for a healthy, balanced diet includes consuming five portions of fruit and vegetables per day and eating more high fibre foods. Following the healthy, balanced diet advice given in the Eatwell Guide can reduce our risk of poor health.

However, there are other parts of our diet that are not covered in the Eatwell Guide that may also be important for health. For example, research suggests that some types of food processing might influence our health, but these types of food processing are not included within the recommendations.

We do not know if the benefit of following the healthy diet advice in the Eatwell Guide depends on the types of food processing in our diets. It is important that we find out if these types of food processing matter, in order to give people the best advice possible. It also means that the government and other health organisations may need to change the regulations around the food we eat.

To answer this, we will compare the effects of two healthy, balanced diets following the advice in the UK Eatwell Guide, but each based on a different type of food processing. Participants will receive the first diet for 8 weeks, return to their normal diet for 4 weeks, and then receive the second diet for a further 8 weeks. They will have 6 months of support to help improve their diet and be more physically active. The results from this study will be used to help inform UK dietary guidance and food policy.

The study will also contribute towards the award of a PhD at University College London (UCL).

There are two parts to this study:

1. We will study if the benefit from eating a healthy, balanced diet depends on the types of food processing in the diet. We will do this by providing people with two diets that follow the Eatwell Guide, but containing foods with different types of processing, for 8 weeks each.
2. We will then study whether people are able to switch from their usual unhealthy diet to a healthy, balanced diet, and the benefits of doing so. We will do this by providing people with 6 months of personal support. We will also look at what helps people to maintain a healthy diet, and what makes it difficult. We will also support people to be more physically active.

2. Why have I been invited?

You have been invited to take part because you are a member of staff at UCL Hospitals (UCLH) living with overweight or obesity, and you have provided interest in participating in the study by contacting our research team.

For peer review only - http://bmjopen.bmj.com/site/about/guidelines.xhtml

However, you will be unable to take part if you have dietary restrictions that limit you from consuming the provided diets, such as having food allergies, or being vegan or vegetarian. You also cannot take part if you are pregnant and/or breastfeeding, or living with diabetes.

3. Do I have to take part?

No, the decision to take part is completely up to you. No one should force you into taking part. A member of the study team will contact you by telephone or email within a week of you receiving this invitation letter to see if you are still interested in taking part. We will describe the study and go through this information sheet. You can discuss any further questions you may have about the study with them. If you choose not to take part, that is completely fine. This will not affect any care you receive from the NHS, nor affect your role at UCLH. If you agree to take part, we will then ask you to sign a consent form. Even after signing the consent form, you are still able to withdraw from the study at any time without giving a reason. This would not affect the standard of any care you receive.

4. What will happen to me if I decide to take part?

If you decide to take part, you will be asked to sign two copies of the trial consent form. You will be given a copy to take home and keep. An appointment at a time and day convenient for you will be arranged for you to attend a screening visit. Signing the consent form however will not automatically enrol you onto the trial. Your eligibility to participate will depend on the data obtained during the screening visit. The research team will notify you on the outcome of this.

If you decide to take part, you will first be given one of the two diets for 8 weeks. You will then return to your normal diet for 4 weeks, before being given the second diet for another 8 weeks. After the second diet, you will be given 6 months of support to help you switch to and maintain a healthy, balanced diet and be more physically active.

For part 1 of this study, we will see if the benefit of consuming a healthy diet depends on the types of food processing in the diet. You will be given two diets in a random order, determined by a computer:

A. A diet following UK dietary guidance for 8 weeks containing foods based on one type of food processing
B. A diet following UK dietary guidance for 8 weeks containing foods based on another type of food processing

You will be given the diets in a random order, meaning that you could have diet A first then diet B, or diet B first then diet A. We will not tell you which diet you are on. Before the first diet, we will give you healthy eating advice from the Eatwell Guide, which the diets are based on. We will also explain to you about how to report how much of the food provided you have eaten. After the first 8-week diet, we will ask you to return to your normal diet you were eating before for 4 weeks. We will not provide your food or drink during this period. You will then receive the second diet for 8 weeks. After the second 8-week diet, we will stop providing you with food and drink, and you will then need to obtain your own food and drink. We will collect data on blood pressure, body composition, physical activity and fitness, questions regarding quality of life, mental health and

For peer review only - http://bmjopen.bmj.com/site/about/guidelines.xhtml

wellbeing, and blood samples at the start of each diet and at 4 and 8 weeks into each diet. We will work with you to find a suitable period of time to conduct part 1 of the study around any holidays or commitments you have.

The aim of part 1 is to collect important scientific data to see how food processing impacts on our health, rather than to assess specific foods. Therefore, we will provide you with all meals, snacks and drinks for the two 8-week diets, which have been designed with limited flexibility. You will not need to purchase any food or drink during this time. These will be set menus, featuring typical foods recommended in the Eatwell Guide that you might buy from a shop or make yourself at home. The meals and snacks on each menu will be varied across the week and include fruit, vegetables, beef, chicken, pork, fish, bread, potatoes, pasta, rice, noodles, oats, yoghurts, flapjacks, nuts, tea and coffee. We will not provide any alcohol. The diets will be delivered to your home at a time convenient for you or delivered to a safe space every few days. The meals will require minimal preparation, being ready to eat, or needing a few minutes in the microwave. Foods provided will need to be stored in either a fridge, freezer, or cupboard. We will provide a menu guide and instructions on how to prepare your food. Once prepared, you can then eat as little or as much of the food we give you as you like. We will give you kitchen scales and containers to prepare and store food for each day. You will have weekly telephone contact with the research team at a time that is convenient for you to help with any issues with the diets.

To provide you with your food for the diets, you will need to consent to and give your address and contact details to the food suppliers. This is explained in more detail in section 21.

Part 2 of the study will begin after you finish the second 8-week diet. A behavioural scientist will chat with you to create a personal plan to help you to eat a healthier diet and be more physically active. This support will last for 6 months, with ongoing monthly telephone/video calls with the research team to discuss your progress and support you. At the end of the 6 months, we will collect data on blood pressure, body composition, physical activity and fitness, questions regarding quality of life, mental health and wellbeing, and blood samples. We will also invite you to a one-to-one interview to chat about what aspects helped you to eat healthily and be more active, and what aspects made it difficult.

5. How many sessions do I need to attend?

You will be required to attend nine sessions:

- Screening visit

Part 1 of the study:

- Before the start of the first diet
- 4 weeks into the first diet
- 8 weeks into the first diet
- Before the start of the second diet
- 4 weeks into the second diet
- 8 weeks into the second diet

Part 2 of the study:

For peer review only - http://bmjopen.bmj.com/site/about/guidelines.xhtml

- At the start of the behavioural support program (this is a 60-90 minute remote telephone/video call)
- At the end (6 months) of the behavioural support program

Each visit will last 60-90 minutes except for screening, which will last around 30-40 minutes. These sessions will take place at UCLH or at the nearby Centre for Obesity Research (COR). We will conduct the visits at a time that suits you, that fits in with your work schedule and other commitments. If you are selected for MRI scans, this will be an extra 60 minutes at visits 2, 4, 5 and 7.

After all data is collected from visit 9 (the 6-month follow-up for the behavioural support), nothing more will be expected or asked of you, and you will have completed the study.

6. What data will be collected about me?

Screening

At the screening assessment, we will collect information to check that you are eligible and that it is safe for you to take part in the study. This will include:

- Checking that you do not have a medical diagnosis of an eating disorder.
- Checking your medical history to make sure that you do not have any medical conditions which might put you at risk during the study (such as Coeliac disease).
- Checking your current diet to see the amounts of different types of food processing in your diet.
- Checking that you do not have any food allergies or dietary restrictions that make it unsafe or not possible for you to eat the diets that we will provide you in part 1 of the study.
- If you are female, checking that you are not pregnant. We will ask you to take a urine pregnancy test. Urine taken for the pregnancy test will be disposed of following testing and not kept for any reason.

If you are not eligible, your information will be destroyed after 25 years.

During the study

The tests and data we will need to collect will include:

- Blood pressure and heart rate. We will measure blood pressure using a cuff that is placed on your upper arm. Heart rate will be measured with a small clip placed on your finger, called an oximeter.
- Weight and body composition, such as muscle mass and fat mass. You will stand on a scanner that uses an electric current to assess the amount of fat and muscle you have, for 30 seconds. Scales will be used to measure your weight, a stadiometer to measure your height, and a tape measure to measure your waist circumference.
- Blood samples before and after a meal. We will give you a standard meal to eat at our research centre. A 15ml tablespoon sized blood sample will be collected before and 15 minutes and 30 minutes after the meal, which will be performed by a trained healthcare professional. We will measure changes in the hormones that regulate your appetite, as well as markers of metabolism. Blood samples will be taken at five visits: before the start of the

For peer review only - http://bmjopen.bmj.com/site/about/guidelines.xhtml

first and second diet, 8 weeks into the first and second diet, and at the end (6 months) of the behavioural support program. You will need to fast overnight for 12 hours before these five visits.

- How active you are during the day, and how much sleep you are getting. We will ask you to wear a monitor for 7 days to track your movement and sleep. The monitor is worn on your dominant hip or wrist for one week, from waking in the morning until going to bed at night. The monitor can be removed when taking a bath. We need a minimum of four days with at least 10 hours of daily wear time to collect useful data. The activity tracker records only basic movements, and does not record any identifiable information.
- How fit you are. We will ask you to complete three fitness tests. This will include squeezing a handgrip as hard as you can (handgrip strength test), measuring how far you can walk in 6 minutes (six-minute walk test), and testing your ability to stand from a seated position (sit-to-stand test).
- Questionnaires. We will ask you to fill in questionnaires about your physical activity, sleep, mental health, quality of life and eating behaviour, and what made it easier or harder for you to stick with your diet. The questionnaires will be provided to you in an electronic format, and can be completed at the study visit or remotely.
- Dietary information. To keep track of what you are eating, we will ask you to recall what you have eaten in a 24-hour period for two days, and ask you to fill in a questionnaire that asks how frequently you have eaten certain foods. We will explain how to complete these. We will ask you to also take some photos of your prepared meals for a couple of days during the 4- and 8-week follow-up visits in part 1 of the study. If it helps, we can also provide you with a diet diary to keep track of what you eat during each diet.
- A 60-90 minute interview at the end of the study to discuss the 6-month support program. A trained behavioural scientist will chat with you about the aspects of your personal, social and work life that helped you to stick with a healthy diet, and what aspects of your life made it difficult. This interview will be audio recorded and then converted into text. Some of the things you say may be quoted verbatim, but this will be anonymised before being published, so it will not be possible to identify you. Interviews will take place via telephone or video call at a time convenient for the participant during the scheduled visit time. Transcriptions will be anonymised following transcription, and audio recordings deleted as soon as transcriptions of the recordings have been obtained.

For some participants, we will also collect:

- Brain imaging scans. Not all participants will undergo a brain imaging scan. You may be selected to receive a brain scan if you consent, but you can opt out of this on the consent form. You will have a magnetic resonance imaging (MRI) scan on your brain. MRI does not expose you to ionising radiation. Brain imaging will be conducted before you start each diet, and at the 8-week visits for each diet. The scans will last around 60 minutes for each visit, in addition to the 60-90 minute visits. Scans will be conducted at the Institute of Neurology next to Russell Square. We can book these scans around your schedule at a separate visit if necessary. If you do not wish to undergo brain imaging if chosen, you can opt out on the consent form. We will provide you with more information about the brain scans at the screening visit. The brain is important for determining what we eat. Different areas of the brain can impact on the types of foods we eat, and evidence suggests that connections

For peer review only - http://bmjopen.bmj.com/site/about/guidelines.xhtml

between these different areas of the brain may change whilst eating certain diets. We want to learn more about how connections between different brain areas change when eating two different diets.

7. How long will I be involved in the study for?

After signing the written informed consent form, it is expected that your involvement in the study will last for around 49 weeks. Part 1 of the study will last around 24 weeks. Part 2 of the study will last around 25 weeks.

8. Study Timeline

The study timeline is outlined below.

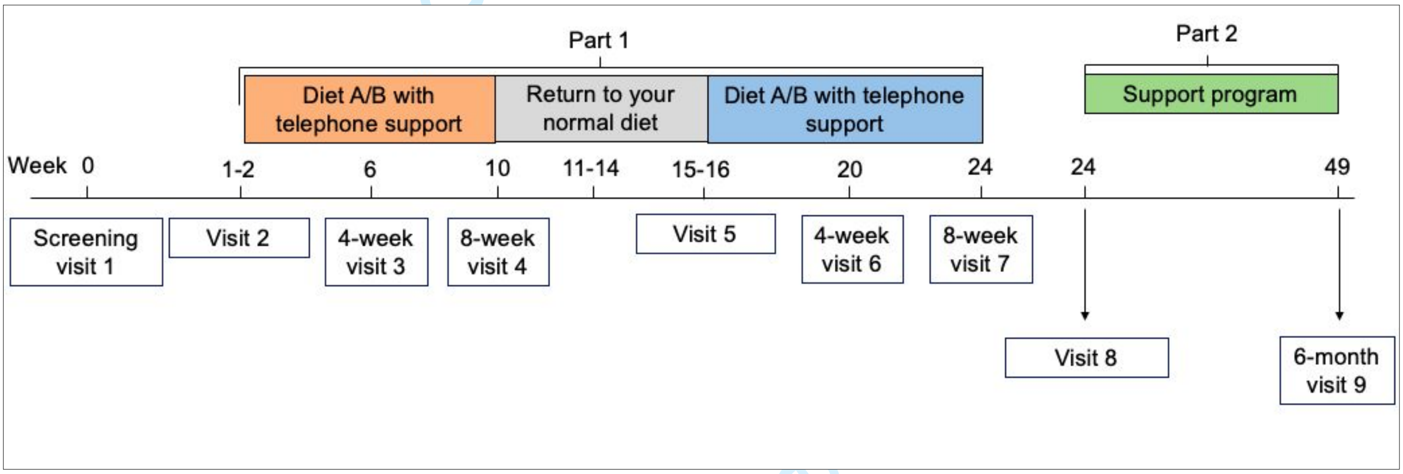

Assessment visit schedule and data collection

The table below outlines the measures collected at each visit and what will happen during the study.

		Part 1						Part 2	
		First diet			Second diet				
	Screening	Before start of diet	At 4 weeks	At 8 weeks	Before start of diet	At 4 weeks	At 8 weeks	At the start of support program	At the end of support program
**Visit Number:**	1	2	3	4	5	6	7	8	9
**Week:**	0	1-2	6	10	15-16	20	24	24	49
Informed Consent for Screening	Y								
Medical history	Y	Y	Y	Y	Y	Y	Y		Y

For peer review only - http://bmjopen.bmj.com/site/about/guidelines.xhtml

Heart rate and blood pressure	Y	Y	Y	Y	Y	Y	Y		Y
Urine Pregnancy Test (females only)	Y								
Body composition		Y	Y	Y	Y	Y	Y		Y
Blood samples before and after a meal: you will need to fast for 12 hours before this visit		Y		Y	Y		Y		Y
Physical activity and sleep monitor		Y		Y	Y		Y		Y
Physical Fitness Tests		Y	Y	Y	Y	Y	Y		Y
Questionnaires		Y	Y	Y	Y	Y	Y		Y
Dietary information	Y	Y	Y	Y	Y	Y	Y		Y
Brain scans (if chosen)		Y		Y	Y		Y		
Behavioural support call								Y	
One to one telephone/video call interview									Y

For peer review only

9. Where will the study take place?

The study will take place at the Centre for Obesity Research (COR) at UCL and at UCLH. If selected for brain scans, these will be at the nearby UCL Institute of Neurology. You will not need to travel to any other location at any other time.

10. What will I have to do?

If enrolled in the study, we will expect you to attend all scheduled ither in-person or remote study visits, and perform the tests and assessments as listed above, accordingly to the study protocol. Importantly, you will be asked to prepare and eat the diets provided to you in part 1, and not to consume other foods or drinks during each 8-week period. The research team will be available if you need to contact them, for example, if there are issues with adherence due to social events,

For peer review only - http://bmjopen.bmj.com/site/about/guidelines.xhtml

issues with the provided diets (e.g. a missing delivery or missing meals) or problems with dietary reporting.

11. What are the possible disadvantages and risks of taking part?

Possible disadvantages and risks are related to the study assessments, procedures and questionnaires you will have to undergo when taking part in the trial. However, these would be considered minor risks/disadvantages, as explained below. The study is generally low risk. We do not expect there to be any major risks or adverse events from taking part in the study. There are no expected risks from the screening visit.

The two 8-week diets that we will provide you in part 1:

- As part of the screening process, we will make sure that you do not have any allergens or dietary restrictions to the foods we will provide you in part 1. We will check that there are no potential concerns regarding your eating behaviour.
- We will provide you with simple instructions on how to safely prepare and store your meals, and you will not be expected to consume foods beyond their best by date or use by date.

Blood samples before and after a meal:

- Taking blood samples may occasionally cause a bruise or pain. All samples will be obtained by a trained healthcare professional.

Physical fitness tests:

- The physical fitness measures are not expected to result in a risk of injury. Trained researchers will provide full instructions and supervision for the tests.

Questionnaires:

- You might find some of the questions about your mental health and quality of life upsetting. However, you can stop the questionnaires at any time, or decline to answer specific questions, without having to give a reason or without affecting your rights. If you do feel upset and you feel you would benefit from some emotional support, you can contact the Samaritans charity (https://www.samaritans.org) on 116 123, their helpline is open 24 hours per day. You can also contact MIND (https://www.mind.org.uk), or the CALM (Campaign against living miserably) charity, who have a helpline open from 5pm to midnight every day on 0800 58 58 58 or a web chat (https://www.thecalmzone.net).

Support program:

- In order to support you with eating a healthy diet and being more physically active, the behavioural scientist may discuss aspects of your personal, social and work life that may be upsetting. The aim of the discussion is to help you, such that there is a positive impact on your health. As with the questionnaires, you can stop the discussions at any time, or decline to answer specific questions, without having to give a reason or without affecting your rights.

For peer review only - http://bmjopen.bmj.com/site/about/guidelines.xhtml

Brain scans:

- If you consent and are selected for brain imaging assessment (unless you choose to opt out on the consent form), you will undergo an MRI scan. MRI does not expose you to ionising radiation. But, the noise from the scanner and small space can cause stress and discomfort.
- Our researchers will check that you are suitable to undergo MRI brain scanning during screening. We will also check for any reasons why you cannot undergo an MRI scan, which will be confirmed prior to every scan.

Unexpected findings

In the unlikely event that we notice an abnormal finding in some of the measurements we take (including blood tests, heart rate, blood pressure and brain scans), we will inform you and your GP, who will discuss the findings and any further action needed with you.

Will I experience any side-effects?

We do not expect there to be any side effects from the diet interventions, from the support program, or from the tests involved.

The diets provided contain typical foods in UK diets and are obtained from supermarkets or made in a professional catering facility.

It is important that if you do feel any unusual symptoms, regardless of whether you think they are linked to the study, that you tell a member of the study team.

12. What are the possible benefits of taking part?

It is likely that you will experience a benefit from the 6-month support program as you will be supported to improve your diet and be more physically active. It is not possible to guarantee any specific benefit from part 1 of the study, where we provide you with two diets.

The knowledge gained from this study will provide further information into whether a healthy diet depends on how processed it is. Results from this study can be used to inform changes to the current recommendations for dietary guidelines. A greater understanding of the long-term risks of diets can better inform healthcare professionals on helping people living with obesity.

13. How many participants will be in the study?

We will recruit 55 members of staff at UCLH living with overweight or obesity. The study will run for approximately 18 months in total.

14. What happens when the trial stops?

We will only provide your food and drink for the two 8-week diets in part 1 of the study. We will not be able to provide behavioural support after the end of the study.

For peer review only - http://bmjopen.bmj.com/site/about/guidelines.xhtml

At the end of the trial, we will analyse the data and publish the results in medical journals, and present results at scientific conferences. If you wish, a copy of the reports on the study findings can be requested from the Chief Investigator, Professor Batterham, at the address given in section 19 of this information sheet. Should the study be stopped prematurely for any reason, we will tell you why.

15. Will my taking part in the study be kept confidential?

Yes. We will follow ethical and legal practices and all information about you will be handled in confidence. The details are outlined in the following section 19.

16. Will I be paid to take part in the study?

You will receive the two 8-week diets for free in part 1 of the study. This means you will not need to purchase any food or drink for 16 weeks. We will not directly pay you for any aspects of the study.

We do not foresee any travel costs, as all study measurements will be at UCLH or the adjacent COR, so you will not have to make any extra journeys or travel anywhere other than to your usual appointment location. If you do need to travel specifically for the study visits, any reasonable travel costs incurred to attend the study visit will be reimbursed on production of valid receipts. All the food we provide will be delivered to your home every few days, so you do not need to travel to collect your food. We will work with you to ensure food deliveries, meetings or assessments align with your work and other commitments.

None of the study team are being paid beyond their normal salary to conduct the research, nor are any clinicians being paid for recruiting individuals into the study.

17. What if relevant new information becomes available?

Sometimes during a clinical trial, new information about the intervention becomes available. If this happens, we will tell you about it and discuss with you whether you want to or should continue your participation in this study. If you decide not to carry on, we will make arrangements for your care to continue. If you decide to continue in the study, you might be asked to sign an updated consent form. Also, on receiving new information, we might consider it to be in your best interest to withdraw from the study. If so, we will explain the reasons and arrange for your care to continue.

18. What will happen if I don't want to carry on with the study?

Your participation in the trial is entirely voluntary and you will be free to withdraw from the study at any time and without giving a reason. A decision to withdraw will not affect the current or future care you receive. If you withdraw from the study, we will destroy all your identifiable information, but we will need to use and analyse the pseudonymised data collected up to the point of your withdrawal. You will be provided with a withdrawal form where you will be able to express your preference. In any case, pseudonymised (coded) information collected up to the point of your

For peer review only - http://bmjopen.bmj.com/site/about/guidelines.xhtml

withdrawal will still be used in the analysis. Any stored blood or tissue samples that can still be identified as yours will be destroyed if you wish.

19. What if there is a problem?

If you have any problem during the study, or would like to discuss any aspect of the study, you can contact any of the research investigators. You can find their contact details on the last page of this information sheet. Every care will be taken in the course of this clinical trial.

However, in the unlikely event that you are injured by taking part, compensation may be available through the University College London (UCL) insurance scheme. If you suspect that the injury is the result of the Sponsor's (University College London) or the hospital's negligence then you may be able to claim compensation. After discussing with your research doctor, please make the claim in writing to the Chief Investigator for this trial:

Professor Rachel Batterham,
Centre for Obesity Research,
Rayne Building,
5 University Street,
London, WC1E 6JF.

The Chief Investigator will then pass the claim to the Sponsor's Insurers, via the Sponsor's office.

However, if you remain unhappy or have a complaint about any aspect of this study and wish to speak to someone independent of the research team/hospital, please contact the Head of Research Governance and Compliance, UCL/UCLH Joint Research Office, University College London, Gower Street, London WC1E 6BT email: research-incidents@ucl.ac.uk.

Regardless of this, if you wish to complain, or have any concerns about any aspect of the way you have been approached or treated by members of staff or about any side effects (adverse events) you may have experienced due to your participation in the clinical trial, the normal National Health Service complaints mechanisms are available to you. You could also write or get in touch with the Complaints Manager, UCL hospitals. Please quote the study IRAS number at the top of this information sheet. Please ask your study doctor if you would like more information on this. Details can also be obtained from the NHS website and the Department of Health website: http://www.dh.gov.uk. You can also contact the UCLH Patient Advise Liaison Service (PALS) via phone: 020 3447 3042 or email: uclh.pals@nhs.net. In case you feel you would benefit from some emotional support, you can contact the Samaritans charity on 116 123, their helpline is open 24 hours per day. The CALM (Campaign against living miserably) charity also have a helpline open from 5pm to midnight every day on 0800 58 58 58 or a web chat (https://www.thecalmzone.net).

20. How will we use information about you?

Data Protection Information

We will need to use information from you and your medical records for this research project. This information will include your contact details [name, initials, telephone number(s), email address, address, NHS/hospital number], demographics [age, gender, marital status, ethnicity, education,

For peer review only - http://bmjopen.bmj.com/site/about/guidelines.xhtml

occupation]), which will be held by UCL. People will use this information to do the research or to check your records to make sure that the research is being done properly. People who do not need to know who you are will not be able to see your name or contact details. Your data will have a code number instead. We will keep all information about you safe and secure. Once we have finished the study, we will keep some of the data so we can check the results. We will write our reports in a way that no-one can work out that you took part in the study.

UCL is the sponsor for this study based in the United Kingdom. The sponsor is the organisation responsible for ensuring that the study is carried out correctly. UCL will act as the data controller for this study. This means that they are responsible for looking after your information and using it properly. UCL will keep this information about you for 25 years after the study has finished. The UCL Data Protection Office provides oversight of UCL activities involving the processing of personal data and can be contacted at data-protection@ucl.ac.uk. Further information on how UCL uses your information can be found on our general research privacy notice here https://www.ucl.ac.uk/legal-services/privacy.

In order to conduct the study, staff outside your normal care will need access to your medical records. You will need to give permission to the study team to access your medical records, and for long-term storage in an anonymised form. All of your personal information will be kept strictly confidential. The study will legally comply with the Data Protection Act, 2018. Your patient records and study information will be stored behind a card-secure door, with access only by members of the research team. Any data that leaves the study sites (UCLH and UCL) will be anonymised. Your name and other identifiable information will not be recorded on records or samples. Your initials and trial identification number will be used on records or samples.

When you agree to take part in a research study, the information about your health and care may be provided to researchers running other research studies in this organisation and in other organisations. These organisations may be universities, NHS organisations or companies involved in health and care research in this country or abroad. Your information will only be used by organisations and researchers to conduct research in accordance with the UK Policy Framework for Health and Social Care Research.

We collect personal data directly from you, or your GP and/or hospital team if needed, for the purposes of carrying out this research study. We use your name, NHS number and contact details to contact you about the research study and make sure that relevant information about the study is recorded for your care, and to oversee the quality of the study. We only share your personal data in limited circumstances, with your GP, as set out in this information sheet, and with individuals from regulatory organisations where they need to look at your medical and research records to check the accuracy of the research study. The only people in UCL who will have access to information that identifies you will be people who need to contact you as part of the study or audit the data collection process. The people who analyse the study data will not be able to identify you and will not be able to find out your name, NHS number or contact details. We will keep all information about you safe and secure. Once we have finished the study, we will keep some of the data so we can check the results. The final study reports will contain anonymised data only and will not contain any identifiable data or personal information.

For peer review only - http://bmjopen.bmj.com/site/about/guidelines.xhtml

Your personal data will be processed for the purposes outlined in this information sheet. The legal basis to process your personal data will be:

- to undertake the research study, on the basis of performance of a task carried out in the public interest and processing your special categories of personal data (e.g., health information) for research purposes.
- where required to make disclosures to your GP further to the results of any study test/questionnaire, on the basis of performance of a task carried out in the public interest, compliance with a legal obligation and processing your special categories of personal data (e.g., health information) for public health purposes.
- to contact you about participating in further research studies, only where you have provided consent on the consent form. You have the right to withdraw your consent to be contacted about future studies at any time, using the details set out at the end of this section.

To deliver the 8-week diets to you, the food suppliers will need your name, contact details and address. We will not share your personal details with the food suppliers. For one of the diets, we will control a secure, password-protected online food delivery account that only the research team can log into. We will ask you to add your name, phone number and address to the account delivery details for the purposes of delivering the diet to you. The supermarket will handle your data according to their privacy policy. We will ask you to add your details to an account for a second supermarket, to be used only if there are issues with delivery from the first supermarket. It is not possible to determine that you are involved in the study from this. When your time on this 8-week diet ends, we will delete your details from the account. For the other diet, you will need to give your name, address and contact details directly to the food supplier. We will give you the details needed to contact them, if you are eligible and decide to participate. You will need to agree to this on the consent form.

Your personal data will be processed only for so long as it is required for the research project. We will pseudonymise the personal data as soon as registered on the study database and endeavour to minimise the processing of personal data, wherever possible, to safeguard your privacy. In the event of any future loss of capacity to make decisions relating to your ongoing participation in this study, you will automatically be withdrawn from the study. The information already collected up to that point, may continue to be used confidentially in connection with this study and future research, if applicable.

In line with the regulations, at the end of the study your data will be securely archived for a minimum of 25 years.

Under data protection law, you have individual rights in relation to the personal data we hold about you. For the purposes of research where such individual rights would seriously impair research outcomes, such rights may be limited. In fact, as we need to manage your records in specific ways for the research to be reliable, we will not be able to let you see or change the data we hold about you. If you would like to exercise a right under data protection law or find out more about how we use your information, please contact data-protection@ucl.ac.uk.

Personal contact details will be removed at the end of the study if you decide to opt out of receiving a results summary.

For peer review only - http://bmjopen.bmj.com/site/about/guidelines.xhtml

We may need to request information about you from your medical records via your GP if we are unable to obtain this from yourself. This information may include age, gender, ethnicity, job occupation, work pattern, educational level, marital status, medication intake, alcohol consumption, smoking habits, family history of obesity and possible related diseases. Any data sent from your GP to the researchers will be transferred using encrypted NHS email systems that are secure for sending confidential information.

21. What are your choices about how your information is used?

- You can stop taking part in the study at any time, without giving a reason, but we will keep information about you that we already have.
- We need to manage your records in specific ways for the research to be reliable. This means that we won't be able to let you see or change the data we hold about you.
- If you agree to take part in this study, you will have the option to take part in future research using your data saved from this study. This is explained in section 24.

22. Where can you find out more about how your information used?

You can find out more about how we use your information:

- at www.hra.nhs.uk/information-about-patients/
- our leaflet available from www.hra.nhs.uk/patientdataandresearch
- by asking one of the research team
- by sending an email to data-protection@ucl.ac.uk, or
- by ringing us on 0203 108 8764

If you remain unsatisfied, you may wish to contact the Information Commissioner's Office (ICO) (https://ico.org.uk/concerns/handling/).

23. Will my GP be informed of my involvement?

With your permission, your GP will be notified that you are taking part in this study. If the study investigators became concerned about your well-being or about the implications of what you tell us for someone else's well-being we would need to inform your GP or other professionals. We would, of course, discuss this with you first. In addition, should your blood test results show any abnormality, we will let your GP know, who will be able to make necessary arrangements for your best care. You will be asked to agree to this when signing the consent form.

24. What will happen to my blood samples?

As part of this trial, we will collect new blood samples from you during the face-to-face visits, as detailed above. These blood samples will be stored and analysed at UCLH and the Centre for Obesity Research, University College London (UCL). Samples will be anonymised, which means they will not be identifiable. The samples will be identified only by your study number and will not be directly marked with your name. The samples will only be used for the study described above, however, with your consent, any remaining blood samples you provided for the study may be

For peer review only - http://bmjopen.bmj.com/site/about/guidelines.xhtml

retained after the end of the study for future research. Only researchers involved in this study will have access to the blood samples. You can choose how you would like your samples to be used after the study ends in the consent form. We can transfer your samples to the Obesity Research Biobank Syndicate (ORBiS) run by COR, where they will be stored in a secure database and used for further research, or they can be safely disposed of. Your samples will be disposed of in accordance with the Human Tissue Act 2004. If you choose to withdraw from the study, any stored blood or tissue samples that can still be identified as yours will be destroyed if you wish.

25. What will happen to the results of the trial?

The results of the study will be available after it finishes and will usually be published in a medical journal or presented at a scientific conference. The data will be anonymised and none of the participants involved in this study will be identified in any report or publication. We will be happy to make the results of the study available to you. You can request a lay summary of the study results by ticking the corresponding box in the consent form.

26. Who is conducting the trial?

The study is being run by the UCL Centre for Obesity Research who care for patients living with obesity.

27. Who is organising and funding the trial?

This research study is being organised by the Centre for Obesity Research, University College London (UCL). The Chief Investigator is Professor Rachel L. Batterham (Consultant Obesity Physician, Diabetologist, and Endocrinologist) who has vast experience in clinical research studies. This study is being sponsored by University College London (UCL) and being funded by the National Institute for Health Research (NIHR) and Rosetrees trust.

28. How have patients and the public been involved in this trial?

In designing and planning this study, we have taken into account the opinions of UCLH staff following a focus group discussion, who have reviewed and given feedback on the study design and this Information Sheet.

29. Who has reviewed the trial?

It is important that when we carry out research, we make sure the study design is as good as possible to reduce the risk of bias and poor-quality research. All research in the NHS is looked at by independent group of people, called a Research Ethics Committee, to protect your interests. This study has been reviewed and given favourable opinion by the Yorkshire & The Humber - Sheffield Research Ethics Committee. Academics from UCL, National Institutes of Health in the US and academic peer-reviewers have also evaluated and reviewed the study design.

30. Further information and contact details

For peer review only - http://bmjopen.bmj.com/site/about/guidelines.xhtml

If you require any further information or have any concerns while taking part in the study, please contact one of the following people:

Samuel Dicken Phone: 07415690476 Email: samuel.dicken.20@ucl.ac.uk

Fred Jassil Email: friedrich.jassil.13@ucl.ac.uk

If you decide you would like to take part, then please read and sign the consent form. You will be given a copy of this information sheet and the consent form to keep. A copy of the consent form will be filed in your patient notes, one will be filed with the study records and one may be sent to the Research Sponsor.

You can have more time to think this over if you are at all unsure.

Thank you for taking the time to read this information sheet and for considering this study.

For peer review only

For peer review only - http://bmjopen.bmj.com/site/about/guidelines.xhtml

CONSENT FORM

Centre for Obesity Research
Division of Medicine
University College London
Rayne Building
5 University Street
London WC1E 6JF
Telephone: 020 7679 0788

Email: samuel.dicken.20@ucl.ac.uk
r.batterham@ucl.ac.uk

For peer review only

UPDATE trial: A study comparing the health effects of two diets following UK dietary guidance in people living with overweight or obesity.

Name of Chief Investigator: Professor Rachel L Batterham **Participant screening number:**

Sponsor Protocol Number: 151582
IRAS ID number: 311525

Thank you for considering taking part in this project. If you have any questions arising from the information sheet or explanation already given to you, please ask the research team before you decide whether to join, contact details are on the top right-hand side of this page. This consent form is to confirm that you are happy to undergo the screening assessments to participate in the study, and if eligible, to participate in the study. For some elements, you must circle an option (e.g., consent/do not consent). You must circle one option and initial the box to confirm your selection. Leaving any boxes not initialled will result in the consent form being incomplete and not valid.

Please initial box

1	I confirm that I have read the participant information sheet (Version 1.1, dated 12.12.2022), which explains the benefits and risks of taking part in the study.	
2	I confirm that I have been provided with sufficient information and opportunity to ask any questions about the study and have had these answered satisfactorily.	
3	I confirm that I have been informed about any risk that may result from taking part in this study and that I have had sufficient time to consider whether or not I want to take part.	
4	I confirm that I understand the requirements for participating in the study, and that I am able to attend all relevant study sessions and procedures as outlined in the participant information sheet (Version 1.1, dated 12.12.2022).	
5	I understand that my participation in this study is voluntary. It is my right to withdraw from the study at any time, with no penalties. I do not need to give a reason for doing so. This will not affect my standard care or my legal rights.	
6	I understand that relevant sections of my medical notes and data collected during the study may be looked at by individuals from the sponsor of the trial (University College	

For peer review only - http://bmjopen.bmj.com/site/about/guidelines.xhtml

	London) and responsible persons authorised by the sponsor, from regulatory authorities or from the NHS Trust, where it is relevant to my taking part in this research. I give permission for these individuals to have access to my records. I understand that such information will be handled appropriately and confidentially.	
7	I understand that the UCL research team will access parts of my medical record and my data collected in the study. I give permission for this access.	
8	I understand that my name, contact details and address need to be used for the purposes of delivering the diets to my home at a time that is convenient for me, as outlined in the participant information sheet (Version 1.1, dated 12.12.2022). The UCL research team will not share my details with the food suppliers. I agree that I will provide my contact details and address to the food suppliers to deliver the diets.	
9	I give permission for a researcher to contact me for the purposes of offering support during the interventions, ensuring the diet deliveries are received, in collecting study samples and to remind me to complete the study measurements.	
10	I give permission for my anonymised data to be used in future publications related to this study and to be stored securely for up to 25 years after the end of the study.	
11	I acknowledge that UCL will handle my personal data for the purposes of this research study and I have been provided with information about how UCL handles personal data, as described in the participant information sheet (Version 1.1, dated 12.12.2022).	
12	I understand that my GP will be informed that I am participating in this study.	
13	I consent that in the event that I lose the capacity to make decisions relating to my ongoing participation in this study, I will automatically be withdrawn from the study. The information already collected up to that point may continue to be used confidentially in connection with this study and future research, if applicable, as described in the participant information sheet (Version 1.1, dated 12.12.2022).	
14	I agree to the one-to-one interview at the 6-month follow-up to be recorded for data analysis purposes, and I understand that some of the things I say may be quoted verbatim in publications without me being directly identified.	
15	I consent / do not consent (please circle) to being potentially chosen to have the MRI brain scans as described in the participant information sheet (Version 1.1, dated 12.12.2022).	
16	I give permission for my blood samples to be stored and analysed at the University sites.	
17	At the end of the study, I consent / do not consent (please circle) to having my blood samples stored in ORBiS for future research as explained in the participant information sheet (Version 1.1, dated 12.12.2022). If I do not agree, I understand that my samples will be disposed of according to Human Tissue Act 2004.	
18	I consent / do not consent (please circle) for my personal information to be retained and used to contact me for the purpose of participating in future research.	

For peer review only - http://bmjopen.bmj.com/site/about/guidelines.xhtml

19	I would / would not (please circle) like to receive a summary of the results.	
20	I voluntarily consent and agree to undergoing a screening assessment to determine my eligibility for the study, and if eligible, participating in the above study.	

---------------------------------- ----------------------------- ----------------------

Name of participant Signature Date

I confirm that I have explained the study to the above participant and have answered questions honestly and fully.

---------------------------------- ---------------------------- ----------------------

Name of investigator taking consent Signature Date

Comments or concerns during the study

If you have any comments or concerns, you may discuss these with the investigators. If you wish to go further and complain about any aspect of the way you have been approached or treated during the course of the study, you should write or get in touch with the Complaints Manager, UCL Hospitals. Please quote the study number on the front page of this consent form.

When completed: 1 (original) for participant; 1 (original) for researcher site file; 1 to be kept in medical notes.

For peer review only - http://bmjopen.bmj.com/site/about/guidelines.xhtml

Supplementary Materials: Contraception list

Female participants

Females of childbearing potential are eligible to participate if they agree to use a highly effective contraception method for the duration of the trial and until 6 weeks after treatment discontinuation. Women are considered of childbearing potential following menarche and until becoming post-menopausal unless permanently sterile. Women are considered permanently sterile if they have had documented hysterectomy, bilateral salpingectomy or bilateral oophorectomy. Postmenopausal state is defined as no menses for 12 months without any medical cause. A high Follicle Stimulating Hormone (FSH) level in the postmenopausal range may be used to confirm a postmenopausal state in women not using hormonal contraception or Hormonal Replacement Therapy (HRT). However in the absence of 12 months of amenorrhoea, a single FSH measurement is insufficient.

Highly effective contraceptive methods include:

- Combine (oestrogen and progesterone containing) hormonal contraception associated with inhibition of ovulation:
  - Oral
  - Intravaginal
  - Transdermal
- Progesterone-only hormonal contraception associated with inhibition of ovulation:
  - Oral
  - Injectable
  - Implantable
- Intrauterine device.
- Intrauterine hormone-releasing system.
- Bilateral tubal occlusion.
- Vasectomised partner.
- True sexual abstinence (refraining from sexual intercourse – only acceptable when this is in line with the preferred and usual lifestyle of the subject).

Male participants

Male participants with partners of childbearing potential should use barrier methods of contraception for the duration of the trial until 6 weeks after treatment discontinuation.

For peer review only

Supplementary Materials: Schedule of Assessments

			First diet intervention				Second diet intervention		Behavioural support program	
	Screening	Baseline Assessment first diet	4-week follow-up first diet	8-week follow-up first diet	4-week washout period	Baseline Assessment second diet	4 -week follow-up second diet	8-week follow-up second diet	Behavioural support counselling	6-month follow-up
**Visit No:**	**1**	**2**	**3**	**4**		**5**	**6**	**7**	**8**	**9**
**Week**	**0**	**1-2**	**6**	**10**	**11-14**	**15-16**	**20**	**24**	**24**	**49**
Informed Consent	✓[a]									
Concomitant Medication review	✓	✓	✓	✓		✓	✓	✓		✓
Adverse Events review		✓	✓	✓		✓	✓	✓		✓
Urine Pregnancy Test	✓									
Socio-demographics	✓									
Medical History and Co-morbidities	✓	✓	✓	✓		✓	✓	✓		✓
Vital Signs	✓	✓	✓	✓		✓	✓	✓		✓
Randomisation[b] for diet order		✓								
Height	✓									
Weight	✓	✓	✓	✓		✓	✓	✓		✓
Waist circumference		✓	✓	✓		✓	✓	✓		✓
BIA		✓	✓	✓		✓	✓	✓		✓
Blood sample collection		✓		✓		✓		✓		✓
Meal test		✓		✓		✓		✓		✓
Accelerometry (ActiGraph)		✓		✓		✓		✓		✓
Physical activity and sleep questionnaires		✓	✓	✓		✓	✓	✓		✓
Physical Function Assessment (6MWT, STS, HGST)		✓	✓	✓		✓	✓	✓		✓
Mental health and wellbeing Questionnaires		✓	✓	✓		✓	✓	✓		✓
Eating behaviour Questionnaires		✓	✓	✓		✓	✓	✓		✓
COM-B questionnaire		✓								✓
Behavioural support program									✓	
One to one semi-structured phone/video call interview										✓

For peer review only

Supplementary Materials: Schedule of Assessments

Diet assessment: 24-hr recall (Intake24)	✓	✓	✓	✓		✓	✓	✓		✓
Diet assessment: FFQ (EPIC-Norfolk FFQ)		✓	✓	✓		✓	✓	✓		✓
Diet assessment: IBDA			✓	✓			✓	✓		
MRI brain scans in a subset		✓		✓		✓		✓		

[a] Prior to Screening.

[b] Randomisation will be undertaken after the participant has completed the baseline diet assessment.

Abbreviations: BIA: Bioelectrical Impedance Analysis; COM-B: capability, opportunity, motivation – behaviour; FFQ: food frequency questionnaire; HGST: handgrip strength test; IBDA: image-based dietary assessment; MRI: magnetic resonance imaging; STS-test: sit-to-stand test; 6MWT: 6-minute walk test.

For peer review only

SPIRIT

STANDARD PROTOCOL ITEMS: RECOMMENDATIONS FOR INTERVENTIONAL TRIALS

SPIRIT 2013 Checklist: Recommended items to address in a clinical trial protocol and related documents*

Section/item	ItemNo	Description	
**Administrative information**			
Title	1	Descriptive title identifying the study design, population, interventions, and, if applicable, trial acronym	Y
Trial registration	2a	Trial identifier and registry name. If not yet registered, name of intended registry	Y
	2b	All items from the World Health Organization Trial Registration Data Set	
Protocol version	3	Date and version identifier	Y
Funding	4	Sources and types of financial, material, and other support	Y
Roles and responsibilities	5a	Names, affiliations, and roles of protocol contributors	Y
	5b	Name and contact information for the trial sponsor	Y
	5c	Role of study sponsor and funders, if any, in study design; collection, management, analysis, and interpretation of data; writing of the report; and the decision to submit the report for publication, including whether they will have ultimate authority over any of these activities	Y
	5d	Composition, roles, and responsibilities of the coordinating centre, steering committee, endpoint adjudication committee, data management team, and other individuals or groups overseeing the trial, if applicable (see Item 21a for data monitoring committee)	Y
**Introduction**			
Background and rationale	6a	Description of research question and justification for undertaking the trial, including summary of relevant studies (published and unpublished) examining benefits and harms for each intervention	Y

For peer review only

	6b	Explanation for choice of comparators	Y
Objectives	7	Specific objectives or hypotheses	Y
Trial design	8	Description of trial design including type of trial (eg, parallel group, crossover, factorial, single group), allocation ratio, and framework (eg, superiority, equivalence, noninferiority, exploratory)	Y
**Methods: Participants, interventions, and outcomes**			
Study setting	9	Description of study settings (eg, community clinic, academic hospital) and list of countries where data will be collected. Reference to where list of study sites can be obtained	Y
Eligibility criteria	10	Inclusion and exclusion criteria for participants. If applicable, eligibility criteria for study centres and individuals who will perform the interventions (eg, surgeons, psychotherapists)	Y
Interventions	11a	Interventions for each group with sufficient detail to allow replication, including how and when they will be administered	Y
	11b	Criteria for discontinuing or modifying allocated interventions for a given trial participant (eg, drug dose change in response to harms, participant request, or improving/worsening disease)	Y
	11c	Strategies to improve adherence to intervention protocols, and any procedures for monitoring adherence (eg, drug tablet return, laboratory tests)	Y
	11d	Relevant concomitant care and interventions that are permitted or prohibited during the trial	Y
Outcomes	12	Primary, secondary, and other outcomes, including the specific measurement variable (eg, systolic blood pressure), analysis metric (eg, change from baseline, final value, time to event), method of aggregation (eg, median, proportion), and time point for each outcome. Explanation of the clinical relevance of chosen efficacy and harm outcomes is strongly recommended	Y
Participant timeline	13	Time schedule of enrolment, interventions (including any run-ins and washouts), assessments, and visits for participants. A schematic diagram is highly recommended (see Figure)	Y

For peer review only - http://bmjopen.bmj.com/site/about/guidelines.xhtml

Sample size	14	Estimated number of participants needed to achieve study objectives and how it was determined, including clinical and statistical assumptions supporting any sample size calculations	Y
Recruitment	15	Strategies for achieving adequate participant enrolment to reach target sample size	Y

Methods: Assignment of interventions (for controlled trials)

Allocation:

Sequence generation	16a	Method of generating the allocation sequence (eg, computer-generated random numbers), and list of any factors for stratification. To reduce predictability of a random sequence, details of any planned restriction (eg, blocking) should be provided in a separate document that is unavailable to those who enrol participants or assign interventions	Y
Allocation concealment mechanism	16b	Mechanism of implementing the allocation sequence (eg, central telephone; sequentially numbered, opaque, sealed envelopes), describing any steps to conceal the sequence until interventions are assigned	Y
Implementation	16c	Who will generate the allocation sequence, who will enrol participants, and who will assign participants to interventions	Y
Blinding (masking)	17a	Who will be blinded after assignment to interventions (eg, trial participants, care providers, outcome assessors, data analysts), and how	Y
	17b	If blinded, circumstances under which unblinding is permissible, and procedure for revealing a participant's allocated intervention during the trial	Y

Methods: Data collection, management, and analysis

Data collection methods	18a	Plans for assessment and collection of outcome, baseline, and other trial data, including any related processes to promote data quality (eg, duplicate measurements, training of assessors) and a description of study instruments (eg, questionnaires, laboratory tests) along with their reliability and validity, if known. Reference to where data collection forms can be found, if not in the protocol	Y

For peer review only - http://bmjopen.bmj.com/site/about/guidelines.xhtml

	18b	Plans to promote participant retention and complete follow-up, including list of any outcome data to be collected for participants who discontinue or deviate from intervention protocols	Y
Data management	19	Plans for data entry, coding, security, and storage, including any related processes to promote data quality (eg, double data entry; range checks for data values). Reference to where details of data management procedures can be found, if not in the protocol	Y
Statistical methods	20a	Statistical methods for analysing primary and secondary outcomes. Reference to where other details of the statistical analysis plan can be found, if not in the protocol	Y
	20b	Methods for any additional analyses (eg, subgroup and adjusted analyses)	Y
	20c	Definition of analysis population relating to protocol non-adherence (eg, as randomised analysis), and any statistical methods to handle missing data (eg, multiple imputation)	Y
**Methods: Monitoring**			
Data monitoring	21a	Composition of data monitoring committee (DMC); summary of its role and reporting structure; statement of whether it is independent from the sponsor and competing interests; and reference to where further details about its charter can be found, if not in the protocol. Alternatively, an explanation of why a DMC is not needed	Y
	21b	Description of any interim analyses and stopping guidelines, including who will have access to these interim results and make the final decision to terminate the trial	Y
Harms	22	Plans for collecting, assessing, reporting, and managing solicited and spontaneously reported adverse events and other unintended effects of trial interventions or trial conduct	Y
Auditing	23	Frequency and procedures for auditing trial conduct, if any, and whether the process will be independent from investigators and the sponsor	Y
**Ethics and dissemination**			

Research ethics approval	24	Plans for seeking research ethics committee/institutional review board (REC/IRB) approval	Y
Protocol amendments	25	Plans for communicating important protocol modifications (eg, changes to eligibility criteria, outcomes, analyses) to relevant parties (eg, investigators, REC/IRBs, trial participants, trial registries, journals, regulators)	Y
Consent or assent	26a	Who will obtain informed consent or assent from potential trial participants or authorised surrogates, and how (see Item 32)	Y
	26b	Additional consent provisions for collection and use of participant data and biological specimens in ancillary studies, if applicable	Y
Confidentiality	27	How personal information about potential and enrolled participants will be collected, shared, and maintained in order to protect confidentiality before, during, and after the trial	Y
Declaration of interests	28	Financial and other competing interests for principal investigators for the overall trial and each study site	Y
Access to data	29	Statement of who will have access to the final trial dataset, and disclosure of contractual agreements that limit such access for investigators	Y
Ancillary and post-trial care	30	Provisions, if any, for ancillary and post-trial care, and for compensation to those who suffer harm from trial participation	Y
Dissemination policy	31a	Plans for investigators and sponsor to communicate trial results to participants, healthcare professionals, the public, and other relevant groups (eg, via publication, reporting in results databases, or other data sharing arrangements), including any publication restrictions	Y
	31b	Authorship eligibility guidelines and any intended use of professional writers	Y
	31c	Plans, if any, for granting public access to the full protocol, participant-level dataset, and statistical code	Y

Appendices

For peer review only

Informed consent materials	32	Model consent form and other related documentation given to participants and authorised surrogates	Y
Biological specimens	33	Plans for collection, laboratory evaluation, and storage of biological specimens for genetic or molecular analysis in the current trial and for future use in ancillary studies, if applicable	In manuscript

*It is strongly recommended that this checklist be read in conjunction with the SPIRIT 2013 Explanation & Elaboration for important clarification on the items. Amendments to the protocol should be tracked and dated. The SPIRIT checklist is copyrighted by the SPIRIT Group under the Creative Commons "Attribution-NonCommercial-NoDerivs 3.0 Unported" license.

For peer review only

For peer review only - http://bmjopen.bmj.com/site/about/guidelines.xhtml

BMJ Open

UPDATE trial: Investigating the effects of Ultra-Processed versus minimally processed Diets following UK dietAry guidance on healTh outcomEs: a protocol for an 8-week community-based crossover randomised controlled trial in people with overweight or obesity, followed by a 6-month behavioural intervention.

Journal:	*BMJ Open*
Manuscript ID	bmjopen-2023-079027.R1
Article Type:	Protocol
Date Submitted by the Author:	27-Dec-2023

Complete List of Authors:	Dicken, Samuel; UCL, Centre for Obesity Research<br>Makaronidis, Janine; University College London, Centre for Obesity Research, Department of Medicine; University College London Hospital (UCLH), Bariatric Centre for Weight Management and Metabolic Surgery<br>van Tulleken, Chris; University College London, Division of Infection<br>Jassil, Friedrich; University College London, Division of Medicine; University College London Hospital (UCLH), Bariatric Centre for Weight Management and Metabolic Surgery<br>Hall, Kevin; National Institute of Diabetes and Digestive and Kidney Diseases, Bethesda, MD, National Institutes of Health<br>Brown, Adrian; UCL, Centre for Obesity Research, Department of Medicine; University College London Hospital (UCLH), Bariatric Centre for Weight Management and Metabolic Surgery<br>Gandini Wheeler-kingshott, Claudia; Queen Square Multiple Sclerosis Centre, UCL Institute of Neurology, Neuroinflammation; Brain MRI 3T Research Center, C. Mondino National Neurological Institute<br>Fisher, Abigail; University College London, Department of Behavioural Science and Health<br>Batterham, Rachel; UCL, Centre for Obesity Research, Department of Medicine; University College London Hospital (UCLH), Bariatric Centre for Weight Management and Metabolic Surgery

**Primary Subject Heading**:	Nutrition and metabolism
Secondary Subject Heading:	Diabetes and endocrinology, Public health, Qualitative research
Keywords:	NUTRITION & DIETETICS, Obesity, PUBLIC HEALTH, Clinical Trial

SCHOLARONE™
Manuscripts

For peer review only - http://bmjopen.bmj.com/site/about/guidelines.xhtml

UPDATE trial: Investigating the effects of **U**ltra-**P**rocessed versus minimally processed **D**iets following UK diet**A**ry guidance on heal**T**h outcom**E**s: a protocol for an 8-week community-based crossover randomised controlled trial in people with overweight or obesity, followed by a 6-month behavioural intervention.

Samuel J. Dicken[1], Janine Makaronidis[1,2,3], Chris Van Tulleken[4], Friedrich C Jassil[1,2,3], Kevin Hall[5], Adrian Brown[1,2,3], Claudia A. M. Gandini Wheeler-Kingshott[6,7,8], Abi Fisher[9], Rachel L. Batterham*[1,2,3]

1. Centre for Obesity Research, Department of Medicine, University College London (UCL), London WC1E 6JF, UK.
2. Bariatric Centre for Weight Management and Metabolic Surgery, University College London Hospital (UCLH), London NW1 2BU, UK.
3. National Institute for Health Research, Biomedical Research Centre, University College London Hospital (UCLH), London W1T 7DN, UK.
4. Division of Infection, University College London, London WC1E 6BT, UK.
5. National Institute of Diabetes and Digestive and Kidney Diseases, National Institutes of Health, US.
6. NMR Research Unit, Queen Square MS Centre, Department of Neuroinflammation, UCL Institute of Neurology, Faculty of Brain Sciences, University College London, London, United Kingdom.
7. Department of Brain and Behavioural Sciences, University of Pavia, Pavia, Italy.
8. Digital Neuroscience Center, IRCCS Mondino Foundation, Pavia, Italy.
9. Behavioural Science and Health, University College London, London, UK.

*Corresponding Author; r.batterham@ucl.ac.uk

For peer review only

Abstract

Introduction Obesity increases the risk of morbidity and mortality. A major driver has been the increased availability of ultra-processed food (UPF), now the main UK dietary energy source. The UK Eatwell Guide (EWG) provides public guidance for a healthy balanced diet, but offers no UPF guidance. Whether a healthy diet can largely consist of UPFs is unclear. No study has assessed whether the health impact of adhering to dietary guidelines depends on food processing. Furthermore, our study will assess the impact of a 6-month behavioural support programme aimed at reducing UPF intake in people with overweight/obesity and high UPF intakes.

Methods and analysis UPDATE is a 2x2 crossover randomised controlled trial with a 6-month behavioural intervention. Fifty-five adults aged ≥18, with overweight/obesity (≥25 to <40 kg/m^2), and ≥50% of habitual energy intake from UPFs will receive an 8-week UPF diet and an 8-week minimally processed food (MPF) diet delivered to their home, both following EWG recommendations, in a random order, with a 4-week washout period. All food/drink will be provided. Participants will then receive 6-months of behavioural support to reduce UPF intake. The primary outcome is the difference in weight change between UPF and MPF diets from baseline to week 8. Secondary outcomes include changes in diet, waist circumference, body composition, heart rate, blood pressure, cardiometabolic risk factors, appetite regulation, sleep quality, physical activity levels, physical function/strength, wellbeing and aspects of behaviour change/eating behaviour at 8 weeks between UPF/MPF diets, and at 6-months follow-up. Quantitative assessment of changes in brain magnetic resonance imaging functional resting-state connectivity between UPF/MPF diets, and qualitative analysis of the behavioural intervention for feasibility and acceptability will be undertaken.

Ethics and dissemination Sheffield Research Ethics Committee approved the trial (22/YH/0281). Peer-reviewed journals, conferences, PhD thesis and lay media will report results.

Trial registration number NCT05627570.

Strengths and limitations of this study

- This protocol outlines the methodology for a free-living study comparing the health effects of MPF and UPF diets following EWG dietary recommendations, and the effectiveness of a 6-month behavioural support programme to reduce UPF intake.
- Strengths of the randomised controlled trial include the long 8-week duration of each intervention diet, and provision of all food and drink to participants' homes.to provide
- Strengths of the behavioural support programme include the use of evidence-based behaviour change techniques, and qualitative and quantitative analyses using a theoretical framework.
- A subset of participants will undergo MRI brain scans.
- Limitations include the exclusion of individuals with type 2 diabetes or with dietary restrictions (e.g., vegan, vegetarian, Halal, Kosher).

For peer review only

Introduction

Obesity is a global healthcare challenge. Nearly two thirds of UK adults now live with overweight or obesity[1], increasing the risk of life-limiting disease and premature mortality[2]. The overall annual cost of obesity in the UK is estimated at £58 billion[3]. Of which, the National Health Service (NHS) spends over £6 billion/year on obesity-related health issues, which is expected to rise to nearly £10 billion/year by 2050[4].

A major driver of the obesity epidemic has been the shift in the food environment with increased availability of ultra-processed food (UPF)[5–7]. Defined by NOVA, UPFs are industrial formulations with five or more ingredients, using extracts of original foods with preservatives, flavourings and colours[8]. UPFs include breakfast cereals, sweets, packaged breads and ready meals. Over 50% of UK energy intake now comes from UPFs, displacing more traditional minimally processed food (MPF)[9]. In prospective cohort studies, higher UPF intakes are associated with increased risks of weight gain, overweight/obesity[10,11], cardiometabolic disease[12], gastrointestinal disorders, cancer[13], mental health problems[14], lower physical strength[15] and all-cause mortality[16].

The Eatwell Guide (EWG) provides the UK public with guidance for a healthy, balanced diet[4,17,18]**.** The EWG focusses on macronutrients and food groups, such as choosing foods lower in saturated fat, sugar and salt, and eating five daily portions of fruit and vegetables[18]. The EWG recommends 'reduced fat' or 'lower sugar' reformulated foods, based on European Food Safety Authority nutrition claim regulations. However, this promotes the intake of UPFs[19]. Given their improved nutritional quality from reduced saturated fat, added sugar and/or salt, reformulated UPFs are often marketed as "healthy"[20]. However, the associations between high UPF intakes and adverse health outcomes appear to independent of diet quality or diet pattern[16].

In the only randomised trial to date examining the effect of energy-matched UPF versus MPF diets, participants gained nearly 1kg body weight on the 2-week UPF diet, but lost nearly 1kg on the 2-week MPF diet, with over 500kcal/day differences in energy intake[21]. The diets were also matched for presented energy density, carbohydrate, sugar, fat, sodium and fibre content. The MPF diet also led to favourable changes in appetite-regulating gut hormones compared with the UPF diet. In epidemiological substitution modelling, replacing UPFs for MPFs leads to weight loss, whereas replacing MPFs for UPFs leads to weight gain, independent of diet pattern (by adjusting for adherence to a Mediterranean diet)[22]. However, to date, UK organisations such as the British Nutrition Foundation and the Scientific Advisory Committee on Nutrition (SACN) do not recommend the inclusion of UPF in dietary guidelines. This is due to the largely observational body of evidence regarding the negative impact of ultra-processing on health, and lack of high-quality interventional evidence[23,24].

Adopting a healthy diet requires a significant behaviour change and lifestyle modification which can be challenging for many individuals. The prevalence of obesity in the UK are also present amongst UK healthcare professionals (HCPs)[1,25]. Many HCPs consume an unhealthy diet[26], attributed to the high availability and accessibility of ready-to-eat

For peer review only

unhealthy food and lack of healthier choices in the workplace especially during night-shifts, stress-driven eating, long working hours, and staff shortages limiting the ability to take breaks[27]. It is therefore important to support individuals to reduce their UPF intake, and in the process, identifying barriers and facilitators to develop effective strategies for long-term behaviour change.

Objectives and hypotheses

To date, no studies have assessed whether the health impact of following dietary guidelines is dependent on food processing, nor whether providing behavioural support to UK adults living with overweight/obesity can help to reduce their UPF intake.

Therefore, this study aims to compare the health effects between MPF and UPF diets that follow the EWG recommendations, and the feasibility and acceptability of a behavioural support programme to reduce UPF intake and be more physically active.

The primary objective is to compare weight change between MPF and UPF diets following EWG recommendations (Table 1). Secondary objectives are to compare changes in cardiometabolic, behavioural, mental and hormonal outcomes between MPF and UPF diets following EWG recommendations and explore the feasibility and acceptability of the behavioural support intervention.

We hypothesise that there will be a difference in the change in weight and other health measures between the two diets; whereby consuming an *ad libitum* MPF diet complying with the EWG will result in weight loss and favourable changes in cardiometabolic, behavioural, mental and hormonal outcomes, whereas consuming an *ad libitum* UPF diet complying with the EWG will result in no change in weight or cardiometabolic, behavioural, mental and hormonal outcomes. If the hypotheses are supported, the results would indicate important health implications of the foods that make up the majority of the energy intake of the UK adult population and the differential impact of UPF and MPF, regardless of following current NHS dietary guidance. The results have the potential to substantially impact on dietary guidelines and the dietary management of obesity worldwide, supporting the development of regulations around the marketing and labelling of UPFs, and increasing knowledge and public awareness of the health consequences of UPFs[28]. The results are directly relevant for UK public health food policy as the interventions are based on the UK EWG. Given the similarities between UK and other national dietary guidelines, there will be important implications for guidelines worldwide as well.

Table 1. UPDATE trial design based on PICOT

Population	Adults (UCLH staff) living with overweight or obesity, and with ≥50% of habitual energy intake from UPFs.
Intervention	A minimally processed diet complying with UK EWG guidance.
Comparator	An ultra-processed diet complying with UK EWG guidance.
Outcome (primary)	Mean difference in percent weight change between intervention and comparator at 8 weeks.
Timepoints	Primary outcome assessed from baseline to 8 weeks follow-up on each diet.

EWG, Eatwell Guide; UCLH, University College London Hospitals; UK, United Kingdom; UPF, ultra-processed food.

Methods and analysis

Trial design and study setting

UPDATE is a single-site, community-based, 2x2 crossover randomised controlled trial (RCT) followed by a six-month behavioural intervention, conducted in the UK by the Centre for Obesity Research, Division of Medicine, University College London (UCL) and University College London Hospitals (UCLH). The protocol was designed according to Standard Protocol Items: Recommendations for Interventional Trials (SPIRIT) guidelines (https://www.spirit-statement.org)[29].

In summary, 55 adults aged 18 or older, living with overweight/obesity (between 25 kg/m^2 and 40 kg/m^2), with 50% or more of habitual energy intake from UPFs will be recruited. Participants will receive an 8-week, minimum 80% UPF diet and an 8-week, minimum 80% MPF diet, both following UK EWG macronutrient recommendations, in a random order, with a 4-week washout period. All food and drink will be provided. All participants will then receive a 6-month behavioural support programme to reduce UPF (Figure 1).

The trial is funded by the National Institute for Health and Care Research UCLH Biomedical Research Council and Rosetrees Trust, and UCL/UCLH are the Sponsors. The Sponsor and funders were not involved in the design and conduct of the trial.

Figure 1. Flow chart of UPDATE trial.
AE, adverse events; BIA, bio-impedance analysis; BP, blood pressure; HR, heart rate; MRI, magnetic resonance imaging; RCT, randomised controlled trial; UCLH, University College London Hospitals; WC, waist circumference.

Methods: Participants, interventions, and outcomes

The trial opened to recruitment on 13th January 2023, the first individual was screened on 13th March 2023, and the first randomisation on 4th April 2023. The planned end date is 31st March 2025.

Recruitment

Potential participants will be identified through advertising at UCLH/UCL (e.g. websites, Trust email, posters, internal communications) and on social media (Twitter).

For peer review only

Interested individuals will receive a Participant information sheet (PIS) (Supplementary Material 1), and be offered a phone call with the research team. Researchers will then explain the screening procedure and the aims, methods, anticipated benefits and potential hazards of the trial, and invite individuals to attend an in-person visit to undergo screening. Written informed consent will be sought a minimum of 24 hours after receiving the PIS, at the in-person visit (see Supplementary Material 2 for the approved consent form). Participants can withdraw at any time, without giving a reason.

Eligibility criteria

Written informed consent will be obtained prior to commencing screening based on the inclusion and exclusion criteria, and before any research-associated measurement is taken (Table 2).

Table 2. Eligibility criteria

**Inclusion criteria**	**Exclusion criteria**
Staff at UCLH	Contraindication for dietary intervention
Adults aged ≥18 years	Participation in another clinical intervention trial
BMI ≥ 25 kg/m^2 (living with overweight or obesity)	Concomitant recent usage of medications that cause weight gain or weight loss
Weight stable (≤5 % variation in body weight over preceding 3 months)	Cardiometabolic comorbidities (e.g. diabetes, on insulin)
Have a habitual dietary intake consisting of ≥50% UPF intake as % total daily energy intake	Coeliac disease
Able to read and write in English	Inflammatory bowel disease
Medically safe to participate in a dietary intervention programme.	A diagnosed eating disorder
Willing and able to give written informed consent.	Planning a weight management programme in the next 3 months
Able to attend the relevant in person and remote sessions	Any diagnosed food allergy, or other allergies which limit the ability to adhere to the intervention diet
Able to comply with the study protocol (including dietary recommendations for each intervention and reporting adherence)	Dietary restrictions (e.g., vegan, vegetarian, Halal or kosher) which limit the ability to adhere to the interventions
Females of childbearing potential and males agree to use an effective method of contraception from the time consent is signed until the end of the intervention period and final follow-up assessment. Effective methods of contraception acceptable for UPDATE are outlined in the Supplementary Material 3	BMI >40 kg/m^2 or basal metabolic rate ≥2300/kcal/day (to ensure intervention diets are at least 300kcal/day greater than maintenance energy needs, based on excess energy intakes reported in Hall et al.[21])
Females of childbearing potential must be on highly effective contraception and have a negative pregnancy test	Females who are pregnant, breast-feeding, or intends to become pregnant
	A history of drug or alcohol abuse
	Any other factor making the participant unsuitable in the view of investigator

For peer review only

Participants will complete two non-consecutive 24-hour recalls at screening. If they meet all inclusion criteria and do not meet any exclusion criteria, they will complete a further two non-consecutive 24-hour recalls at baseline to confirm a habitual dietary intake of ≥50% UPF. The mean of all four recalls will be used to determine final habitual UPF intake. After which, they will be considered eligible.

Interventions

Eligible participants will receive in a random order (1): an 8-week MPF diet (at least 80% MPF (NOVA group 1), <20% UPF), and (2): an 8-week UPF diet (at least 80% UPF, <20% MPF), both following EWG recommendations. During the 4-week washout between diets, participants will return to their habitual diet to minimise carryover effects.

Participants will be given all meals, snacks and drinks for each 8-week intervention to maximise adherence, ensure internal validity and minimise dropout[30,31]. Catering companies and supermarkets will deliver diets to participants' homes, two times per week. Deliveries will be scheduled according to participants' convenience.

Both diets will follow EWG recommendations, choosing foods with green or amber front of package (FoP) traffic lights for total fat, saturated fat, sugar and salt over foods with red traffic lights, consuming 5 portions per day of fruit and vegetables, and eating a variety of foods in the right proportions[18].

Diets will be matched for and aim to follow government recommended intakes for a 2000kcal/day diet[4,18] (Table 3). Provided diets will be scaled up to 4000kcal/day, to ensure participants are not energy restricted and energy intake is *ad libitum.*

Table 3. Eatwell Guide dietary recommendations[4,18]

**Component**	**Guidance**
Total fat	35% of provided energy intake or below, 78g or less/2000kcal
Saturated fat	10% of provided energy intake or below, 24g or less/2000kcal
Salt	Less than 6g per day. Intake in line with current UK average intakes[32], aiming for below 6g per day
Carbohydrate	Around 50% of provided energy intake, 267g/2000kcal
Total sugars	Less than 90g/2000kcal, less than 18% of total energy
Protein	Around 15% of provided energy intake, 45g/2000kcal
Fibre	At least 18g/2000kcal
Fruit and vegetables	Five portions per day

UK, United Kingdom

The menus will be designed to be representative of UK diets, by identifying the most commonly consumed foods in the UK National Diet and Nutrition Survey[33]. Practical and logistical aspects including price, best-before dates, storage requirements, and accessibility will be factored into the design. Diets will be matched where possible, with MPF/UPF versions

For peer review only

of the same meals (e.g., Caesar salad, salmon and potatoes, porridge/breakfast cereal). UPF items are typically foods obtained from supermarkets, whereas MPFs are freshly made culinary preparations. The menu will vary across the week to prevent participant boredom and sensory-specific satiety[34], but repeating each week to allow consistency and reduce burden. Alcohol will not be provided. Participants will be asked to keep alcohol consumption within government guidelines (≤14 units per week)[35]. Participants will be educated on the EWG, but further lifestyle guidance will not be given. Menu guides will be provided with instructions and pictures to prepare each meal. As in previous *ad libitum* feeding trials investigating weight change[36], participants will be asked to consume as much or as little of the provided diets as they desire, but they will not be asked to actively reduce their energy intake.

Participants will receive weekly calls the research team, including dietitians, to discuss any issues with the diets and to promote adherence. Participants will have a food diary to track adherence and to record any food consumed off diet. The 4- and 8-week diet assessments will also be used for monitoring adherence. Participants will be encouraged to report any deviations from the provided diets during weekly calls and in the food diary, and to be as honest as possible, with no repercussions. Previous studies indicate high adherence when all food is provided and delivered to participant homes, where eating foods provided is more of a challenge than eating foods off the diet[30]. Minor modifications to the intervention that do not alter the overall design will be acceptable for enabling adherence. For example, adding herbs or spices (without sugar/salt) to taste, or providing alternative items to facilitate food preparation if participants do not have access to a microwave (e.g. swapping a microwaveable meal for a ready-to-eat meal).

Behavioural intervention

The study is powered for the crossover RCT, rather than for the behavioural support intervention. Therefore, in line with the Medical Research Council (MRC) guidance for developing complex interventions[37], the aim of this secondary aspect of the study is to explore the feasibility and acceptability of the behavioural support intervention to reduce UPF (primary target behaviour) and increase physical activity (secondary target). Full details of the intervention development and content will be provided in a future publication focussed on the behavioural support intervention. The exit interview guide is provided in Supplementary Material 4. However, in brief, the intervention was developed following the steps outlined in the Behaviour Change Wheel framework for intervention development[38], which incorporates the "Capability, Opportunity, Motivation" model for understanding behaviour (COM-B), augmented with Theoretical Domains Framework (TDF) for utilising behaviour change theory[37,39]. Qualitative studies of barriers and facilitators for dietary change in healthcare workers were extensively reviewed, with findings then mapped onto the TDF, and behaviour change techniques consistently associated with successful diet and physical activity change drawn from meta-analyses of RCTs (e.g.[40–42]). The intervention content combines monthly video/telephone behavioural support calls with print/online resources, based on TDF domains. Some examples of TDF domains (linked to the intervention components) are Knowledge (information on what UPFs are and how to recognise them), Environment (mapping of food outlets local to participant's workplace to for quickly accessible MPF options), Beliefs about Consequences (information about links between UPF and health), Behavioural Regulation (support with goal-setting, action-planning, self-

For peer review only

monitoring, habit) and Social Influence (moderated online peer support sessions). The calls will be individually tailored based to participant demographics/work patterns, responses to their baseline COM-B questionnaire, baseline levels of UPF intake and reported physical activity (measures of these are all described in the outcomes section) and personal barriers and motivations to change.

Outcomes

Outcome measures will be collected at seven in-person visits. See Figure 1, and Supplementary Material 5 for the Schedule of Assessments.

The primary outcome is the mean difference in percent weight change (%WC) at 8 weeks from baseline between MPF and UPF diets. This outcome is currently being used clinically in weight management clinics and used for all NHS weight management programmes. Clinically significant weight change can occur from short-term dietary interventions and directly relates to improvements in cardiometabolic risk factors (e.g., blood pressure, blood glucose, glycated haemoglobin (HbA1c), lipids)[43], physical function and quality of life[44]. Weight is an efficient and more accurate measurement to collect than other energy balance measures, such as energy intake. The hypothesised %WC in the face of the current growing obesity pandemic would have clinically relevant impacts on halting the rate of progression of obesity prevalence and mitigating the adverse impacts of adiposity-related disease, simply through shifts in the types of processed in foods being consumed.

Secondary outcomes include changes in dietary intake, waist circumference, body composition (fat mass, fat-free mass), heart rate, blood pressure, cardiometabolic risk factors (comorbidities and blood biomarkers including liver function, lipid profile, glucose, HbA1c and c-reactive protein), fasted and fed metabolomics, appetite measures (fasted and fed appetite scores, circulating gut hormones and adipocytokines), sleep, sleep quality, physical activity levels, physical function (walking distance, leg strength, handgrip strength), mental health, quality of life, wellbeing and aspects of eating behaviour between MPF and UPF diets, and after the 6-month behavioural intervention compared with the first baseline assessment. In a subset of participants, changes in brain functional resting state connectivity between MPF and UPF diets will be assessed. Further behavioural intervention secondary outcomes include understanding the experiences of UPF versus MPF diets, changes in behaviour regulation and shopping expenditure from the first baseline assessment, and barriers and facilitators to reducing UPF intake.

Meal tests with blood samples for biomarkers, metabolomics, gut hormones and adipocytokines will be collected at five visits: baseline visits and 8-weeks follow-ups for the RCT and at 6-months follow-up for the behavioural intervention. A random subset of participants will undergo MRI scans at four visits: baseline visits and 8-weeks follow-ups for the RCT. The COM-B behaviour change questionnaire will be assessed at two visits: the first baseline visits and at 6-months follow-up.

Participant timeline

The trial duration per participant is 49 weeks (see Figure 2). The overall trial is expected to last 24 months. Recruitment commenced in March 2023, and is expected to continue until March 2024 or when 55 participants have been recruited.

Figure 2. Participant timeline
FU, follow-up.

Methods: Assignment of interventions

Randomisation

Enrolled participants will be block randomised (stratified by night-shift status, sex and ethnicity as potential treatment modifiers) to receive: (1) the MPF diet then UPF diet, or (2) UPF diet then MPF diet. Randomisation will be performed using Sealed Envelope by the research team (https://www.sealedenvelope.com).

The trial statistician, but not researchers, will be blinded to the diet assignments. To prevent bias, participants are informed that the provided interventions are healthy balanced diets made with different types of food processing. All participant communications omit the terms MPF/UPF, with diets instead being referred to generally as Diet 1/A or Diet 2/B. Unblinding procedures are not required.

Methods: Data collection, management, and analysis

Data collection methods

Sociodemographics
Age, gender, ethnicity, occupation, work pattern, educational level, marital status, medication intake, alcohol consumption, smoking habits, family history of obesity, cardiovascular disease and diabetes will be collected at screening.

Anthropometrics and body composition
Weight will be measured using an electronic scale to the nearest 0.1 kg (Tanita DC-430MAS; Tanita, Tokyo, Japan). Body composition including fat mass, fat-free mass and bone mass will be assessed using bioelectrical impedance analysis (BIA) (Tanita) at each visit. Height will be determined using a stadiometer to the nearest 0.5 cm, and BMI derived from weight and height (in kg/m^2). Waist circumference will be measured using a non-stretch tape measure at the iliac crest[45].

Vital signs
Blood pressure will be recorded in triplicate, seated, alongside heart rate with an automated sphygmomanometer and oximeter.

Mixed meal test and blood samples
Mixed meal tests will be used to assess circulating gut hormones, adipocytokines and metabolomics, given the significant differences in metabolites and appetite hormones between UPF and MPF diets in Hall et al., 2019[21]. Participants will consume 187.5ml Ensure Compact 2.4kcal (https://nutrition.abbott/uk/product/ensure-compact) after an overnight fast. Fasted blood samples taken before the test include venous glucose, HbA1c, liver function

For peer review only

tests, lipid profile and C-reactive protein, relevant for cardiometabolic health. Blood samples and subjective appetite assessments using a visual analogue score will be collected immediately before, and 15 and 30 minutes after starting the liquid meal[46]. Fasted and post-prandial circulating appetite hormones (ghrelin, peptide YY and glucagon-like-peptide-1), adipocytokines as markers of adipose tissue inflammation (leptin, resistin, adiponectin, interleukin-6 and tumour necrosis factor alpha), and metabolomics analysis of 250 metabolites using the Nightingale platform (https://nightingalehealth.com) will be collected and stored at -80°C.

Physical activity levels and sleep quality

Physical activity (PA) levels will be measured objectively using accelerometry (ActiGraph wGT3X-BT). The device has been previously used in clinical research given its practicality, non-invasiveness, and reliability and accuracy in measuring PA in free-living adults[47]. Data recorded includes energy expenditure, metabolic equivalents and sleep activity. The short-form International Physical Activity Questionnaire (IPAQ-SF) will be used to subjectively assess light, moderate and vigorous PA, and time spent sitting[48–50]. The IPAQ-SF contains seven questions about PA in the past 7 days[51]. ActiGraph wGT3X-BT will also objectively measure sleep quality and quantity. The Pittsburgh Sleep Quality Index is a validated 19-item measure for determining sleep quality in the past month, to distinguish between good and poor sleepers[52].

Physical function and strength

Static muscle strength of the upper extremities will be assessed using a handgrip dynamometer (Jamar Hydraulic Hand Dynamometer, Patterson Medical). Three measures will be recorded (in kg) with each hand, alternately, whilst seated. Functional capacity will be assessed using the 6-minute walk test, a self-paced submaximal assessment, validated in people living with obesity[53]. The test will be performed according to the *"American Thoracic Society Statement: Guidelines for the Six-minute Walk Test"* protocol[54]. The pre- and post-test heart rate, total distance covered, perceived physical exertion and any physical problems will be recorded. Lower body functional capacity will be assessed using the sit-to-stand test[55]. Participants will be requested to perform five sit-to-stand repetitions as fast as possible with arms crossed over the chest. Number of repetitions and time to completion will be recorded.

Quality of life, mental health and wellbeing questionnaires

EuroQol five-dimensions three-levels (EQ-5D-3L) is a 6-item questionnaire containing a descriptive system assessing five domains: mobility, self-care, usual activities, pain/discomfort and anxiety/depression using three levels (e.g., mobility: I have no problems walking about, I have some problems walking about, or I am confined to bed) and a visual analogue scale reporting self-rated health from 0-100[56]. Impact of Weight on Quality of Life-Lite (IWQOL-Lite) is a 31-item, obesity/overweight-specific measure of health-related quality of life relating to five domains: physical function, self-esteem, sexual life, public distress, and work. Higher scores indicate a better quality of life[57]. The Warwick Edinburgh Mental Wellbeing Scale (WEMWBS) is a 14-item, positively worded, validated measure of mental wellbeing. Five responses are summed for a total score between 14-70, with higher scores indicating greater positive mental wellbeing[58]. The Patient Health Questionnaire (PHQ-9) is a 9-item validated measure to assess the severity of depression[59]. Each item is

For peer review only

rated from 0-3, for a total score out of 27. A score of 1-4 indicates minimal depression, 5-9 indicates mild, 10-14 indicates moderate, 15-19 indicates moderately severe, with 20-27 indicating severe depression. The Generalised Anxiety Disorder assessment (GAD-7) is a 7-item anxiety scale to measure the severity of generalised anxiety disorder[60]. Each item is rated from 0-3, for a total score out of 21. A score of 11-15 indicates moderately severe anxiety, with 16-21 indicating severe anxiety.

Eating behaviour questionnaires

The Power of Food (PoF) scale is a 15-item validated measure to assess the psychological impact of living in food-abundant environments[61]. The Control of Eating Questionnaire (CoEQ) is a 21-item validated measure of the severity and type of food cravings[62].

Behaviour change questionnaire

Participants will complete a questionnaire investigating the barriers and facilitators to PA and healthy eating based on COM-B[63]. The PA component contains 137 items, and the eating component contains 120 items.

Magnetic resonance imaging (MRI)

Several aspects of UPFs may influence eating behaviour, including changes to the food composition, food matrix degradation and behavioural aspects, including large portion presentation, cost, availability, shelf life, heavy marketing and attractive packaging[16]. UPFs have been suggested to have addictive-like properties[64], evoking strong emotional reactivity from visual cues[65]. Functional brain magnetic resonance imaging (MRI) has provided valuable insights into appetite regulation[66], and how obesity impacts the neurobiology of weight regulation. However, the neurobiological impact of high-UPF diets is largely unknown. A subset of participants (*n*=24) will undergo an advanced MRI brain protocol at the baseline and 8-week visits of the RCT (four visits), to assess a number of changes in brain properties: i) functional resting-state connectivity between regions implicated in eating behaviour, metabolism and swelling linked to diffuse inflammation, and their reversibility[67]; ii) microstructure brain properties extracted from diffusion weighted imaging data[68]; iii) changes in brain dynamics, based on each subject's functional and structural connectivity[69]; iv) metabolite changes, such as glutamate, N-acetyl aspartate and Inositol, through brainstem magnetic resonance spectroscopy[70]; v) regional volume changes through high-resolution structural scans[67], and vi) relaxometry properties alterations affected by inflammation[71].

Assessment of dietary adherence and intake

In line with previous trials[72], multiple approaches will be used to assess dietary intake and adherence, including 24-hour recalls, food frequency questionnaires (FFQ) and image-based dietary assessment (IBDA).

Intake24[73] is a validated, online, self-reported 24-hour recall system, based on a multiple-pass recall suitable for the general population[74,75] (https://intake24.co.uk). The web-based recall method is convenient, efficient and ensures coding consistency. Participants enter all food and drink consumed in the previous 24 hours (from waking up to going to sleep) into Intake24 on two non-consecutive days per visit period. Each recall takes roughly 12 minutes to complete[76]. The first recall at screening will be conducted with the research

For peer review only

team to ensure adequate training. Links will then be sent to participants to complete further recalls remotely. Intake24 is connected to the National Diet and Nutrition Survey Nutrient Databank to provide nutrient outputs, and has been coded into NOVA by the research team for calculating UPF/MPF intake.[77]. Intake24 includes details on the brand names of products, facilitating assignment of NOVA groups.

The European Prospective Investigation into Cancer and Nutrition (EPIC)-Norfolk FFQ is a validated, semi-quantitative measure of average dietary intake over the past year[78–80]. The FFQ contains a 130-item food list followed by detailed questions regarding the food list. For each food item, participants tick the most appropriate frequency of consumption of that item from nine options (from never or less than once per month, to 6+ per day).

Participants will complete an FFQ and two non-consecutive day 24-hour dietary recalls at baseline, and 4- and 8-weeks for both diets, and at the 6-month behavioural intervention follow-up (Figure 1)[81]. IBDA will also be completed on two non-consecutive days during weeks 4 and 8 of the RCT, whereby participants will take photos before and after their *ad libitum* meals/snacks.

Questionnaires summary

There are twelve questionnaires/surveys: PSQI, IPAQ-SF, EQ-5D-3L, IWQOL-Lite, WEMWBS, PHQ9, GAD7, PoF, CoEQ, COM-B healthy eating and PA questionnaire, Intake24 and EPIC-Norfolk FFQ. Time points for completion are detailed in Table 4 and in Supplementary Material 5.

Feasibility/acceptability and process evaluation of the behavioural intervention

Feasibility/acceptability and process evaluation will be described in full in the subsequent paper focussed on the behavioural intervention. However, the main feasibility/acceptability outcome will be percentage of intervention calls successfully delivered, as well as retention to the study. For quality control intervention calls will be recorded and coded against a checklist to ensure that target BCTs were being delivered as planned. Acceptability of the intervention content and process will be gathered in qualitative interviews. Changes in other outcomes pre and post the behavioural intervention will be explored as secondary.

Qualitative interviews

In the first behavioural intervention call, before being told that the intervention(s) focus on processing, participants will be asked to describe their experiences of each of the trial diets 1A/2B (positives and negatives, how they felt physically/mentally, barriers they faced to adherence) then probed on which diet they thought was UPF vs MPF and why. In addition, at the end of the behavioural intervention participants will be invited to take part in a one-to-one semi-structured telephone interview about their experiences of the trial overall, motivations for participation, barriers to reducing UPF and increasing MPF, and experience of the behavioural intervention specifically (whether they found materials and process acceptable/useful).

Table 4: Time points of questionnaires in the UPDATE trial.

Visit	Number of questionnaires	Time to complete
Screening	Intake24 only	12 minutes

For peer review only

RCT:<br>• First baseline visit<br><br>Behavioural intervention:<br>• 6-month follow-up<br><br>(Visits 2 and 9)	All 12 questionnaires	90 minutes
RCT:<br>• Week 4<br>• Week 8<br>• Second baseline visit<br><br>(Visits 3, 4, 5, 6 and 7)	11 questionnaires (all except the COM-B healthy eating and PA questionnaire)	60 minutes

COM-B, Capability, Opportunity, Motivation - Behaviour; PA, physical activity; RCT, randomised controlled trial.

Data methods: monitoring

Data monitoring

Data generated from this trial will be handled (including collection, storage, processing and disclosure) in accordance with all applicable legal and regulatory requirements, including the UK Data Protection Act (DPA) 2018 and European Union General Data Protection Regulation (EU GDPR 2016/679). All members of the research team are trained in information governance and research integrity.

All data storage mediums will comply with the NHS Information Governance Toolkit. All physical data will be stored in a secure room, with limited access only to members of the research team. All computers storing electronic data will be encrypted and password protected. Data will be first stored on paper case report forms (CRF), securely kept in a locked cabinet in a locked office, and then recorded electronically on a purpose-built trial database (eCRF) using the REDCap system (https://www.project-redcap.org). REDCap is hosted within the UCL Data Safe Haven, a ISO27001 certified secure environment protected by dedicated firewalls, accessed by role-based user accounts with multi-factor authentication. Each participant has their own eCRF, listed under their participant identification number (PIN). Questionnaires will be provided electronically and stored directly in REDCap. The REDCap database contains range validity checks with warnings for erroneous or missing values, and initial data entry from the paper CRF will be verified by a second team member for quality assurance. The REDCap application also provides a comprehensive audit trail showing all changes to the data.

Data confidentiality is outlined in the PIS (Supplementary Material 1). Participant initials and their PIN will be used on records. The CRF will not bear the participant's name or other personal identifiable data. Identifiable information will be stored in a separate electronic database to the database containing trial data. Participants' personal identity and data

For peer review only

collected in the study cannot be connected by anyone outside the study team. The data generated from this trial will not be transferred to any party not identified in the protocol and will not be processed and/or transferred other than in accordance with the participants' consent. Confidentiality will be maintained by user agreements prior to access, ensuring all researchers uphold the principles of Good Clinical Practice, EU GDPR 679/2016 and DPA 2018.

Retention

Loss to follow-up will be minimised through weekly telephone calls during the RCT and monthly calls during the 6-month behavioural intervention to discuss any issues and adherence, encouraging participants to discuss any difficulties with researchers. Other procedures will be adopted for maintaining participation in the study (e.g., reminders before scheduled trial visits, sending greetings messages, personalising letters, and keeping measures short in terms of completion time). No data will be collected after withdrawal.

Sample size

The sample size of 55 is based on Hall et al., 2019[21] showing 0.9kg weight loss following a 2-week MPF diet, with a standard deviation (SD) of the mean difference in weight change of 1.98kg between MPF and UPF diets (mean: 1.85kg), the predicted weight loss if the MPF diet were continued for 8 weeks [using the National Institutes of Health (NIH) bodyweight planner (https://www.niddk.nih.gov/bwp)].

No interventional studies to date have compared diets differing in the nature, extent and purpose of food processing over this duration. To best estimate the expected difference in %WC over this time-frame, the results from the only controlled feeding trial comparing UPF and MPF diets matched for diet quality on weight change were used (Hall et al., 2019[21]), which lasted for 2 weeks. Inclusion criteria for UPDATE requires participants to have a habitual diet high in UPF, thus the UPF diet is hypothesised at best to lead to no change in weight. Whereas, the MPF diet will result in weight loss, with a similar initial trajectory to that in Hall et al., 2019[21]. The NIH bodyweight planner takes into account that weight change is not linear based on the initial calorie deficit and tends to plateau and considers key factors that influence metabolic rate. The mean age, height, weight, and physical activity levels of the male and female participants from Hall et al., were entered into the bodyweight planner to produce a quantitative estimate of weight change on the MPF diet over 8 weeks, which was converted percentage weight change. Male participants in Hall et al., 2019[21] would achieve 2.8% (2.2kg) weight loss after 8 weeks on the MPF diet, and female participants would achieve 2.7% (2.1kg) weight loss. There is no clear value from the literature as to what the SD should be for the mean difference in weight change between 8-week MPF and UPF diets. The expected SD must therefore be estimated. The 1.98kg value for the SD of the mean difference in weight change from Hall et al., was used as the starting point for the expected SD after 8 weeks. This was around 1.1x the mean difference in weight change between MPF and UPF diet interventions. By assuming that the SD would increase over 8 weeks compared with 2 weeks, the SD was assumed to increase to 2x the mean difference in weight change between MPF and UPF diets. Other trials longer in duration than Hall et al., 2019 were considered, but other trials have a number of fundamental differences to the planned trial, which limits the ability to extrapolate their findings [e.g. not necessarily *ad libitum* or crossover trials, testing diets that are unrelated to the concepts of MPF or UPF, or comparing an intervention to a control diet, (whereas UPDATE involves two interventions)]. These generally reported SDs for

the mean weight change of each group, rather than of the mean difference between groups. For each group over several weeks (e.g., 12 weeks), the SDs tended to be around 0.1-0.9x the mean weight change. Therefore, the Hall et al., 2019 estimate was considered most applicable, relevant and conservative.

Assuming weight loss on the MPF diet and no weight change on the UPF diet, 44 participants are required to detect a mean difference of 2.7% weight change between diets (standard deviation of 5.4% (2x the mean difference), power=0.9, alpha=0.05, with a two-sided paired t-test, using SPSS version 27.0). With a 20% dropout rate based on previous controlled feeding trials[82,83], 55 participants will be recruited. This is comparable to attrition rates in previous multi-arm, community-based crossover trials lasting several months, where participants are provided with all meals[84–87].

The target sample size for the qualitative interviews is 20-30. Data saturation is controversial, but researchers will aim for meaningful saturation, whereby further interviews produce minimal, or no changes to the coding framework and allow complete understanding of thematic codes[88]. Where possible, participants will be selected to ensure representation from across ethnicities, night-shift patterns genders, treatment allocation arms. All participants will be offered the behavioural intervention calls, so will be asked about experiences of the provided trial diets.

Statistical methods

A CONSORT diagram and descriptive statistics will be used to outline the trial sample[89]. The primary outcome will be analysed using mixed-effects models, with a random effect for participants, adjusting for stratification variables and any baseline variables not balanced between arms. Age will also be included as an adjustment covariate. An intention-to-treat analysis will be conducted[90], with values presented by randomisation group and all available data analysed as randomised. Additional models will include a per-protocol analysis, and repeated-measures analysis additionally using data from 4-week follow-ups. Bias from missing data will be dealt with using multiple imputation[91]. The 4-week washout period between diets will minimise any carryover effects[89], with any residual effect assessed between arms. To account for non-adherence (consuming more than one meal per week off the intervention diet), inverse probability weighting will be used to reweight the remaining sample[92]. There is no planned interim analysis.

Secondary outcome variables for the RCT and behavioural intervention will be analysed using mixed-effects or linear, binary or ordinal regression models, where appropriate.

Qualitative interviews will be analysed using Framework Analysis, following the stages outlined by Gale and colleagues[93]. Transcripts will be deductively coded, broadly mapping barriers and facilitators to the COM-B model[37,38] and Theoretical Domains Framework[39] – an approach previously used to understand the experience of novel diets[94], as well as inductively coding aspects that may not naturally fit into these groupings. Recordings will be converted into text using transcription software. Two researchers will initially independently analyse three interviews, before meeting to discuss and develop an initial framework. Additional transcripts will then be coded based on this framework, with additional factors

For peer review only

added as they arise. Two researchers will interpret data and write the report in accordance with the COREQ checklist (www.equator-network.org/reporting-guidelines/coreq).

Methods: Monitoring

A Data and Safety Monitoring Plan (DSMP) plan is in place for quality control to ensure adherence to the approved protocol (Version 1.3, 26/07/23). Charters outline the roles and responsibilities of the Trial Management Group (TMG) and Trial Steering Committee (TSC). The TMG includes the Chief Investigator (CI) (RLB), Trial Coordinator and Manager (SJD) and co-investigators/researchers (AB, CWK, AF, FCJ, JM). The TMG is responsible for maintaining and overseeing the trial, meeting before recruitment and every 3 months throughout the trial. The TMG approved the final trial protocol and eCRF, as well as any subsequent amendments. The TSC will provide supervision and act on behalf of the funders and Sponsor, recommending any appropriate amendment/actions as necessary. The TSC includes a lay member and will be chaired by an external academic member with experience leading clinical trials. The trial may be stopped before completion upon recommendation from the TSC, or Sponsor and CI.

A Data Monitoring Committee will not be set up as there are no intervention-related adverse events (AEs), serious adverse events (SAEs) or major risks associated with participation. Risks are considered minimal, and are outlined in the PIS and CF (Supplementary Materials 1 and 2). Recording/reporting AEs will commence from written informed consent until the 6-month follow-up (week 49). All SAEs will be reported to the Sponsor within 24 hours of becoming aware. Incidental findings will be reported to participants and their GP.

Auditing

Monitoring and auditing (e.g., adherence, deviations, withdrawals, AEs/SAEs) is outlined in the DSMP, and will be conducted by the CI, TMG and TSC in accordance with the UK Policy Framework for Health and Social Care Research, and in accordance with the Sponsor's monitoring and audit policies and procedures.

Ethics and dissemination

Research ethics approval

The Yorkshire & The Humber - Sheffield Research Ethics Committee approved the trial on 22nd December 2022 (22/YH/0281), and the study was prospectively registered on ClinicalTrials.gov (NCT05627570). The trial is currently recruiting and ongoing. The trial will be conducted in compliance with the principles of the Declaration of Helsinki 1996 and the principles of the International Conference on Harmonisation GCP. Any amendments will be recorded in academic publications, and will be submitted for approval to the Sponsor and Research Ethics Committee prior to implementation. Protocol Version 1.3, 26/07/2023. Sponsor contact: (UCLH/UCL) Joint Research Office (uclh.randd@nhs.net). On 16/06/2023, the UPF intake inclusion criterion was amended from ≥60% to ≥50% to better reflect the average intake of participants in London, compared with intakes across the UK[9].

Ancillary and post-trial care

For peer review only

There will be no diet provisions or behavioural support given at the end of the trial. Consent will be sought for future research and the storage and future use of participant samples in the Obesity Research Biobank Syndicate (ORBiS).

Dissemination

Results will be disseminated through publications, conferences and social and lay media. Progress and analyses may be presented to the Sponsor and funding bodies. Authorship will follow the International Committee of Medical Journal Editors guidelines. Participant data will not be made publicly accessible.

Twitter

R.L.B: ProfBatterhamMD
S.J.D: SamuelDickenUK
A.B: BrownAdey
C.W.K: Claudia_WK; @GolgiBrain
K.H: KevinH_PhD
A.F: abifisher99
J.M: DrJanine_Mak
F.C.J: FriedrichJassil
C.v.T: DoctorChrisVT

Author contributions:

R.L.B conceptualised the trial; S.J.D wrote the initial manuscript and protocol draft; R.L.B, S.J.D, A.B, C.W.K, K.H, A.F, J.M, F.C.J and C.v.T contributed to the intervention design, methodology and assessments, R.L.B, S.J.D, A.B, A.F, J.M and F.C.J contributed to the study protocol.

Acknowledgements:

The authors would like to thank: UPDATE participants and collaborators, the Intake24 team (https://intake24.co.uk/), the patient and public involvement focus group, EPIC-Norfolk for use of their FFQ (https://www.epic-norfolk.org.uk/for-researchers/ffq/), REDCap (https://www.project-redcap.org/) and Out Of The Box Food. Acknowledgements also go to Caroline Buck, Gabriella Heuchan and Rana Conway, for their substantial input into the behavioural intervention development.

The statistical analysis plan has been reviewed by an independent associate professor in statistics, (with thanks to Michalis Katsoulis) and a statistician specialising in clinical trial design was consulted regarding the sample size calculation for the proposed statistical analyses (with thanks to Michael Grayling).

Funding:

For peer review only

This work was supported by National Institute for Health and Care Research Biomedical Research Centre (NIHR BRC) grant BRC530a and Rosetrees Trust grant PGL22/100041.

Patient and public involvement:

The trial, including the menu, assessments and participant burden have been reviewed and designed with input from NHS UCLH staff at following a focus group session before ethics submission. In addition, Obesity Empowerment Network UK members, with lived experience of obesity, have also contributed to the design.

Competing Interests:

S.J.D is funded by the Medical Research Council (MR/N013867/1) and receives royalties from Amazon for a self-published book that mentions ultra-processed food, and payments from Red Pen Reviews as a contributor. R.L.B reports honoraria from Novo Nordisk, Eli Lilly, Medscape, ViiV Healthcare Ltd and International Medical P and advisory board and consultancy work for Novo Nordisk, Eli Lilly, Pfizer, Gila Therapeutics Ltd, Epitomee Medical Ltd and ViiV Healthcare Ltd and from May 2023 is an employee and share holder of Eli Lilly and Company. A.B. reports honoraria from Novo Nordisk, Office of Health Improvement and Disparity, Johnson and Johnson and Obesity UK outside the submitted work and is on the Medical Advisory Board and shareholder of Reset Health Clinics Ltd. CGWK receives funding from Horizon2020 (Research and Innovation Action Grants Human Brain Project 945539 (SGA3)), BRC (#BRC704/CAP/CGW), MRC (#MR/S026088/1), Ataxia UK, Rosetrees Trust (#PGL22/100041 and #PGL21/10079) and is a shareholder in Queen Square Analytics Ltd. JM is funded by the NIHR and reports funding from the NIHR BRC and the Society for Endocrinology. JM reports institutional funding from Novo Nordisk, Rhythm Pharmaceuticals and Innovate UK outside the submitted work. KH is supported by the Intramural Research Program of the National Institutes of Health, National Institute of Diabetes & Digestive & Kidney Diseases. C.v.T receives royalties for a book on ultra-processed food. A.F. and F.C.J report no conflicts of interest.

Peer review:

The study has been peer reviewed in accordance with the requirements outlined by UCL. The study was internally peer-reviewed by co-investigators of the research team, and externally peer-reviewed by two peer reviewers at UCL. The protocol has been updated with additional detail following the comments provided by the peer reviewers. Furthermore, the trial was peer-reviewed as part of the funding grant application by Rosetrees Trust by external peer reviewers.

References:

1. NHS Digital Part 3: Adult Overweight and Obesity Available online: https://digital.nhs.uk/data-and-information/publications/statistical/statistics-on-obesity-physical-activity-and-diet/england-2020/part-3-adult-obesity-copy (accessed on 12 July 2021).

For peer review only

2. The Global BMI Mortality Collaboration Body-Mass Index and All-Cause Mortality: Individual-Participant-Data Meta-Analysis of 239 Prospective Studies in Four Continents. *Lancet* **2016**, *388*, 776–786, doi:10.1016/S0140-6736(16)30175-1.
3. Frontier Economics The Annual Social Cost of Obesity in the UK Available online: https://www.frontier-economics.com/uk/en/news-and-articles/articles/article-i9130-the-annual-social-cost-of-obesity-in-the-uk/ (accessed on 26 July 2023).
4. Public Health England Government Recommendations for Energy and Nutrients for Males and Females Aged 1–18 Years and 19+ Years Available online: https://assets.publishing.service.gov.uk/government/uploads/system/uploads/attachment_data/file/618167/government_dietary_recommendations.pdf (accessed on 14 October 2021).
5. Popkin, B.M.; Adair, L.S.; Ng, S.W. The Global Nutrition Transition: The Pandemic of Obesity in Developing Countries. *Nutr Rev* **2012**, *70*, 3–21, doi:10.1111/j.1753-4887.2011.00456.x.
6. Popkin, B.M.; Ng, S.W. The Nutrition Transition to a Stage of High Obesity and Noncommunicable Disease Prevalence Dominated by Ultra-Processed Foods Is Not Inevitable. *Obesity Reviews* **2022**, *23*, e13366, doi:10.1111/obr.13366.
7. Hall, K.D. From Dearth to Excess: The Rise of Obesity in an Ultra-Processed Food System. *Philosophical Transactions of the Royal Society B: Biological Sciences* **2023**, *378*, 20220214, doi:10.1098/rstb.2022.0214.
8. Monteiro, C.A.; Cannon, G.; Levy, R.B.; Moubarac, J.-C.; Louzada, M.L.; Rauber, F.; Khandpur, N.; Cediel, G.; Neri, D.; Martinez-Steele, E.; et al. Ultra-Processed Foods: What They Are and How to Identify Them. *Public Health Nutr* **2019**, *22*, 936–941, doi:10.1017/S1368980018003762.
9. Rauber, F.; Steele, E.M.; Louzada, M.L. da C.; Millett, C.; Monteiro, C.A.; Levy, R.B. Ultra-Processed Food Consumption and Indicators of Obesity in the United Kingdom Population (2008-2016). *PLoS One* **2020**, *15*, doi:10.1371/journal.pone.0232676.
10. Moradi, S.; Entezari, M.H.; Mohammadi, H.; Jayedi, A.; Lazaridi, A.-V.; Kermani, M. ali H.; Miraghajani, M. Ultra-Processed Food Consumption and Adult Obesity Risk: A Systematic Review and Dose-Response Meta-Analysis. *Crit Rev Food Sci Nutr* **2021**, 1–12, doi:10.1080/10408398.2021.1946005.
11. Askari, M.; Heshmati, J.; Shahinfar, H.; Tripathi, N.; Daneshzad, E. Ultra-Processed Food and the Risk of Overweight and Obesity: A Systematic Review and Meta-Analysis of Observational Studies. *Int J Obes (Lond)* **2020**, *44*, 2080–2091, doi:10.1038/s41366-020-00650-z.
12. Juul, F.; Deierlein, A.L.; Vaidean, G.; Quatromoni, P.A.; Parekh, N. Ultra-Processed Foods and Cardiometabolic Health Outcomes: From Evidence to Practice. *Curr Atheroscler Rep* **2022**, *24*, 849–860, doi:10.1007/s11883-022-01061-3.
13. Isaksen, I.M.; Dankel, S.N. Ultra-Processed Food Consumption and Cancer Risk: A Systematic Review and Meta-Analysis. *Clin Nutr* **2023**, *42*, 919–928, doi:10.1016/j.clnu.2023.03.018.
14. Lane, M.M.; Gamage, E.; Travica, N.; Dissanayaka, T.; Ashtree, D.N.; Gauci, S.; Lotfaliany, M.; O'Neil, A.; Jacka, F.N.; Marx, W. Ultra-Processed Food Consumption and Mental Health: A Systematic Review and Meta-Analysis of Observational Studies. *Nutrients* **2022**, *14*, 2568, doi:10.3390/nu14132568.
15. Zhang, S.; Gu, Y.; Rayamajhi, S.; Thapa, A.; Meng, G.; Zhang, Q.; Liu, L.; Wu, H.; Zhang, T.; Wang, X.; et al. Ultra-Processed Food Intake Is Associated with Grip Strength Decline in

For peer review only

Middle-Aged and Older Adults: A Prospective Analysis of the TCLSIH Study. *Eur J Nutr* **2021**, doi:10.1007/s00394-021-02737-3.

16. Dicken, S.J.; Batterham, R.L. The Role of Diet Quality in Mediating the Association between Ultra-Processed Food Intake, Obesity and Health-Related Outcomes: A Review of Prospective Cohort Studies. *Nutrients* **2022**, *14*, 23, doi:10.3390/nu14010023.
17. Scientific Advisory Committee on Nutrition Carbohydrates and Health Report Available online: https://www.gov.uk/government/publications/sacn-carbohydrates-and-health-report (accessed on 5 February 2020).
18. NHS The Eatwell Guide Available online: https://www.nhs.uk/live-well/eat-well/the-eatwell-guide/ (accessed on 11 October 2021).
19. European Commission Nutrition Claims Available online: https://ec.europa.eu/food/safety/labelling-and-nutrition/nutrition-and-health-claims/nutrition-claims_en (accessed on 7 November 2021).
20. Derbyshire, E. Are All 'Ultra-Processed' Foods Nutritional Demons? A Commentary and Nutritional Profiling Analysis. *Trends Food Sci Technol* **2019**, *94*, 98–104, doi:10.1016/j.tifs.2019.08.023.
21. Hall, K.D.; Ayuketah, A.; Brychta, R.; Cai, H.; Cassimatis, T.; Chen, K.Y.; Chung, S.T.; Costa, E.; Courville, A.; Darcey, V.; et al. Ultra-Processed Diets Cause Excess Calorie Intake and Weight Gain: An Inpatient Randomized Controlled Trial of Ad Libitum Food Intake. *Cell Metab* **2019**, *30*, 67-77.e3, doi:10.1016/j.cmet.2019.05.008.
22. Cordova, R.; Kliemann, N.; Huybrechts, I.; Rauber, F.; Vamos, E.P.; Levy, R.B.; Wagner, K.-H.; Viallon, V.; Casagrande, C.; Nicolas, G.; et al. Consumption of Ultra-Processed Foods Associated with Weight Gain and Obesity in Adults: A Multi-National Cohort Study. *Clin Nutr* **2021**, *40*, 5079–5088, doi:10.1016/j.clnu.2021.08.009.
23. British Nutrition Foundation Position Statement on the Concept of Ultra-Processed Foods (UPF) - British Nutrition Foundation Available online: https://www.nutrition.org.uk/news/2023/position-statement-on-the-concept-of-ultra-processed-foods-upf/ (accessed on 7 July 2023).
24. GOV.UK SACN Statement on Processed Foods and Health Available online: https://www.gov.uk/government/publications/sacn-statement-on-processed-foods-and-health (accessed on 11 July 2023).
25. Kyle, R.G.; Wills, J.; Mahoney, C.; Hoyle, L.; Kelly, M.; Atherton, I.M. Obesity Prevalence among Healthcare Professionals in England: A Cross-Sectional Study Using the Health Survey for England. *BMJ Open* **2017**, *7*, e018498, doi:10.1136/bmjopen-2017-018498.
26. Blake, H.; Mo, P.K.; Lee, S.; Batt, M.E. Health in the NHS: Lifestyle Behaviours of Hospital Employees. *Perspect Public Health* **2012**, *132*, 213–215, doi:10.1177/1757913912457309.
27. Power, B.T.; Kiezebrink, K.; Allan, J.L.; Campbell, M.K. Understanding Perceived Determinants of Nurses' Eating and Physical Activity Behaviour: A Theory-Informed Qualitative Interview Study. *BMC Obes* **2017**, *4*, 18, doi:10.1186/s40608-017-0154-4.
28. Dicken, S.J.; Batterham, R.L. Ultra-Processed Food: A Global Problem Requiring a Global Solution. *Lancet Diabetes Endocrinol* **2022**, *10*, 691–694, doi:10.1016/S2213-8587(22)00248-0.
29. Chan, A.-W.; Tetzlaff, J.M.; Altman, D.G.; Laupacis, A.; Gøtzsche, P.C.; Krleža-Jerić, K.; Hróbjartsson, A.; Mann, H.; Dickersin, K.; Berlin, J.A.; et al. SPIRIT 2013 Statement:

For peer review only

Defining Standard Protocol Items for Clinical Trials. *Ann Intern Med* **2013**, *158*, 200–207, doi:10.7326/0003-4819-158-3-201302050-00583.

30. Hall, D.M.; Most, M.M. Dietary Adherence in Well-Controlled Feeding Studies. *Journal of the American Dietetic Association* **2005**, *105*, 1285–1288, doi:10.1016/j.jada.2005.05.009.
31. Moreira, E.A.M.; Most, M.; Howard, J.; Ravussin, E. Dietary Adherence to Long-Term Controlled Feeding in a Calorie-Restriction Study in Overweight Men and Women. *Nutr Clin Pract* **2011**, *26*, 309–315, doi:10.1177/0884533611405992.
32. Public Health England National Diet and Nutrition Survey: Assessment of Salt Intake from Urinary Sodium in Adults Available online: https://assets.publishing.service.gov.uk/government/uploads/system/uploads/attachment_data/file/876624/Report_England_Sodium_Survey_2018-to-2019.pdf.
33. NDNS: Results from Years 9 to 11 (2016 to 2017 and 2018 to 2019) Available online: https://www.gov.uk/government/statistics/ndns-results-from-years-9-to-11-2016-to-2017-and-2018-to-2019 (accessed on 30 November 2021).
34. Rolls, B.J.; Rolls, E.T.; Rowe, E.A.; Sweeney, K. Sensory Specific Satiety in Man. *Physiol Behav* **1981**, *27*, 137–142, doi:10.1016/0031-9384(81)90310-3.
35. Department of Health and Social Care Alcohol Consumption: Advice on Low Risk Drinking Available online: https://www.gov.uk/government/publications/alcohol-consumption-advice-on-low-risk-drinking (accessed on 27 May 2021).
36. Poppitt, S.D.; Keogh, G.F.; Prentice, A.M.; Williams, D.E.M.; Sonnemans, H.M.W.; Valk, E.E.J.; Robinson, E.; Wareham, N.J. Long-Term Effects of Ad Libitum Low-Fat, High-Carbohydrate Diets on Body Weight and Serum Lipids in Overweight Subjects with Metabolic Syndrome. *Am J Clin Nutr* **2002**, *75*, 11–20, doi:10.1093/ajcn/75.1.11.
37. Skivington, K.; Matthews, L.; Simpson, S.A.; Craig, P.; Baird, J.; Blazeby, J.M.; Boyd, K.A.; Craig, N.; French, D.P.; McIntosh, E.; et al. A New Framework for Developing and Evaluating Complex Interventions: Update of Medical Research Council Guidance. *BMJ* **2021**, *374*, n2061, doi:10.1136/bmj.n2061.
38. Michie, S.; van Stralen, M.M.; West, R. The Behaviour Change Wheel: A New Method for Characterising and Designing Behaviour Change Interventions. *Implement. Sci.* **2011**, *6*, 42, doi:10.1186/1748-5908-6-42.
39. Cane, J.; O'Connor, D.; Michie, S. Validation of the Theoretical Domains Framework for Use in Behaviour Change and Implementation Research. *Implement Sci* **2012**, *7*, 37, doi:10.1186/1748-5908-7-37.
40. Morrow, A.; Walker, K.; Calder-MacPhee, N.; Ozakinci, G. The Active Ingredients of Physical Activity and / or Dietary Workplace-Based Interventions to Achieve Weight Loss in Overweight and Obese Healthcare Staff: A Systematic Review. *J Behav Med* **2022**, *45*, 331–349, doi:10.1007/s10865-021-00279-x.
41. Michie, S.; Abraham, C.; Whittington, C.; Mcateer, J.; Gupta, S. Effective Techniques in Healthy Eating and Physical Activity Interventions: A Meta-Regression. *Health psychology : official journal of the Division of Health Psychology, American Psychological Association* **2009**, *28*, 690–701, doi:10.1037/a0016136.
42. Samdal, G.B.; Eide, G.E.; Barth, T.; Williams, G.; Meland, E. Effective Behaviour Change Techniques for Physical Activity and Healthy Eating in Overweight and Obese Adults; Systematic Review and Meta-Regression Analyses. *Int J Behav Nutr Phys Act* **2017**, *14*, doi:10.1186/s12966-017-0494-y.

For peer review only

43. Jensen, M.D.; Ryan, D.H.; Apovian, C.M.; Ard, J.D.; Comuzzie, A.G.; Donato, K.A.; Hu, F.B.; Hubbard, V.S.; Jakicic, J.M.; Kushner, R.F.; et al. 2013 AHA/ACC/TOS Guideline for the Management of Overweight and Obesity in Adults. *Circulation* **2014**, *129*, S102–S138, doi:10.1161/01.cir.0000437739.71477.ee.
44. Buckell, J.; Mei, X.W.; Clarke, P.; Aveyard, P.; Jebb, S.A. Weight Loss Interventions on Health-Related Quality of Life in Those with Moderate to Severe Obesity: Findings from an Individual Patient Data Meta-Analysis of Randomized Trials. *Obesity Reviews* **2021**, *22*, e13317, doi:10.1111/obr.13317.
45. Ross, R.; Neeland, I.J.; Yamashita, S.; Shai, I.; Seidell, J.; Magni, P.; Santos, R.D.; Arsenault, B.; Cuevas, A.; Hu, F.B.; et al. Waist Circumference as a Vital Sign in Clinical Practice: A Consensus Statement from the IAS and ICCR Working Group on Visceral Obesity. *Nat Rev Endocrinol* **2020**, *16*, 177–189, doi:10.1038/s41574-019-0310-7.
46. Flint, A.; Raben, A.; Blundell, J.E.; Astrup, A. Reproducibility, Power and Validity of Visual Analogue Scales in Assessment of Appetite Sensations in Single Test Meal Studies. *Int J Obes Relat Metab Disord* **2000**, *24*, 38–48, doi:10.1038/sj.ijo.0801083.
47. Powell, C.; Carson, B.P.; Dowd, K.P.; Donnelly, A.E. Simultaneous Validation of Five Activity Monitors for Use in Adult Populations. *Scandinavian Journal of Medicine & Science in Sports* **2017**, *27*, 1881–1892, doi:10.1111/sms.12813.
48. Lee, P.H.; Macfarlane, D.J.; Lam, T.H.; Stewart, S.M. Validity of the International Physical Activity Questionnaire Short Form (IPAQ-SF): A Systematic Review. *Int J Behav Nutr Phys Act* **2011**, *8*, 115, doi:10.1186/1479-5868-8-115.
49. Meh, K.; Jurak, G.; Sorić, M.; Rocha, P.; Sember, V. Validity and Reliability of IPAQ-SF and GPAQ for Assessing Sedentary Behaviour in Adults in the European Union: A Systematic Review and Meta-Analysis. *Int J Environ Res Public Health* **2021**, *18*, 4602, doi:10.3390/ijerph18094602.
50. Sember, V.; Meh, K.; Sorić, M.; Starc, G.; Rocha, P.; Jurak, G. Validity and Reliability of International Physical Activity Questionnaires for Adults across EU Countries: Systematic Review and Meta Analysis. *Int J Environ Res Public Health* **2020**, *17*, E7161, doi:10.3390/ijerph17197161.
51. Craig, C.L.; Marshall, A.L.; Sjöström, M.; Bauman, A.E.; Booth, M.L.; Ainsworth, B.E.; Pratt, M.; Ekelund, U.; Yngve, A.; Sallis, J.F.; et al. International Physical Activity Questionnaire: 12-Country Reliability and Validity. *Medicine & Science in Sports & Exercise* **2003**, *35*, 1381–1395, doi:10.1249/01.MSS.0000078924.61453.FB.
52. Buysse, D.J.; Reynolds, C.F.; Monk, T.H.; Berman, S.R.; Kupfer, D.J. The Pittsburgh Sleep Quality Index: A New Instrument for Psychiatric Practice and Research. *Psychiatry Res* **1989**, *28*, 193–213, doi:10.1016/0165-1781(89)90047-4.
53. Beriault, K.; Carpentier, A.C.; Gagnon, C.; Ménard, J.; Baillargeon, J.-P.; Ardilouze, J.-L.; Langlois, M.-F. Reproducibility of the 6-Minute Walk Test in Obese Adults. *Int J Sports Med* **2009**, *30*, 725–727, doi:10.1055/s-0029-1231043.
54. ATS Committee on Proficiency Standards for Clinical Pulmonary Function Laboratories ATS Statement: Guidelines for the Six-Minute Walk Test. *Am J Respir Crit Care Med* **2002**, *166*, 111–117, doi:10.1164/ajrccm.166.1.at1102.
55. Klukowska, A.M.; Staartjes, V.E.; Vandertop, W.P.; Schröder, M.L. Five-Repetition Sit-to-Stand Test Performance in Healthy Individuals: Reference Values and Predictors From 2 Prospective Cohorts. *Neurospine* **2021**, *18*, 760–769, doi:10.14245/ns.2142750.375.
56. EuroQol EQ-5D-3L – EQ-5D.

For peer review only

57. Kolotkin, R.L.; Crosby, R.D. Psychometric Evaluation of the Impact of Weight on Quality of Life-Lite Questionnaire (IWQOL-Lite) in a Community Sample. *Qual Life Res* **2002**, *11*, 157–171, doi:10.1023/a:1015081805439.
58. Tennant, R.; Hiller, L.; Fishwick, R.; Platt, S.; Joseph, S.; Weich, S.; Parkinson, J.; Secker, J.; Stewart-Brown, S. The Warwick-Edinburgh Mental Well-Being Scale (WEMWBS): Development and UK Validation. *Health Qual Life Outcomes* **2007**, *5*, 63, doi:10.1186/1477-7525-5-63.
59. Kroenke, K.; Spitzer, R.L.; Williams, J.B.W. The PHQ-9. *J Gen Intern Med* **2001**, *16*, 606–613, doi:10.1046/j.1525-1497.2001.016009606.x.
60. Spitzer, R.L.; Kroenke, K.; Williams, J.B.W.; Löwe, B. A Brief Measure for Assessing Generalized Anxiety Disorder: The GAD-7. *Arch Intern Med* **2006**, *166*, 1092–1097, doi:10.1001/archinte.166.10.1092.
61. Lowe, M.R.; Butryn, M.L.; Didie, E.R.; Annunziato, R.A.; Thomas, J.G.; Crerand, C.E.; Ochner, C.N.; Coletta, M.C.; Bellace, D.; Wallaert, M.; et al. The Power of Food Scale. A New Measure of the Psychological Influence of the Food Environment. *Appetite* **2009**, *53*, 114–118, doi:10.1016/j.appet.2009.05.016.
62. Dalton, M.; Finlayson, G.; Hill, A.; Blundell, J. Preliminary Validation and Principal Components Analysis of the Control of Eating Questionnaire (CoEQ) for the Experience of Food Craving. *Eur J Clin Nutr* **2015**, *69*, 1313–1317, doi:10.1038/ejcn.2015.57.
63. Willmott, T.J.; Pang, B.; Rundle-Thiele, S. Capability, Opportunity, and Motivation: An across Contexts Empirical Examination of the COM-B Model. *BMC Public Health* **2021**, *21*, 1014, doi:10.1186/s12889-021-11019-w.
64. Gearhardt, A.N.; Schulte, E.M. Is Food Addictive? A Review of the Science. *Annu Rev Nutr* **2021**, *41*, 387–410, doi:10.1146/annurev-nutr-110420-111710.
65. David, I.A.; Krutman, L.; Fernández-Santaella, M.C.; Andrade, J.R.; Andrade, E.B.; Oliveira, L.; Pereira, M.G.; Gomes, F.S.; Gleiser, S.; Oliveira, J.M.; et al. Appetitive Drives for Ultra-Processed Food Products and the Ability of Text Warnings to Counteract Consumption Predispositions. *Public Health Nutrition* **2018**, *21*, 543–557, doi:10.1017/S1368980017003263.
66. Batterham, R.L.; ffytche, D.H.; Rosenthal, J.M.; Zelaya, F.O.; Barker, G.J.; Withers, D.J.; Williams, S.C.R. PYY Modulation of Cortical and Hypothalamic Brain Areas Predicts Feeding Behaviour in Humans. *Nature* **2007**, *450*, 106–109, doi:10.1038/nature06212.
67. Wingrove, J.; Makaronidis, J.; Prados, F.; Kanber, B.; Yiannakas, M.C.; Magee, C.; Castellazzi, G.; Grandjean, L.; Golay, X.; Tur, C.; et al. Aberrant Olfactory Network Functional Connectivity in People with Olfactory Dysfunction Following COVID-19 Infection: An Exploratory, Observational Study. *EClinicalMedicine* **2023**, *58*, 101883, doi:10.1016/j.eclinm.2023.101883.
68. Zhang, H.; Schneider, T.; Wheeler-Kingshott, C.A.; Alexander, D.C. NODDI: Practical in Vivo Neurite Orientation Dispersion and Density Imaging of the Human Brain. *NeuroImage* **2012**, *61*, 1000–1016, doi:10.1016/j.neuroimage.2012.03.072.
69. Monteverdi, A.; Palesi, F.; Costa, A.; Vitali, P.; Pichiecchio, A.; Cotta Ramusino, M.; Bernini, S.; Jirsa, V.; Gandini Wheeler-Kingshott, C.A.M.; D’Angelo, E. Subject-Specific Features of Excitation/Inhibition Profiles in Neurodegenerative Diseases. *Frontiers in Aging Neuroscience* **2022**, *14*.
70. Solanky, B.S.; John, N.A.; DeAngelis, F.; Stutters, J.; Prados, F.; Schneider, T.; Parker, R.A.; Weir, C.J.; Monteverdi, A.; Plantone, D.; et al. NAA Is a Marker of Disability in

For peer review only

Secondary-Progressive MS: A Proton MR Spectroscopic Imaging Study. *AJNR Am J Neuroradiol* **2020**, *41*, 2209–2218, doi:10.3174/ajnr.A6809.

71. Feldotto, B.; Eppler, J.M.; Jimenez-Romero, C.; Bignamini, C.; Gutierrez, C.E.; Albanese, U.; Retamino, E.; Vorobev, V.; Zolfaghari, V.; Upton, A.; et al. Deploying and Optimizing Embodied Simulations of Large-Scale Spiking Neural Networks on HPC Infrastructure. *Front Neuroinform* **2022**, *16*, 884180, doi:10.3389/fninf.2022.884180.
72. Williamson, D.A.; Anton, S.D.; Han, H.; Champagne, C.M.; Allen, R.; LeBlanc, E.; Ryan, D.H.; McManus, K.; Laranjo, N.; Carey, V.J.; et al. Adherence Is a Multi-Dimensional Construct in the POUNDS LOST Trial. *J Behav Med* **2010**, *33*, 35–46, doi:10.1007/s10865-009-9230-7.
73. Simpson, E.; Bradley, J.; Poliakov, I.; Jackson, D.; Olivier, P.; Adamson, A.J.; Foster, E. Iterative Development of an Online Dietary Recall Tool: INTAKE24. *Nutrients* **2017**, *9*, E118, doi:10.3390/nu9020118.
74. Bradley, J.; Simpson, E.; Poliakov, I.; Matthews, J.N.S.; Olivier, P.; Adamson, A.J.; Foster, E. Comparison of INTAKE24 (an Online 24-h Dietary Recall Tool) with Interviewer-Led 24-h Recall in 11-24 Year-Old. *Nutrients* **2016**, *8*, E358, doi:10.3390/nu8060358.
75. Foster, E.; Lee, C.; Imamura, F.; Hollidge, S.E.; Westgate, K.L.; Venables, M.C.; Poliakov, I.; Rowland, M.K.; Osadchiy, T.; Bradley, J.C.; et al. Validity and Reliability of an Online Self-Report 24-h Dietary Recall Method (Intake24): A Doubly Labelled Water Study and Repeated-Measures Analysis. *J Nutr Sci* **2019**, *8*, e29, doi:10.1017/jns.2019.20.
76. Food Standards Agency *Comparison Study: INTAKE24 vs Interviewer Led Recall Final Report*; 2014;
77. Dicken, S.J.; Batterham, R.L.; Brown, A. Nutrients or Processing? An Analysis of Food and Drink Items from the UK National Diet and Nutrition Survey Based on Nutrient Content, the NOVA Classification, and Front of Package Traffic Light Labelling. *medRxiv* **2023**, 2023.04.24.23289024, doi:10.1101/2023.04.24.23289024.
78. Bingham, S.A.; Welch, A.A.; McTaggart, A.; Mulligan, A.A.; Runswick, S.A.; Luben, R.; Oakes, S.; Khaw, K.T.; Wareham, N.; Day, N.E. Nutritional Methods in the European Prospective Investigation of Cancer in Norfolk. *Public Health Nutr* **2001**, *4*, 847–858, doi:10.1079/phn2000102.
79. McKeown, N.M.; Day, N.E.; Welch, A.A.; Runswick, S.A.; Luben, R.N.; Mulligan, A.A.; McTaggart, A.; Bingham, S.A. Use of Biological Markers to Validate Self-Reported Dietary Intake in a Random Sample of the European Prospective Investigation into Cancer United Kingdom Norfolk Cohort. *Am J Clin Nutr* **2001**, *74*, 188–196, doi:10.1093/ajcn/74.2.188.
80. Bingham, S.A.; Gill, C.; Welch, A.; Cassidy, A.; Runswick, S.A.; Oakes, S.; Lubin, R.; Thurnham, D.I.; Key, T.J.; Roe, L.; et al. Validation of Dietary Assessment Methods in the UK Arm of EPIC Using Weighed Records, and 24-Hour Urinary Nitrogen and Potassium and Serum Vitamin C and Carotenoids as Biomarkers. *Int J Epidemiol* **1997**, *26 Suppl 1*, S137-151, doi:10.1093/ije/26.suppl_1.s137.
81. Freedman, L.S.; Midthune, D.; Arab, L.; Prentice, R.L.; Subar, A.F.; Willett, W.; Neuhouser, M.L.; Tinker, L.F.; Kipnis, V. Combining a Food Frequency Questionnaire With 24-Hour Recalls to Increase the Precision of Estimation of Usual Dietary Intakes-Evidence From the Validation Studies Pooling Project. *Am J Epidemiol* **2018**, *187*, 2227–2232, doi:10.1093/aje/kwy126.
82. Barnard, N.D.; Alwarith, J.; Rembert, E.; Brandon, L.; Nguyen, M.; Goergen, A.; Horne, T.; do Nascimento, G.F.; Lakkadi, K.; Tura, A.; et al. A Mediterranean Diet and Low-Fat

For peer review only

Vegan Diet to Improve Body Weight and Cardiometabolic Risk Factors: A Randomized, Cross-over Trial. *Journal of the American Nutrition Association* **2022**, *41*, 127–139, doi:10.1080/07315724.2020.1869625.
83. Fuller, N.R.; Lau, N.S.; Denyer, G.; Simpson, A.E.; Gerofi, J.; Wu, M.; Holmes, A.; Markovic, T.P.; Kang, J.-H.; Caterson, I.D. A 12-Week, Randomised, Controlled Trial to Examine the Acceptability of the Korean Diet and Its Effectiveness on Weight and Metabolic Parameters in an Australian Overweight and Obese Population. *Obesity Research & Clinical Practice* **2012**, *6*, e71–e83, doi:10.1016/j.orcp.2011.05.003.
84. Sacks, F.M.; Carey, V.J.; Anderson, C.A.M.; Miller, E.R.; Copeland, T.; Charleston, J.; Harshfield, B.J.; Laranjo, N.; McCarron, P.; Swain, J.; et al. Effects of High vs Low Glycemic Index of Dietary Carbohydrate on Cardiovascular Disease Risk Factors and Insulin Sensitivity. *JAMA* **2014**, *312*, 2531–2541, doi:10.1001/jama.2014.16658.
85. Appel, L.J.; Sacks, F.M.; Carey, V.J.; Obarzanek, E.; Swain, J.F.; Miller, E.R.; Conlin, P.R.; Erlinger, T.P.; Rosner, B.A.; Laranjo, N.M.; et al. Effects of Protein, Monounsaturated Fat, and Carbohydrate Intake on Blood Pressure and Serum LipidsResults of the OmniHeart Randomized Trial. *JAMA* **2005**, *294*, 2455–2464, doi:10.1001/jama.294.19.2455.
86. Ginsberg, H.N.; Kris-Etherton, P.; Dennis, B.; Elmer, P.J.; Ershow, A.; Lefevre, M.; Pearson, T.; Roheim, P.; Ramakrishnan, R.; Reed, R.; et al. Effects of Reducing Dietary Saturated Fatty Acids on Plasma Lipids and Lipoproteins in Healthy Subjects. *Arteriosclerosis, Thrombosis, and Vascular Biology* **1998**, *18*, 441–449, doi:10.1161/01.ATV.18.3.441.
87. Berglund, L.; Lefevre, M.; Ginsberg, H.N.; Kris-Etherton, P.M.; Elmer, P.J.; Stewart, P.W.; Ershow, A.; Pearson, T.A.; Dennis, B.H.; Roheim, P.S.; et al. Comparison of Monounsaturated Fat with Carbohydrates as a Replacement for Saturated Fat in Subjects with a High Metabolic Risk Profile: Studies in the Fasting and Postprandial States. *The American Journal of Clinical Nutrition* **2007**, *86*, 1611–1620, doi:10.1093/ajcn/86.5.1611.
88. Palinkas, L.A.; Horwitz, S.M.; Green, C.A.; Wisdom, J.P.; Duan, N.; Hoagwood, K. Purposeful Sampling for Qualitative Data Collection and Analysis in Mixed Method Implementation Research. *Adm Policy Ment Health* **2015**, *42*, 533–544, doi:10.1007/s10488-013-0528-y.
89. Dwan, K.; Li, T.; Altman, D.G.; Elbourne, D. CONSORT 2010 Statement: Extension to Randomised Crossover Trials. *BMJ* **2019**, *366*, l4378, doi:10.1136/bmj.l4378.
90. White, I.R.; Horton, N.J.; Carpenter, J.; Pocock, S.J. Strategy for Intention to Treat Analysis in Randomised Trials with Missing Outcome Data. *BMJ* **2011**, *342*, d40, doi:10.1136/bmj.d40.
91. Jakobsen, J.C.; Gluud, C.; Wetterslev, J.; Winkel, P. When and How Should Multiple Imputation Be Used for Handling Missing Data in Randomised Clinical Trials – a Practical Guide with Flowcharts. *BMC Med Res Methodol* **2017**, *17*, 162, doi:10.1186/s12874-017-0442-1.
92. Hernán, M.A.; Robins, J.M. Per-Protocol Analyses of Pragmatic Trials. *New England Journal of Medicine* **2017**, *377*, 1391–1398, doi:10.1056/NEJMsm1605385.
93. Gale, N.K.; Heath, G.; Cameron, E.; Rashid, S.; Redwood, S. Using the Framework Method for the Analysis of Qualitative Data in Multi-Disciplinary Health Research. *BMC Medical Research Methodology* **2013**, *13*, 117, doi:10.1186/1471-2288-13-117.

For peer review only - http://bmjopen.bmj.com/site/about/guidelines.xhtml

94. Timlin, D.; McCormack, J.M.; Simpson, E.E. Using the COM-B Model to Identify Barriers and Facilitators towards Adoption of a Diet Associated with Cognitive Function (MIND Diet). *Public Health Nutr 24*, 1657–1670, doi:10.1017/S1368980020001445.

For peer review only

POPULATION: UCLH staff living with overweight or obesity

↓

WRITTEN INFORMED CONSENT

↓

SCREENING ASSESSMENT: Sociodemographics; medical history; medications; height, weight; BP and HR; urine pregnancy test; diet assessment

↓

BASELINE DIET ASSESSMENT (Weeks 1-2)

→ **EXCLUDED: Screening failure or baseline diet assessment failure**

↓

2x2 RCT PHASE: RANDOMISATION FOR DIET ORDER: 1:1 single-blind allocation (n=55)
Stratified by sex, ethnicity and night-shift status

↓

BASELINE ASSESSMENT 1 (Weeks 1-2): History; medications; weight; WC; BIA; BP and HR; blood tests; meal test; actigraphy; physical function and strength; questionnaires; MRI in subset; AEs

↓

FIRST 8 WEEK DIET INTERVENTION

4-WEEK FOLLOW-UP VISIT (Week 6): Medical review; weight; WC; BIA; BP and HR; physical function and strength; diet assessment; questionnaires; AEs

8-WEEK FOLLOW-UP VISIT (Week 10): Medical review; weight; WC; BIA; BP and HR; blood tests; meal test; actigraphy; physical function and strength; diet assessment; questionnaires; MRI in subset; AEs

↓

WASH OUT PERIOD (Weeks 11-14): Return to habitual diet

↓

BASELINE ASSESSMENT 2 (Weeks 15-16): Medical review; medications; weight; WC; BIA; BP and HR; blood tests; meal test; actigraphy; physical function and strength; diet assessment; questionnaires; MRI in subset; AEs

↓

SECOND 8 WEEK DIET INTERVENTION

4-WEEK FOLLOW-UP VISIT (Week 20): Medical review; weight; WC; BIA; BP and HR; physical function and strength; diet assessment; questionnaires; AEs

8-WEEK FOLLOW-UP VISIT (Week 24): Medical review; weight; WC; BIA; BP and HR; blood tests; meal test; actigraphy; physical function and strength; diet assessment; questionnaires; MRI in subset; AEs

↓

6-MONTH BEHAVIOURAL SUPPORT PROGRAM

WEEK 24-49: Monthly behavioural support calls and provision of resources to support reduction of UPF intake and increase in physical activity levels

↓

6-MONTH FOLLOW-UP (Week 49): Medical review; medications; weight; WC; BIA; BP and HR; blood tests; meal test; actigraphy; physical function and strength; diet assessment; questionnaires; qualitative interview; AEs

For peer review only - http://bmjopen.bmj.com/site/about/guidelines.xhtml

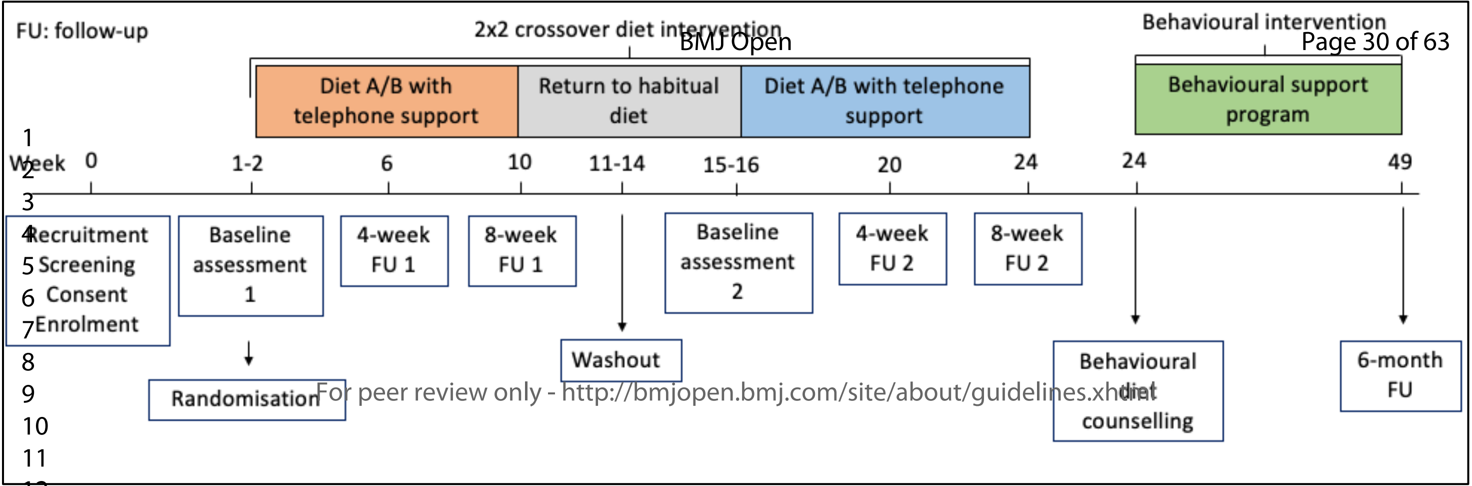

For peer review only - http://bmjopen.bmj.com/site/about/guidelines.xhtml

PARTICIPANT INFORMATION SHEET

Sponsor Protocol Number: 151582
IRAS ID number: 311525

Study Title: UPDATE Trial: A study comparing the health effects of two diets following UK dietary guidance in people living with overweight or obesity

Name of Chief Investigator: Professor Rachel L. Batterham

Study team:
Professor Claudia Wheeler-Kingshott, Dr Adrian Brown, Dr Abigail Fisher, Dr Chris van Tulleken, Dr Janine Makaronidis, Dr Jed Wingrove, Tapiwa Ruwona, Samuel Dicken

Thank you for taking the time to read this participant information sheet (PIS). We would like to invite you to take part in our study investigating the benefit of eating a healthy, balanced diet as recommended by UK governments. The results from this study will be used to help inform UK dietary guidance and food policy. Before you decide if you would like to take part, it is important that you understand why this research is being done and what it will involve. Please take the time to decide whether or not you wish to take part. A member of the study team will call you in the next week to go through this information sheet with you and answer any questions you may have. You may also wish to discuss this information with your GP, your family or friends before deciding if you would like to take part.

Ask us if there is anything that is not clear. Take time to decide whether or not you wish to take part. We appreciate that this information sheet may not answer all of your questions, so please do not hesitate to contact a member of the study team on the telephone numbers given at the end of this information sheet if you would like to discuss any aspect of the study further.

Thank you for taking the time to read this information.

For peer review only

For peer review only - http://bmjopen.bmj.com/site/about/guidelines.xhtml

Study summary:

- **UK governments recommend a healthy, balanced diet, which involves eating a variety of foods in the right proportions, such as five daily portions of fruit and vegetables, and limiting foods high in saturated fat, added sugar and salt.**
- **We want to see if the benefit of a healthy, balanced diet depends on the types of food processing in the diet.**
- **Staff at University College London Hospitals (UCLH) living with overweight or obesity will be invited to take part.**
- **There are two parts to this study, the first part is a diet intervention, the second part is behavioural support intervention to eat a healthier diet and be more active.**
- **In the first part, you will be given two diets in a random order, for 8 weeks each.**
- **Both diets are healthy and balanced, but differ in the types of food processing.**
- **After the first 8-week diet, you will return to your normal diet for 4 weeks, before we give you the second 8-week diet.**
- **We will not tell you which diet you will be given first.**
- **You will be given all your meals, snacks and drinks for both 8-week diets for free.**
- **After the second 8-week diet, we will then support you to eat a healthier diet and be more physically active for 6 months.**
- **We will collect data from you before you start both diets, at 4 weeks and 8 weeks into each diet, and then at the end of the 6-month support program.**
- **We will collect data about your health, including blood pressure, body composition, blood samples, physical activity and fitness, sleep and quality of life.**
- **The risk associated with taking part in the study is low, and no adverse events are expected from taking part.**
- **You can leave the study at any time, without giving a reason.**
- **You will be involved in the study for 49 weeks.**

For peer review only - http://bmjopen.bmj.com/site/about/guidelines.xhtml

1. What is the purpose of the study?

Eating a healthy, balanced diet is important for our health. A healthy, balanced diet involves eating a variety of foods in the right proportions. In the UK, the Eatwell Guide gives us recommendations on what we should eat more of, and what we should eat less of. Unhealthy diets contain lots of foods high in saturated fat, added sugar and salt, which increases our risk of developing obesity, cardiovascular disease and type 2 diabetes. Advice for a healthy, balanced diet includes consuming five portions of fruit and vegetables per day and eating more high fibre foods. Following the healthy, balanced diet advice given in the Eatwell Guide can reduce our risk of poor health.

However, there are other parts of our diet that are not covered in the Eatwell Guide that may also be important for health. For example, research suggests that some types of food processing might influence our health, but these types of food processing are not included within the recommendations.

We do not know if the benefit of following the healthy diet advice in the Eatwell Guide depends on the types of food processing in our diets. It is important that we find out if these types of food processing matter, in order to give people the best advice possible. It also means that the government and other health organisations may need to change the regulations around the food we eat.

To answer this, we will compare the effects of two healthy, balanced diets following the advice in the UK Eatwell Guide, but each based on a different type of food processing. Participants will receive the first diet for 8 weeks, return to their normal diet for 4 weeks, and then receive the second diet for a further 8 weeks. They will have 6 months of support to help improve their diet and be more physically active. The results from this study will be used to help inform UK dietary guidance and food policy.

The study will also contribute towards the award of a PhD at University College London (UCL).

There are two parts to this study:

1. We will study if the benefit from eating a healthy, balanced diet depends on the types of food processing in the diet. We will do this by providing people with two diets that follow the Eatwell Guide, but containing foods with different types of processing, for 8 weeks each.
2. We will then study whether people are able to switch from their usual unhealthy diet to a healthy, balanced diet, and the benefits of doing so. We will do this by providing people with 6 months of personal support. We will also look at what helps people to maintain a healthy diet, and what makes it difficult. We will also support people to be more physically active.

2. Why have I been invited?

You have been invited to take part because you are a member of staff at UCL Hospitals (UCLH) living with overweight or obesity, and you have provided interest in participating in the study by contacting our research team.

For peer review only - http://bmjopen.bmj.com/site/about/guidelines.xhtml

However, you will be unable to take part if you have dietary restrictions that limit you from consuming the provided diets, such as having food allergies, or being vegan or vegetarian. You also cannot take part if you are pregnant and/or breastfeeding, or living with diabetes.

3. Do I have to take part?

No, the decision to take part is completely up to you. No one should force you into taking part. A member of the study team will contact you by telephone or email within a week of you receiving this invitation letter to see if you are still interested in taking part. We will describe the study and go through this information sheet. You can discuss any further questions you may have about the study with them. If you choose not to take part, that is completely fine. This will not affect any care you receive from the NHS, nor affect your role at UCLH. If you agree to take part, we will then ask you to sign a consent form. Even after signing the consent form, you are still able to withdraw from the study at any time without giving a reason. This would not affect the standard of any care you receive.

4. What will happen to me if I decide to take part?

If you decide to take part, you will be asked to sign two copies of the trial consent form. You will be given a copy to take home and keep. An appointment at a time and day convenient for you will be arranged for you to attend a screening visit. Signing the consent form however will not automatically enrol you onto the trial. Your eligibility to participate will depend on the data obtained during the screening visit. The research team will notify you on the outcome of this.

If you decide to take part, you will first be given one of the two diets for 8 weeks. You will then return to your normal diet for 4 weeks, before being given the second diet for another 8 weeks. After the second diet, you will be given 6 months of support to help you switch to and maintain a healthy, balanced diet and be more physically active.

For part 1 of this study, we will see if the benefit of consuming a healthy diet depends on the types of food processing in the diet. You will be given two diets in a random order, determined by a computer:

A. A diet following UK dietary guidance for 8 weeks containing foods based on one type of food processing
B. A diet following UK dietary guidance for 8 weeks containing foods based on another type of food processing

You will be given the diets in a random order, meaning that you could have diet A first then diet B, or diet B first then diet A. We will not tell you which diet you are on. Before the first diet, we will give you healthy eating advice from the Eatwell Guide, which the diets are based on. We will also explain to you about how to report how much of the food provided you have eaten. After the first 8-week diet, we will ask you to return to your normal diet you were eating before for 4 weeks. We will not provide your food or drink during this period. You will then receive the second diet for 8 weeks. After the second 8-week diet, we will stop providing you with food and drink, and you will then need to obtain your own food and drink. We will collect data on blood pressure, body composition, physical activity and fitness, questions regarding quality of life, mental health and

For peer review only - http://bmjopen.bmj.com/site/about/guidelines.xhtml

wellbeing, and blood samples at the start of each diet and at 4 and 8 weeks into each diet. We will work with you to find a suitable period of time to conduct part 1 of the study around any holidays or commitments you have.

The aim of part 1 is to collect important scientific data to see how food processing impacts on our health, rather than to assess specific foods. Therefore, we will provide you with all meals, snacks and drinks for the two 8-week diets, which have been designed with limited flexibility. You will not need to purchase any food or drink during this time. These will be set menus, featuring typical foods recommended in the Eatwell Guide that you might buy from a shop or make yourself at home. The meals and snacks on each menu will be varied across the week and include fruit, vegetables, beef, chicken, pork, fish, bread, potatoes, pasta, rice, noodles, oats, yoghurts, flapjacks, nuts, tea and coffee. We will not provide any alcohol. The diets will be delivered to your home at a time convenient for you or delivered to a safe space every few days. The meals will require minimal preparation, being ready to eat, or needing a few minutes in the microwave. Foods provided will need to be stored in either a fridge, freezer, or cupboard. We will provide a menu guide and instructions on how to prepare your food. Once prepared, you can then eat as little or as much of the food we give you as you like. We will give you kitchen scales and containers to prepare and store food for each day. You will have weekly telephone contact with the research team at a time that is convenient for you to help with any issues with the diets.

To provide you with your food for the diets, you will need to consent to and give your address and contact details to the food suppliers. This is explained in more detail in section 21.

Part 2 of the study will begin after you finish the second 8-week diet. A behavioural scientist will chat with you to create a personal plan to help you to eat a healthier diet and be more physically active. This support will last for 6 months, with ongoing monthly telephone/video calls with the research team to discuss your progress and support you. At the end of the 6 months, we will collect data on blood pressure, body composition, physical activity and fitness, questions regarding quality of life, mental health and wellbeing, and blood samples. We will also invite you to a one-to-one interview to chat about what aspects helped you to eat healthily and be more active, and what aspects made it difficult.

5. How many sessions do I need to attend?

You will be required to attend nine sessions:

- Screening visit

Part 1 of the study:

- Before the start of the first diet
- 4 weeks into the first diet
- 8 weeks into the first diet
- Before the start of the second diet
- 4 weeks into the second diet
- 8 weeks into the second diet

Part 2 of the study:

For peer review only - http://bmjopen.bmj.com/site/about/guidelines.xhtml

- At the start of the behavioural support program (this is a 60-90 minute remote telephone/video call)
- At the end (6 months) of the behavioural support program

Each visit will last 60-90 minutes except for screening, which will last around 30-40 minutes. These sessions will take place at UCLH or at the nearby Centre for Obesity Research (COR). We will conduct the visits at a time that suits you, that fits in with your work schedule and other commitments. If you are selected for MRI scans, this will be an extra 60 minutes at visits 2, 4, 5 and 7.

After all data is collected from visit 9 (the 6-month follow-up for the behavioural support), nothing more will be expected or asked of you, and you will have completed the study.

6. What data will be collected about me?

Screening

At the screening assessment, we will collect information to check that you are eligible and that it is safe for you to take part in the study. This will include:

- Checking that you do not have a medical diagnosis of an eating disorder.
- Checking your medical history to make sure that you do not have any medical conditions which might put you at risk during the study (such as Coeliac disease).
- Checking your current diet to see the amounts of different types of food processing in your diet.
- Checking that you do not have any food allergies or dietary restrictions that make it unsafe or not possible for you to eat the diets that we will provide you in part 1 of the study.
- If you are female, checking that you are not pregnant. We will ask you to take a urine pregnancy test. Urine taken for the pregnancy test will be disposed of following testing and not kept for any reason.

If you are not eligible, your information will be destroyed after 25 years.

During the study

The tests and data we will need to collect will include:

- Blood pressure and heart rate. We will measure blood pressure using a cuff that is placed on your upper arm. Heart rate will be measured with a small clip placed on your finger, called an oximeter.
- Weight and body composition, such as muscle mass and fat mass. You will stand on a scanner that uses an electric current to assess the amount of fat and muscle you have, for 30 seconds. Scales will be used to measure your weight, a stadiometer to measure your height, and a tape measure to measure your waist circumference.
- Blood samples before and after a meal. We will give you a standard meal to eat at our research centre. A 15ml tablespoon sized blood sample will be collected before and 15 minutes and 30 minutes after the meal, which will be performed by a trained healthcare professional. We will measure changes in the hormones that regulate your appetite, as well as markers of metabolism. Blood samples will be taken at five visits: before the start of the

For peer review only - http://bmjopen.bmj.com/site/about/guidelines.xhtml

first and second diet, 8 weeks into the first and second diet, and at the end (6 months) of the behavioural support program. You will need to fast overnight for 12 hours before these five visits.

- How active you are during the day, and how much sleep you are getting. We will ask you to wear a monitor for 7 days to track your movement and sleep. The monitor is worn on your dominant hip or wrist for one week, from waking in the morning until going to bed at night. The monitor can be removed when taking a bath. We need a minimum of four days with at least 10 hours of daily wear time to collect useful data. The activity tracker records only basic movements, and does not record any identifiable information.
- How fit you are. We will ask you to complete three fitness tests. This will include squeezing a handgrip as hard as you can (handgrip strength test), measuring how far you can walk in 6 minutes (six-minute walk test), and testing your ability to stand from a seated position (sit-to-stand test).
- Questionnaires. We will ask you to fill in questionnaires about your physical activity, sleep, mental health, quality of life and eating behaviour, and what made it easier or harder for you to stick with your diet. The questionnaires will be provided to you in an electronic format, and can be completed at the study visit or remotely.
- Dietary information. To keep track of what you are eating, we will ask you to recall what you have eaten in a 24-hour period for two days, and ask you to fill in a questionnaire that asks how frequently you have eaten certain foods. We will explain how to complete these. We will ask you to also take some photos of your prepared meals for a couple of days during the 4- and 8-week follow-up visits in part 1 of the study. If it helps, we can also provide you with a diet diary to keep track of what you eat during each diet.
- A 60-90 minute interview at the end of the study to discuss the 6-month support program. A trained behavioural scientist will chat with you about the aspects of your personal, social and work life that helped you to stick with a healthy diet, and what aspects of your life made it difficult. This interview will be audio recorded and then converted into text. Some of the things you say may be quoted verbatim, but this will be anonymised before being published, so it will not be possible to identify you. Interviews will take place via telephone or video call at a time convenient for the participant during the scheduled visit time. Transcriptions will be anonymised following transcription, and audio recordings deleted as soon as transcriptions of the recordings have been obtained.

For some participants, we will also collect:

- Brain imaging scans. Not all participants will undergo a brain imaging scan. You may be selected to receive a brain scan if you consent, but you can opt out of this on the consent form. You will have a magnetic resonance imaging (MRI) scan on your brain. MRI does not expose you to ionising radiation. Brain imaging will be conducted before you start each diet, and at the 8-week visits for each diet. The scans will last around 60 minutes for each visit, in addition to the 60-90 minute visits. Scans will be conducted at the Institute of Neurology next to Russell Square. We can book these scans around your schedule at a separate visit if necessary. If you do not wish to undergo brain imaging if chosen, you can opt out on the consent form. We will provide you with more information about the brain scans at the screening visit. The brain is important for determining what we eat. Different areas of the brain can impact on the types of foods we eat, and evidence suggests that connections

For peer review only - http://bmjopen.bmj.com/site/about/guidelines.xhtml

For peer review only

between these different areas of the brain may change whilst eating certain diets. We want to learn more about how connections between different brain areas change when eating two different diets.

7. How long will I be involved in the study for?

After signing the written informed consent form, it is expected that your involvement in the study will last for around 49 weeks. Part 1 of the study will last around 24 weeks. Part 2 of the study will last around 25 weeks.

8. Study Timeline

The study timeline is outlined below.

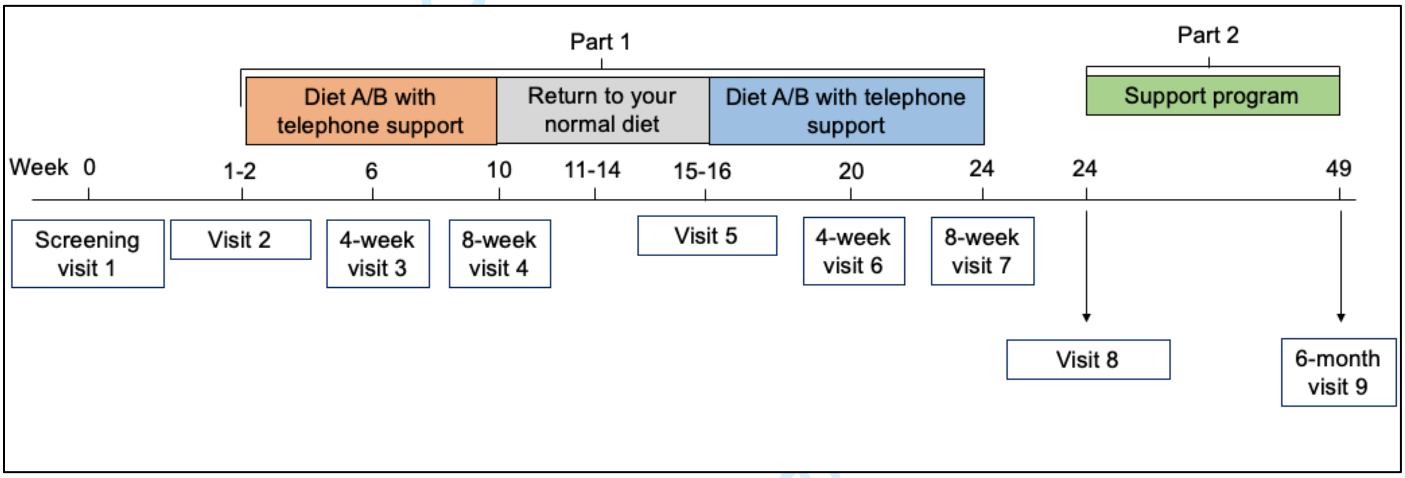

Assessment visit schedule and data collection

The table below outlines the measures collected at each visit and what will happen during the study.

		Part 1						Part 2	
		First diet			Second diet				
	Screening	Before start of diet	At 4 weeks	At 8 weeks	Before start of diet	At 4 weeks	At 8 weeks	At the start of support program	At the end of support program
**Visit Number:**	1	2	3	4	5	6	7	8	9
**Week:**	0	1-2	6	10	15-16	20	24	24	49
Informed Consent for Screening	Y								
Medical history	Y	Y	Y	Y	Y	Y	Y		Y

For peer review only - http://bmjopen.bmj.com/site/about/guidelines.xhtml

Heart rate and blood pressure	Y	Y	Y	Y	Y	Y	Y		Y
Urine Pregnancy Test (females only)	Y								
Body composition		Y	Y	Y	Y	Y	Y		Y
Blood samples before and after a meal: you will need to fast for 12 hours before this visit		Y		Y	Y		Y		Y
Physical activity and sleep monitor		Y		Y	Y		Y		Y
Physical Fitness Tests		Y	Y	Y	Y	Y	Y		Y
Questionnaires		Y	Y	Y	Y	Y	Y		Y
Dietary information	Y	Y	Y	Y	Y	Y	Y		Y
Brain scans (if chosen)		Y		Y	Y		Y		
Behavioural support call								Y	
One to one telephone/video call interview									Y

For peer review only

9. Where will the study take place?

The study will take place at the Centre for Obesity Research (COR) at UCL and at UCLH. If selected for brain scans, these will be at the nearby UCL Institute of Neurology. You will not need to travel to any other location at any other time.

10. What will I have to do?

If enrolled in the study, we will expect you to attend all scheduled ither in-person or remote study visits, and perform the tests and assessments as listed above, accordingly to the study protocol. Importantly, you will be asked to prepare and eat the diets provided to you in part 1, and not to consume other foods or drinks during each 8-week period. The research team will be available if you need to contact them, for example, if there are issues with adherence due to social events,

For peer review only - http://bmjopen.bmj.com/site/about/guidelines.xhtml

issues with the provided diets (e.g. a missing delivery or missing meals) or problems with dietary reporting.

11. What are the possible disadvantages and risks of taking part?

Possible disadvantages and risks are related to the study assessments, procedures and questionnaires you will have to undergo when taking part in the trial. However, these would be considered minor risks/disadvantages, as explained below. The study is generally low risk. We do not expect there to be any major risks or adverse events from taking part in the study. There are no expected risks from the screening visit.

The two 8-week diets that we will provide you in part 1:

- As part of the screening process, we will make sure that you do not have any allergens or dietary restrictions to the foods we will provide you in part 1. We will check that there are no potential concerns regarding your eating behaviour.
- We will provide you with simple instructions on how to safely prepare and store your meals, and you will not be expected to consume foods beyond their best by date or use by date.

Blood samples before and after a meal:

- Taking blood samples may occasionally cause a bruise or pain. All samples will be obtained by a trained healthcare professional.

Physical fitness tests:

- The physical fitness measures are not expected to result in a risk of injury. Trained researchers will provide full instructions and supervision for the tests.

Questionnaires:

- You might find some of the questions about your mental health and quality of life upsetting. However, you can stop the questionnaires at any time, or decline to answer specific questions, without having to give a reason or without affecting your rights. If you do feel upset and you feel you would benefit from some emotional support, you can contact the Samaritans charity (https://www.samaritans.org) on 116 123, their helpline is open 24 hours per day. You can also contact MIND (https://www.mind.org.uk), or the CALM (Campaign against living miserably) charity, who have a helpline open from 5pm to midnight every day on 0800 58 58 58 or a web chat (https://www.thecalmzone.net).

Support program:

- In order to support you with eating a healthy diet and being more physically active, the behavioural scientist may discuss aspects of your personal, social and work life that may be upsetting. The aim of the discussion is to help you, such that there is a positive impact on your health. As with the questionnaires, you can stop the discussions at any time, or decline to answer specific questions, without having to give a reason or without affecting your rights.

For peer review only - http://bmjopen.bmj.com/site/about/guidelines.xhtml

Brain scans:

- If you consent and are selected for brain imaging assessment (unless you choose to opt out on the consent form), you will undergo an MRI scan. MRI does not expose you to ionising radiation. But, the noise from the scanner and small space can cause stress and discomfort.
- Our researchers will check that you are suitable to undergo MRI brain scanning during screening. We will also check for any reasons why you cannot undergo an MRI scan, which will be confirmed prior to every scan.

Unexpected findings

In the unlikely event that we notice an abnormal finding in some of the measurements we take (including blood tests, heart rate, blood pressure and brain scans), we will inform you and your GP, who will discuss the findings and any further action needed with you.

Will I experience any side-effects?

We do not expect there to be any side effects from the diet interventions, from the support program, or from the tests involved.

The diets provided contain typical foods in UK diets and are obtained from supermarkets or made in a professional catering facility.

It is important that if you do feel any unusual symptoms, regardless of whether you think they are linked to the study, that you tell a member of the study team.

12. What are the possible benefits of taking part?

It is likely that you will experience a benefit from the 6-month support program as you will be supported to improve your diet and be more physically active. It is not possible to guarantee any specific benefit from part 1 of the study, where we provide you with two diets.

The knowledge gained from this study will provide further information into whether a healthy diet depends on how processed it is. Results from this study can be used to inform changes to the current recommendations for dietary guidelines. A greater understanding of the long-term risks of diets can better inform healthcare professionals on helping people living with obesity.

13. How many participants will be in the study?

We will recruit 55 members of staff at UCLH living with overweight or obesity. The study will run for approximately 18 months in total.

14. What happens when the trial stops?

We will only provide your food and drink for the two 8-week diets in part 1 of the study. We will not be able to provide behavioural support after the end of the study.

For peer review only - http://bmjopen.bmj.com/site/about/guidelines.xhtml

At the end of the trial, we will analyse the data and publish the results in medical journals, and present results at scientific conferences. If you wish, a copy of the reports on the study findings can be requested from the Chief Investigator, Professor Batterham, at the address given in section 19 of this information sheet. Should the study be stopped prematurely for any reason, we will tell you why.

15. Will my taking part in the study be kept confidential?

Yes. We will follow ethical and legal practices and all information about you will be handled in confidence. The details are outlined in the following section 19.

16. Will I be paid to take part in the study?

You will receive the two 8-week diets for free in part 1 of the study. This means you will not need to purchase any food or drink for 16 weeks. We will not directly pay you for any aspects of the study.

We do not foresee any travel costs, as all study measurements will be at UCLH or the adjacent COR, so you will not have to make any extra journeys or travel anywhere other than to your usual appointment location. If you do need to travel specifically for the study visits, any reasonable travel costs incurred to attend the study visit will be reimbursed on production of valid receipts. All the food we provide will be delivered to your home every few days, so you do not need to travel to collect your food. We will work with you to ensure food deliveries, meetings or assessments align with your work and other commitments.

None of the study team are being paid beyond their normal salary to conduct the research, nor are any clinicians being paid for recruiting individuals into the study.

17. What if relevant new information becomes available?

Sometimes during a clinical trial, new information about the intervention becomes available. If this happens, we will tell you about it and discuss with you whether you want to or should continue your participation in this study. If you decide not to carry on, we will make arrangements for your care to continue. If you decide to continue in the study, you might be asked to sign an updated consent form. Also, on receiving new information, we might consider it to be in your best interest to withdraw from the study. If so, we will explain the reasons and arrange for your care to continue.

18. What will happen if I don't want to carry on with the study?

Your participation in the trial is entirely voluntary and you will be free to withdraw from the study at any time and without giving a reason. A decision to withdraw will not affect the current or future care you receive. If you withdraw from the study, we will destroy all your identifiable information, but we will need to use and analyse the pseudonymised data collected up to the point of your withdrawal. You will be provided with a withdrawal form where you will be able to express your preference. In any case, pseudonymised (coded) information collected up to the point of your

For peer review only - http://bmjopen.bmj.com/site/about/guidelines.xhtml

For peer review only

withdrawal will still be used in the analysis. Any stored blood or tissue samples that can still be identified as yours will be destroyed if you wish.

19. What if there is a problem?

If you have any problem during the study, or would like to discuss any aspect of the study, you can contact any of the research investigators. You can find their contact details on the last page of this information sheet. Every care will be taken in the course of this clinical trial.

However, in the unlikely event that you are injured by taking part, compensation may be available through the University College London (UCL) insurance scheme. If you suspect that the injury is the result of the Sponsor's (University College London) or the hospital's negligence then you may be able to claim compensation. After discussing with your research doctor, please make the claim in writing to the Chief Investigator for this trial:

Professor Rachel Batterham,
Centre for Obesity Research,
Rayne Building,
5 University Street,
London, WC1E 6JF.

The Chief Investigator will then pass the claim to the Sponsor's Insurers, via the Sponsor's office.

However, if you remain unhappy or have a complaint about any aspect of this study and wish to speak to someone independent of the research team/hospital, please contact the Head of Research Governance and Compliance, UCL/UCLH Joint Research Office, University College London, Gower Street, London WC1E 6BT email: research-incidents@ucl.ac.uk.

Regardless of this, if you wish to complain, or have any concerns about any aspect of the way you have been approached or treated by members of staff or about any side effects (adverse events) you may have experienced due to your participation in the clinical trial, the normal National Health Service complaints mechanisms are available to you. You could also write or get in touch with the Complaints Manager, UCL hospitals. Please quote the study IRAS number at the top of this information sheet. Please ask your study doctor if you would like more information on this. Details can also be obtained from the NHS website and the Department of Health website: http://www.dh.gov.uk. You can also contact the UCLH Patient Advise Liaison Service (PALS) via phone: 020 3447 3042 or email: uclh.pals@nhs.net. In case you feel you would benefit from some emotional support, you can contact the Samaritans charity on 116 123, their helpline is open 24 hours per day. The CALM (Campaign against living miserably) charity also have a helpline open from 5pm to midnight every day on 0800 58 58 58 or a web chat (https://www.thecalmzone.net).

20. How will we use information about you?

Data Protection Information

We will need to use information from you and your medical records for this research project. This information will include your contact details [name, initials, telephone number(s), email address, address, NHS/hospital number], demographics [age, gender, marital status, ethnicity, education,

For peer review only - http://bmjopen.bmj.com/site/about/guidelines.xhtml

occupation]), which will be held by UCL. People will use this information to do the research or to check your records to make sure that the research is being done properly. People who do not need to know who you are will not be able to see your name or contact details. Your data will have a code number instead. We will keep all information about you safe and secure. Once we have finished the study, we will keep some of the data so we can check the results. We will write our reports in a way that no-one can work out that you took part in the study.

UCL is the sponsor for this study based in the United Kingdom. The sponsor is the organisation responsible for ensuring that the study is carried out correctly. UCL will act as the data controller for this study. This means that they are responsible for looking after your information and using it properly. UCL will keep this information about you for 25 years after the study has finished. The UCL Data Protection Office provides oversight of UCL activities involving the processing of personal data and can be contacted at data-protection@ucl.ac.uk. Further information on how UCL uses your information can be found on our general research privacy notice here https://www.ucl.ac.uk/legal-services/privacy.

In order to conduct the study, staff outside your normal care will need access to your medical records. You will need to give permission to the study team to access your medical records, and for long-term storage in an anonymised form. All of your personal information will be kept strictly confidential. The study will legally comply with the Data Protection Act, 2018. Your patient records and study information will be stored behind a card-secure door, with access only by members of the research team. Any data that leaves the study sites (UCLH and UCL) will be anonymised. Your name and other identifiable information will not be recorded on records or samples. Your initials and trial identification number will be used on records or samples.

When you agree to take part in a research study, the information about your health and care may be provided to researchers running other research studies in this organisation and in other organisations. These organisations may be universities, NHS organisations or companies involved in health and care research in this country or abroad. Your information will only be used by organisations and researchers to conduct research in accordance with the UK Policy Framework for Health and Social Care Research.

We collect personal data directly from you, or your GP and/or hospital team if needed, for the purposes of carrying out this research study. We use your name, NHS number and contact details to contact you about the research study and make sure that relevant information about the study is recorded for your care, and to oversee the quality of the study. We only share your personal data in limited circumstances, with your GP, as set out in this information sheet, and with individuals from regulatory organisations where they need to look at your medical and research records to check the accuracy of the research study. The only people in UCL who will have access to information that identifies you will be people who need to contact you as part of the study or audit the data collection process. The people who analyse the study data will not be able to identify you and will not be able to find out your name, NHS number or contact details. We will keep all information about you safe and secure. Once we have finished the study, we will keep some of the data so we can check the results. The final study reports will contain anonymised data only and will not contain any identifiable data or personal information.

For peer review only - http://bmjopen.bmj.com/site/about/guidelines.xhtml

Your personal data will be processed for the purposes outlined in this information sheet. The legal basis to process your personal data will be:

- to undertake the research study, on the basis of performance of a task carried out in the public interest and processing your special categories of personal data (e.g., health information) for research purposes.
- where required to make disclosures to your GP further to the results of any study test/questionnaire, on the basis of performance of a task carried out in the public interest, compliance with a legal obligation and processing your special categories of personal data (e.g., health information) for public health purposes.
- to contact you about participating in further research studies, only where you have provided consent on the consent form. You have the right to withdraw your consent to be contacted about future studies at any time, using the details set out at the end of this section.

To deliver the 8-week diets to you, the food suppliers will need your name, contact details and address. We will not share your personal details with the food suppliers. For one of the diets, we will control a secure, password-protected online food delivery account that only the research team can log into. We will ask you to add your name, phone number and address to the account delivery details for the purposes of delivering the diet to you. The supermarket will handle your data according to their privacy policy. We will ask you to add your details to an account for a second supermarket, to be used only if there are issues with delivery from the first supermarket. It is not possible to determine that you are involved in the study from this. When your time on this 8-week diet ends, we will delete your details from the account. For the other diet, you will need to give your name, address and contact details directly to the food supplier. We will give you the details needed to contact them, if you are eligible and decide to participate. You will need to agree to this on the consent form.

Your personal data will be processed only for so long as it is required for the research project. We will pseudonymise the personal data as soon as registered on the study database and endeavour to minimise the processing of personal data, wherever possible, to safeguard your privacy. In the event of any future loss of capacity to make decisions relating to your ongoing participation in this study, you will automatically be withdrawn from the study. The information already collected up to that point, may continue to be used confidentially in connection with this study and future research, if applicable.

In line with the regulations, at the end of the study your data will be securely archived for a minimum of 25 years.

Under data protection law, you have individual rights in relation to the personal data we hold about you. For the purposes of research where such individual rights would seriously impair research outcomes, such rights may be limited. In fact, as we need to manage your records in specific ways for the research to be reliable, we will not be able to let you see or change the data we hold about you. If you would like to exercise a right under data protection law or find out more about how we use your information, please contact data-protection@ucl.ac.uk.

Personal contact details will be removed at the end of the study if you decide to opt out of receiving a results summary.

For peer review only - http://bmjopen.bmj.com/site/about/guidelines.xhtml

We may need to request information about you from your medical records via your GP if we are unable to obtain this from yourself. This information may include age, gender, ethnicity, job occupation, work pattern, educational level, marital status, medication intake, alcohol consumption, smoking habits, family history of obesity and possible related diseases. Any data sent from your GP to the researchers will be transferred using encrypted NHS email systems that are secure for sending confidential information.

21. What are your choices about how your information is used?

- You can stop taking part in the study at any time, without giving a reason, but we will keep information about you that we already have.
- We need to manage your records in specific ways for the research to be reliable. This means that we won't be able to let you see or change the data we hold about you.
- If you agree to take part in this study, you will have the option to take part in future research using your data saved from this study. This is explained in section 24.

22. Where can you find out more about how your information used?

You can find out more about how we use your information:
- at www.hra.nhs.uk/information-about-patients/
- our leaflet available from www.hra.nhs.uk/patientdataandresearch
- by asking one of the research team
- by sending an email to data-protection@ucl.ac.uk, or
- by ringing us on 0203 108 8764

If you remain unsatisfied, you may wish to contact the Information Commissioner's Office (ICO) (https://ico.org.uk/concerns/handling/).

23. Will my GP be informed of my involvement?

With your permission, your GP will be notified that you are taking part in this study. If the study investigators became concerned about your well-being or about the implications of what you tell us for someone else's well-being we would need to inform your GP or other professionals. We would, of course, discuss this with you first. In addition, should your blood test results show any abnormality, we will let your GP know, who will be able to make necessary arrangements for your best care. You will be asked to agree to this when signing the consent form.

24. What will happen to my blood samples?

As part of this trial, we will collect new blood samples from you during the face-to-face visits, as detailed above. These blood samples will be stored and analysed at UCLH and the Centre for Obesity Research, University College London (UCL). Samples will be anonymised, which means they will not be identifiable. The samples will be identified only by your study number and will not be directly marked with your name. The samples will only be used for the study described above, however, with your consent, any remaining blood samples you provided for the study may be

For peer review only - http://bmjopen.bmj.com/site/about/guidelines.xhtml

retained after the end of the study for future research. Only researchers involved in this study will have access to the blood samples. You can choose how you would like your samples to be used after the study ends in the consent form. We can transfer your samples to the Obesity Research Biobank Syndicate (ORBiS) run by COR, where they will be stored in a secure database and used for further research, or they can be safely disposed of. Your samples will be disposed of in accordance with the Human Tissue Act 2004. If you choose to withdraw from the study, any stored blood or tissue samples that can still be identified as yours will be destroyed if you wish.

25. What will happen to the results of the trial?

The results of the study will be available after it finishes and will usually be published in a medical journal or presented at a scientific conference. The data will be anonymised and none of the participants involved in this study will be identified in any report or publication. We will be happy to make the results of the study available to you. You can request a lay summary of the study results by ticking the corresponding box in the consent form.

26. Who is conducting the trial?

The study is being run by the UCL Centre for Obesity Research who care for patients living with obesity.

27. Who is organising and funding the trial?

This research study is being organised by the Centre for Obesity Research, University College London (UCL). The Chief Investigator is Professor Rachel L. Batterham (Consultant Obesity Physician, Diabetologist, and Endocrinologist) who has vast experience in clinical research studies. This study is being sponsored by University College London (UCL) and being funded by the National Institute for Health Research (NIHR) and Rosetrees trust.

28. How have patients and the public been involved in this trial?

In designing and planning this study, we have taken into account the opinions of UCLH staff following a focus group discussion, who have reviewed and given feedback on the study design and this Information Sheet.

29. Who has reviewed the trial?

It is important that when we carry out research, we make sure the study design is as good as possible to reduce the risk of bias and poor-quality research. All research in the NHS is looked at by independent group of people, called a Research Ethics Committee, to protect your interests. This study has been reviewed and given favourable opinion by the Yorkshire & The Humber - Sheffield Research Ethics Committee. Academics from UCL, National Institutes of Health in the US and academic peer-reviewers have also evaluated and reviewed the study design.

30. Further information and contact details

For peer review only - http://bmjopen.bmj.com/site/about/guidelines.xhtml

If you require any further information or have any concerns while taking part in the study, please contact one of the following people:

Samuel Dicken Phone: 07415690476 Email: samuel.dicken.20@ucl.ac.uk

Fred Jassil Email: friedrich.jassil.13@ucl.ac.uk

If you decide you would like to take part, then please read and sign the consent form. You will be given a copy of this information sheet and the consent form to keep. A copy of the consent form will be filed in your patient notes, one will be filed with the study records and one may be sent to the Research Sponsor.

You can have more time to think this over if you are at all unsure.

Thank you for taking the time to read this information sheet and for considering this study.

For peer review only

For peer review only - http://bmjopen.bmj.com/site/about/guidelines.xhtml

CONSENT FORM

Centre for Obesity Research
Division of Medicine
University College London
Rayne Building
5 University Street
London WC1E 6JF
Telephone: 020 7679 0788

Email: samuel.dicken.20@ucl.ac.uk
r.batterham@ucl.ac.uk

UPDATE trial: A study comparing the health effects of two diets following UK dietary guidance in people living with overweight or obesity.

Name of Chief Investigator: Professor Rachel L Batterham **Participant screening number:** [ ]

Sponsor Protocol Number: 151582
IRAS ID number: 311525

Thank you for considering taking part in this project. If you have any questions arising from the information sheet or explanation already given to you, please ask the research team before you decide whether to join, contact details are on the top right-hand side of this page. This consent form is to confirm that you are happy to undergo the screening assessments to participate in the study, and if eligible, to participate in the study. For some elements, you must circle an option (e.g., consent/do not consent). You must circle one option and initial the box to confirm your selection. Leaving any boxes not initialled will result in the consent form being incomplete and not valid.

Please initial box

1	I confirm that I have read the participant information sheet (Version 1.1, dated 12.12.2022), which explains the benefits and risks of taking part in the study.	
2	I confirm that I have been provided with sufficient information and opportunity to ask any questions about the study and have had these answered satisfactorily.	
3	I confirm that I have been informed about any risk that may result from taking part in this study and that I have had sufficient time to consider whether or not I want to take part.	
4	I confirm that I understand the requirements for participating in the study, and that I am able to attend all relevant study sessions and procedures as outlined in the participant information sheet (Version 1.1, dated 12.12.2022).	
5	I understand that my participation in this study is voluntary. It is my right to withdraw from the study at any time, with no penalties. I do not need to give a reason for doing so. This will not affect my standard care or my legal rights.	
6	I understand that relevant sections of my medical notes and data collected during the study may be looked at by individuals from the sponsor of the trial (University College	

For peer review only - http://bmjopen.bmj.com/site/about/guidelines.xhtml

	London) and responsible persons authorised by the sponsor, from regulatory authorities or from the NHS Trust, where it is relevant to my taking part in this research. I give permission for these individuals to have access to my records. I understand that such information will be handled appropriately and confidentially.	
7	I understand that the UCL research team will access parts of my medical record and my data collected in the study. I give permission for this access.	
8	I understand that my name, contact details and address need to be used for the purposes of delivering the diets to my home at a time that is convenient for me, as outlined in the participant information sheet (Version 1.1, dated 12.12.2022). The UCL research team will not share my details with the food suppliers. I agree that I will provide my contact details and address to the food suppliers to deliver the diets.	
9	I give permission for a researcher to contact me for the purposes of offering support during the interventions, ensuring the diet deliveries are received, in collecting study samples and to remind me to complete the study measurements.	
10	I give permission for my anonymised data to be used in future publications related to this study and to be stored securely for up to 25 years after the end of the study.	
11	I acknowledge that UCL will handle my personal data for the purposes of this research study and I have been provided with information about how UCL handles personal data, as described in the participant information sheet (Version 1.1, dated 12.12.2022).	
12	I understand that my GP will be informed that I am participating in this study.	
13	I consent that in the event that I lose the capacity to make decisions relating to my ongoing participation in this study, I will automatically be withdrawn from the study. The information already collected up to that point may continue to be used confidentially in connection with this study and future research, if applicable, as described in the participant information sheet (Version 1.1, dated 12.12.2022).	
14	I agree to the one-to-one interview at the 6-month follow-up to be recorded for data analysis purposes, and I understand that some of the things I say may be quoted verbatim in publications without me being directly identified.	
15	I consent / do not consent (please circle) to being potentially chosen to have the MRI brain scans as described in the participant information sheet (Version 1.1, dated 12.12.2022).	
16	I give permission for my blood samples to be stored and analysed at the University sites.	
17	At the end of the study, I consent / do not consent (please circle) to having my blood samples stored in ORBiS for future research as explained in the participant information sheet (Version 1.1, dated 12.12.2022). If I do not agree, I understand that my samples will be disposed of according to Human Tissue Act 2004.	
18	I consent / do not consent (please circle) for my personal information to be retained and used to contact me for the purpose of participating in future research.	

For peer review only - http://bmjopen.bmj.com/site/about/guidelines.xhtml

19	I would / would not (please circle) like to receive a summary of the results.	
20	I voluntarily consent and agree to undergoing a screening assessment to determine my eligibility for the study, and if eligible, participating in the above study.	

--------------------------------- ---------------------------- ----------------------

Name of participant Signature Date

I confirm that I have explained the study to the above participant and have answered questions honestly and fully.

--------------------------------- ---------------------------- ----------------------

Name of investigator taking consent Signature Date

Comments or concerns during the study

If you have any comments or concerns, you may discuss these with the investigators. If you wish to go further and complain about any aspect of the way you have been approached or treated during the course of the study, you should write or get in touch with the Complaints Manager, UCL Hospitals. Please quote the study number on the front page of this consent form.

When completed: 1 (original) for participant; 1 (original) for researcher site file; 1 to be kept in medical notes.

For peer review only - http://bmjopen.bmj.com/site/about/guidelines.xhtml

Supplementary Materials: Contraception list

Female participants

Females of childbearing potential are eligible to participate if they agree to use a highly effective contraception method for the duration of the trial and until 6 weeks after treatment discontinuation. Women are considered of childbearing potential following menarche and until becoming post-menopausal unless permanently sterile. Women are considered permanently sterile if they have had documented hysterectomy, bilateral salpingectomy or bilateral oophorectomy. Postmenopausal state is defined as no menses for 12 months without any medical cause. A high Follicle Stimulating Hormone (FSH) level in the postmenopausal range may be used to confirm a postmenopausal state in women not using hormonal contraception or Hormonal Replacement Therapy (HRT). However in the absence of 12 months of amenorrhoea, a single FSH measurement is insufficient.

Highly effective contraceptive methods include:

- Combine (oestrogen and progesterone containing) hormonal contraception associated with inhibition of ovulation:
  - Oral
  - Intravaginal
  - Transdermal
- Progesterone-only hormonal contraception associated with inhibition of ovulation:
  - Oral
  - Injectable
  - Implantable
- Intrauterine device.
- Intrauterine hormone-releasing system.
- Bilateral tubal occlusion.
- Vasectomised partner.
- True sexual abstinence (refraining from sexual intercourse – only acceptable when this is in line with the preferred and usual lifestyle of the subject).

Male participants

Male participants with partners of childbearing potential should use barrier methods of contraception for the duration of the trial until 6 weeks after treatment discontinuation.

For peer review only

UPDATE Exit Interview Guide

Full details of the intervention development and content will be provided in a future publication focussed on the behavioural support intervention. This document outlines the support programme exit interview. This guide is to ensure that key aspects of participants' opinions of participating in the trial are covered during the interview. This is a semi-structured interview guide, and as such, a respondent-sensitive approach should be taken, allowing deviation from question order and raising additional issues if appropriate, depending on the conversation with each individual.

Introduction:

Introduce yourself and explain that the purpose of the call is to find out more about how they have found participating in the behavioural support programme. Ask participants to be as open and honest as possible in their answers and reassure that there are no right or wrong answers – we're just interested in their opinions. Ask permission to record the phone call so that we can come back to their answers.

When you first saw the advert for the UPDATE trial, what made you want to take part? Was the offer of behavioural support appealing to you? *[Prompt on reasons/what they hoped to get out of the behavioural support]*

I will ask about individual parts of the programme in more detail later, but overall, did you feel you got any benefit from the behavioural support? [*Prompt on what this was/reasons why/why not]*

When you first entered the trial, you weren't told it was about ultra-processed food (UPF) / minimally processed food (MPF). How did you feel when you found this out? [*Prompt on reasons/whether this something they felt positively or negatively about]*

Were you familiar with the ideas of UPF or MPF before you started this programme? Do you feel you understand these more as a result of taking part?

[If they were aware of UPF/MPF before the trial] How worthwhile did you think it was to reduce UPF/increase MPF before you started this programme? *[Probe on reasons for it being worthwhile versus not]*

How worthwhile to do you feel it is to reduce UPF/increase MPF now? *[Probe on reasons]*

I am now going to ask about a different parts of the behavioural support programme. I want to know what worked for you, but also really want to know what didn't work. If there was anything you didn't use it's also really helpful for us to know.

[Note to researcher – these sections do not have to be in order. If it feels more natural to start talking about another part of the programme, i.e. because a participant raises it, then be led by them]

Let's talk about:

For peer review only - http://bmjopen.bmj.com/site/about/guidelines.xhtml

The **behavioural support calls/sessions** [*Prompt on how many they received and if they did not receive all, ask why they missed some. Researcher should be aware in advance of which calls/sessions they received and can use this to help remind them if needed].*

- Ask if they did it online/in person and reasons for preference. [Use 'sessions' rather than calls if they were in person].
- What were your feelings about the calls/sessions overall? Were they helpful?
- Let's break it down and think about the first one. That was the **introductory call/session** where you were asked about your experience of diet A/B, what influenced how you ate before joining the trial, personal barriers, and were asked to fill in a food/mood diary. Did you find this call useful? *[Probe on what they liked/didn't like, the length/the areas covered/the diary/what could have been done differently]*
- Let's think about the call you had a week later (the **first behavioural support call/session**). Did you find this call useful? *[Probe on what they liked/didn't like, the length/the areas covered/the diary/what could have been done differently]*
- In the month three call physical activity was introduced. How did you feel about this? *[Probe on what they liked/didn't like, the length/the areas covered/the diary/what could have been done differently]*
- What about the other (check-in) calls in-between these? Were they useful? [*Prompt on reasons]*
- Overall, how did you feel about the frequency of calls/sessions – was it too little/too much/about right for you? *[Prompt on reasons and what they might have preferred where relevant]*
- Still thinking about the calls/sessions - do you have any suggestions for what we could do differently in future?

In between the calls you got **weekly emails**. How did you feel about these? Did you read them? Were they helpful? Was weekly OK for you? What about the content of the emails? Was this a good way to communicate with you between calls/sessions? *[Probe on other preferences if not].*

Did you use the **UPDATE behavioural support** (green) booklet alongside your behavioural support calls?

- If no, explore why not.
- If yes:
  - What were your feelings about it overall? Was it helpful to use during the call?
  - How did you feel about the design/content?
  - Did you refer to it in between the calls? *(Probe on why/why not)*
  - Do you still refer to it now?
  - Did your interest in or use of the booklet change over the course of the programme? *[Probe on how/why]*
  - Do you think the booklet influenced your UPF/MPF intake? *(Probe on why/why not)*
  - Do you think it influenced your physical activity? *(Probe on why/why not)*
  - Is there anything you would add or change in the green booklet?

For peer review only - http://bmjopen.bmj.com/site/about/guidelines.xhtml

Did you use the **UPDATE tracker** (blue) booklet?

- If no, explore why not.
- If yes:
  - What were your feelings about it overall?
  - How did you feel about the design/content?
  - Did you use it more than once?
  - Did your interest in or use of it change over the course of the programme? *[Probe on how/why]*
  - Do you think it influenced your UPF/MPF intake? *(Probe on why/why not)*
  - Do you think it influenced your physical activity? *(Probe on why/why not)*
  - How did you feel about the idea of tracking and setting goals?
  - As we talked about during the programme tracking and setting goals is important. Do you think this was the best way to do it? Do you have ideas of different ways that might work in this type of programme?

Did you use the **UPDATE website**?

- If no, explore why not.
- If yes:
  - What were your feelings about it overall? Was it helpful?
  - How did you feel about the design/content?
  - Did you use it more than once? *[Prompt on how often if so]*
  - What prompted you to use it?
  - Did you use the food mapper/recipes/other resources [*Probe on what they liked and didn't like/what could be improved on each]*
  - Did your interest in or use of the website change over the course of the programme? *[Probe on how/why]*
  - Do you think anything on the website influenced your UPF/MPF intake? (Probe on why/why not)
  - Do you think anything on the website influenced your physical activity?
  - Is there anything you would add or change?

Did you attend any **Group Sessions**?

- If no, explore why not.
- If yes:
  - How many did you attend?
  - What were your feelings about them overall? Were they helpful? Any sessions that stood out as especially helpful/relevant to you? *[Probe as needed]*
  - How did you feel about the format/length?
  - Is there anything you would add or change?

Summary

- Overall, would you recommend the behavioural support programme to other people? *[Probe on reasons why/why not]*

For peer review only - http://bmjopen.bmj.com/site/about/guidelines.xhtml

- You were offered the behavioural support programme as part of the larger study. Would you have taken part if only the behavioural support programme had been offered?
- Do you think having done the first part of the trial had any impact on how you felt about the behavioural support programme? *(Probe on what/why)*
- Have you felt anything has changed for you as a result of taking part, positively or negatively? *(Prompt: noticed any physical/psychological changes, changes in thoughts/feelings, wellbeing?)*
- Did your feelings about the behavioural support programme change from when you first started it to now? *(Probe on how/why)*
- Is there anything you learned during the programme that you are still doing now?
- Is there anything that was missing for you that you haven't told us about?
- Thinking about the study as a whole (both parts) – how did you feel about the assessments *(probe on number, type)* – is there anything else you'd like to say about the way the study was designed or set up?
- Is there anything else you would like to say, that we've not already spoken about?

Thank participant for their time and participation in the interview, and the study as a whole.

For peer review only - http://bmjopen.bmj.com/site/about/guidelines.xhtml

Supplementary Materials: Schedule of Assessments

			First diet intervention				Second diet intervention		Behavioural support program	
	Screening	Baseline Assessment first diet	4-week follow-up first diet	8-week follow-up first diet	4-week washout period	Baseline Assessment second diet	4 -week follow-up second diet	8-week follow-up second diet	Behavioural support counselling	6-month follow-up
**Visit No:**	**1**	**2**	**3**	**4**		**5**	**6**	**7**	**8**	**9**
**Week**	**0**	**1-2**	**6**	**10**	**11-14**	**15-16**	**20**	**24**	**24**	**49**
Informed Consent	✓[a]									
Concomitant Medication review	✓	✓	✓	✓		✓	✓	✓		✓
Adverse Events review		✓	✓	✓		✓	✓	✓		✓
Urine Pregnancy Test	✓									
Socio-demographics	✓									
Medical History and Co-morbidities	✓	✓	✓	✓		✓	✓	✓		✓
Vital Signs	✓	✓	✓	✓		✓	✓	✓		✓
Randomisation[b] for diet order		✓								
Height	✓									
Weight	✓	✓	✓	✓		✓	✓	✓		✓
Waist circumference		✓	✓	✓		✓	✓	✓		✓
BIA		✓	✓	✓		✓	✓	✓		✓
Blood sample collection		✓		✓		✓		✓		✓
Meal test		✓		✓		✓		✓		✓
Accelerometry (ActiGraph)		✓		✓		✓		✓		✓
Physical activity and sleep questionnaires		✓	✓	✓		✓	✓	✓		✓
Physical Function Assessment (6MWT, STS, HGST)		✓	✓	✓		✓	✓	✓		✓
Mental health and wellbeing Questionnaires		✓	✓	✓		✓	✓	✓		✓
Eating behaviour Questionnaires		✓	✓	✓		✓	✓	✓		✓
COM-B questionnaire		✓								✓
Behavioural support program									✓	
One to one semi-structured phone/video call interview										✓

For peer review only

Supplementary Materials: Schedule of Assessments

Diet assessment: 24-hr recall (Intake24)	✓	✓	✓	✓		✓	✓	✓		✓
Diet assessment: FFQ (EPIC-Norfolk FFQ)		✓	✓	✓		✓	✓	✓		✓
Diet assessment: IBDA			✓	✓			✓	✓		
MRI brain scans in a subset		✓		✓		✓		✓		

[a] Prior to Screening.

[b] Randomisation will be undertaken after the participant has completed the baseline diet assessment.

Abbreviations: BIA: Bioelectrical Impedance Analysis; COM-B: capability, opportunity, motivation – behaviour; FFQ: food frequency questionnaire; HGST: handgrip strength test; IBDA: image-based dietary assessment; MRI: magnetic resonance imaging; STS-test: sit-to-stand test; 6MWT: 6-minute walk test.

For peer review only

SPIRIT

STANDARD PROTOCOL ITEMS: RECOMMENDATIONS FOR INTERVENTIONAL TRIALS

SPIRIT 2013 Checklist: Recommended items to address in a clinical trial protocol and related documents*

Section/item	ItemNo	Description	
**Administrative information**			
Title	1	Descriptive title identifying the study design, population, interventions, and, if applicable, trial acronym	Y
Trial registration	2a	Trial identifier and registry name. If not yet registered, name of intended registry	Y
	2b	All items from the World Health Organization Trial Registration Data Set	
Protocol version	3	Date and version identifier	Y
Funding	4	Sources and types of financial, material, and other support	Y
Roles and responsibilities	5a	Names, affiliations, and roles of protocol contributors	Y
	5b	Name and contact information for the trial sponsor	Y
	5c	Role of study sponsor and funders, if any, in study design; collection, management, analysis, and interpretation of data; writing of the report; and the decision to submit the report for publication, including whether they will have ultimate authority over any of these activities	Y
	5d	Composition, roles, and responsibilities of the coordinating centre, steering committee, endpoint adjudication committee, data management team, and other individuals or groups overseeing the trial, if applicable (see Item 21a for data monitoring committee)	Y
**Introduction**			
Background and rationale	6a	Description of research question and justification for undertaking the trial, including summary of relevant studies (published and unpublished) examining benefits and harms for each intervention	Y

For peer review only

	6b	Explanation for choice of comparators	Y
Objectives	7	Specific objectives or hypotheses	Y
Trial design	8	Description of trial design including type of trial (eg, parallel group, crossover, factorial, single group), allocation ratio, and framework (eg, superiority, equivalence, noninferiority, exploratory)	Y
**Methods: Participants, interventions, and outcomes**			
Study setting	9	Description of study settings (eg, community clinic, academic hospital) and list of countries where data will be collected. Reference to where list of study sites can be obtained	Y
Eligibility criteria	10	Inclusion and exclusion criteria for participants. If applicable, eligibility criteria for study centres and individuals who will perform the interventions (eg, surgeons, psychotherapists)	Y
Interventions	11a	Interventions for each group with sufficient detail to allow replication, including how and when they will be administered	Y
	11b	Criteria for discontinuing or modifying allocated interventions for a given trial participant (eg, drug dose change in response to harms, participant request, or improving/worsening disease)	Y
	11c	Strategies to improve adherence to intervention protocols, and any procedures for monitoring adherence (eg, drug tablet return, laboratory tests)	Y
	11d	Relevant concomitant care and interventions that are permitted or prohibited during the trial	Y
Outcomes	12	Primary, secondary, and other outcomes, including the specific measurement variable (eg, systolic blood pressure), analysis metric (eg, change from baseline, final value, time to event), method of aggregation (eg, median, proportion), and time point for each outcome. Explanation of the clinical relevance of chosen efficacy and harm outcomes is strongly recommended	Y
Participant timeline	13	Time schedule of enrolment, interventions (including any run-ins and washouts), assessments, and visits for participants. A schematic diagram is highly recommended (see Figure)	Y

For peer review only - http://bmjopen.bmj.com/site/about/guidelines.xhtml

Sample size	14	Estimated number of participants needed to achieve study objectives and how it was determined, including clinical and statistical assumptions supporting any sample size calculations	Y
Recruitment	15	Strategies for achieving adequate participant enrolment to reach target sample size	Y
**Methods: Assignment of interventions (for controlled trials)**			
Allocation:			
Sequence generation	16a	Method of generating the allocation sequence (eg, computer-generated random numbers), and list of any factors for stratification. To reduce predictability of a random sequence, details of any planned restriction (eg, blocking) should be provided in a separate document that is unavailable to those who enrol participants or assign interventions	Y
Allocation concealment mechanism	16b	Mechanism of implementing the allocation sequence (eg, central telephone; sequentially numbered, opaque, sealed envelopes), describing any steps to conceal the sequence until interventions are assigned	Y
Implementation	16c	Who will generate the allocation sequence, who will enrol participants, and who will assign participants to interventions	Y
Blinding (masking)	17a	Who will be blinded after assignment to interventions (eg, trial participants, care providers, outcome assessors, data analysts), and how	Y
	17b	If blinded, circumstances under which unblinding is permissible, and procedure for revealing a participant's allocated intervention during the trial	Y
**Methods: Data collection, management, and analysis**			
Data collection methods	18a	Plans for assessment and collection of outcome, baseline, and other trial data, including any related processes to promote data quality (eg, duplicate measurements, training of assessors) and a description of study instruments (eg, questionnaires, laboratory tests) along with their reliability and validity, if known. Reference to where data collection forms can be found, if not in the protocol	Y

For peer review only - http://bmjopen.bmj.com/site/about/guidelines.xhtml

	18b	Plans to promote participant retention and complete follow-up, including list of any outcome data to be collected for participants who discontinue or deviate from intervention protocols	Y
Data management	19	Plans for data entry, coding, security, and storage, including any related processes to promote data quality (eg, double data entry; range checks for data values). Reference to where details of data management procedures can be found, if not in the protocol	Y
Statistical methods	20a	Statistical methods for analysing primary and secondary outcomes. Reference to where other details of the statistical analysis plan can be found, if not in the protocol	Y
	20b	Methods for any additional analyses (eg, subgroup and adjusted analyses)	Y
	20c	Definition of analysis population relating to protocol non-adherence (eg, as randomised analysis), and any statistical methods to handle missing data (eg, multiple imputation)	Y
**Methods: Monitoring**			
Data monitoring	21a	Composition of data monitoring committee (DMC); summary of its role and reporting structure; statement of whether it is independent from the sponsor and competing interests; and reference to where further details about its charter can be found, if not in the protocol. Alternatively, an explanation of why a DMC is not needed	Y
	21b	Description of any interim analyses and stopping guidelines, including who will have access to these interim results and make the final decision to terminate the trial	Y
Harms	22	Plans for collecting, assessing, reporting, and managing solicited and spontaneously reported adverse events and other unintended effects of trial interventions or trial conduct	Y
Auditing	23	Frequency and procedures for auditing trial conduct, if any, and whether the process will be independent from investigators and the sponsor	Y
**Ethics and dissemination**			

Research ethics approval	24	Plans for seeking research ethics committee/institutional review board (REC/IRB) approval	Y
Protocol amendments	25	Plans for communicating important protocol modifications (eg, changes to eligibility criteria, outcomes, analyses) to relevant parties (eg, investigators, REC/IRBs, trial participants, trial registries, journals, regulators)	Y
Consent or assent	26a	Who will obtain informed consent or assent from potential trial participants or authorised surrogates, and how (see Item 32)	Y
	26b	Additional consent provisions for collection and use of participant data and biological specimens in ancillary studies, if applicable	Y
Confidentiality	27	How personal information about potential and enrolled participants will be collected, shared, and maintained in order to protect confidentiality before, during, and after the trial	Y
Declaration of interests	28	Financial and other competing interests for principal investigators for the overall trial and each study site	Y
Access to data	29	Statement of who will have access to the final trial dataset, and disclosure of contractual agreements that limit such access for investigators	Y
Ancillary and post-trial care	30	Provisions, if any, for ancillary and post-trial care, and for compensation to those who suffer harm from trial participation	Y
Dissemination policy	31a	Plans for investigators and sponsor to communicate trial results to participants, healthcare professionals, the public, and other relevant groups (eg, via publication, reporting in results databases, or other data sharing arrangements), including any publication restrictions	Y
	31b	Authorship eligibility guidelines and any intended use of professional writers	Y
	31c	Plans, if any, for granting public access to the full protocol, participant-level dataset, and statistical code	Y

Appendices

For peer review only

Informed consent materials	32	Model consent form and other related documentation given to participants and authorised surrogates	Y
Biological specimens	33	Plans for collection, laboratory evaluation, and storage of biological specimens for genetic or molecular analysis in the current trial and for future use in ancillary studies, if applicable	In manuscript

*It is strongly recommended that this checklist be read in conjunction with the SPIRIT 2013 Explanation & Elaboration for important clarification on the items. Amendments to the protocol should be tracked and dated. The SPIRIT checklist is copyrighted by the SPIRIT Group under the Creative Commons "Attribution-NonCommercial-NoDerivs 3.0 Unported" license.

For peer review only
